# Evaluation of the efficacy and safety of TREM-1 inhibition with nangibotide in patients with COVID-19 receiving respiratory support: the ESSENTIAL randomised, double-blind trial

**DOI:** 10.1016/j.eclinm.2023.102013

**Published:** 2023-05-31

**Authors:** Bruno François, Simon Lambden, Jean-Jacques Garaud, Marc Derive, Jean-Marie Grouin, Pierre Asfar, Cédric Darreau, Jean-Paul Mira, Jean-Pierre Quenot, Jérémie Lemarié, Emmanuelle Mercier, Jean-Claude Lacherade, Christophe Vinsonneau, Tom Fivez, Julie Helms, Julio Badie, Mitchell Levy, Valérie Cuvier, Margarita Salcedo-Magguilli, Anne-Lise Laszlo-Pouvreau, Pierre-François Laterre, Sébastien Gibot, ESSENTIAL investigators

**Affiliations:** a Medical-Surgical ICU Department and Inserm CIC1435 & UMR1092, CHU Dupuytren, Limoges, France; b Victor Phillip Dahdaleh Heart and Lung Research Institute, University of Cambridge, Cambridge, UK; c Inotrem SA, Paris, France; d Université de Rouen, 76821 Mont Saint-Aignan, France; e Department of Intensive Care, CHU d’Angers, France; f Department of Intensive Care, CHU Le Mans, France; g Department of Intensive Care, Groupe Hospitalier Cochin, Paris, France; h Department of Intensive Care, Burgundy University Hospital, Dijon, France; i Department of Intensive Care, Hôtel Dieu, Nantes, France; j Department of Intensive Care, CHRU Tours Hôpital Bretonneau, Tours, France; k Department of Intensive Care, Centre Hospitalier Départemental de Vendée, La Roche-Sur-Yon, France; l Department of Intensive Care, Centre Hospitalier de Béthune, France; m Department of Intensive Care, Ziekenhuis Oost-Limburg, Genk, Belgium; n Department of Intensive Care, Hôpitaux Universitaires de Strasbourg, Nouvel Hôpital Civil, Université de Strasbourg (UNISTRA), Faculté de Médecine and Inserm UMR 1260, RNM, FMTS, Strasbourg, France; o Department of Intensive Care, Hôpital Nord Franche-Comté, Trevenans, France; p Division of Pulmonary, Critical Care and Sleep Medicine, Department of Medicine, Warren Alpert School of Medicine at Brown University, Providence, RI, USA; q Department of Critical Care Medicine, CHR Mons-Hainaut, Mons, Belgium; r Intensive Care Unit, Centre Hospitalier Regional Universitaire (CHRU), 54000 Nancy, France

**Keywords:** COVID-19, TREM-1, Clinical trial, SARS-CoV-2, ICU, Pneumonia, ARDS

## Abstract

**Background:**

Activation of the TREM-1 pathway is associated with outcome in life threatening COVID-19. Data suggest that modulation of this pathway with nangibotide, a TREM-1 modulator may improve survival in TREM-1 activated patients (identified using the biomarker sTREM-1).

**Methods:**

Phase 2 double-blind randomized controlled trial assessing efficacy, safety, and optimum treatment population of nangibotide (1.0 mg/kg/h) compared to placebo. Patients aged 18–75 years were eligible within 7 days of SARS-CoV-2 documentation and within 48 h of the onset of invasive or non-invasive respiratory support because of COVID-19-related ARDS. Patients were included from September 2020 to April 2022, with a pause in recruitment between January and August 2021. Primary outcome was the improvement in clinical status defined by a seven-point ordinal scale in the overall population with a planned sensitivity analysis in the subgroup of patients with a sTREM-1 level above the median value at baseline (high sTREM-1 group). Secondary endpoints included safety and all-cause 28-day and day 60 mortality. The study was registered in EudraCT (2020-001504-42) and ClinicalTrials.gov (NCT04429334).

**Findings:**

The study was stopped after 220 patients had been recruited. Of them, 219 were included in the mITT analysis. Nangibotide therapy was associated with an improved clinical status at day 28. Fifty-two (52.0%) of patients had improved in the placebo group compared to 77 (64.7%) of the nangibotide treated population, an odds ratio (95% CI) for improvement of 1.79 (1.02–3.14), p = 0.043. In the high sTREM-1 population, 18 (32.7%) of placebo patients had improved by day 28 compared to 26 (48.1%) of treated patients, an odds ratio (95% CI) of 2.17 (0.96–4.90), p = 0.063 was observed. In the overall population, 28 (28.0%) of placebo treated patients were not alive at the day 28 visit compared to 19 (16.0%) of nangibotide treated patients, an absolute improvement (95% CI) in all-cause mortality at day 28, adjusted for baseline clinical status of 12.1% (1.18–23.05). In the high sTREM-1 population (n = 109), 23 (41.8%) of patients in the placebo group and 12 (22.2%) of patients in the nangibotide group were not alive at day 28, an adjusted absolute reduction in mortality of 19.9% (2.78–36.98). The rate of treatment emergent adverse events was similar in both placebo and nangibotide treated patients.

**Interpretation:**

Whilst the study was stopped early due to low recruitment rate, the ESSENTIAL study demonstrated that TREM-1 modulation with nangibotide is safe in COVID-19, and results in a consistent pattern of improved clinical status and mortality compared to placebo. The relationship between sTREM-1 and both risk of death and treatment response merits further evaluation of nangibotide using precision medicine approaches in life threatening viral pneumonitis.

**Funding:**

The study was sponsored by Inotrem SA.

**Research in context**

**Evidence before this study**

The treatment of patients with life threatening ARDS caused by SARS-CoV-2 infection has been shown to be amenable to immune modulation therapy, although in spite of positive trials, mortality remains unacceptably high in this population. Observational data supports a potential role for TREM-1 in the pathophysiology of severe COVID-19 and ARDS in general. However to date, no studies have explored the impact of a TREM-1 modulation strategy on outcome in patients.

**Added value of this study**

This study shows for the first time that a TREM-1 modulation strategy with nangibotide may result in improved clinical status and survival at day 28 with a consistent pattern at day 60 in patients with severe COVID-19 compared to placebo. In addition, nangibotide administration appears to be safe and well tolerated in this population.

**Implications of all the available evidence**

These data, combined with existing observational data, support the progression to definitive evaluation of the efficacy of TREM-1 modulation with nangibotide in patients with severe COVID-19 and the wider group of patients with moderate to severe ARDS.

## Introduction

Coronavirus disease 2019 (COVID-19) arises as a consequence of infection with severe acute respiratory syndrome coronavirus 2 (SARS-CoV-2). The clinical spectrum of this disease is broad with most patients experiencing mild or moderate symptoms. However in the subgroup of patients that develop severe disease, the mortality remains high and additional therapies are required.^1^

The Triggering Receptor Expressed on Myeloid Cells 1 (TREM-1) is an immunomodulatory receptor expressed on innate immune cells, endothelial cells, and platelets. The biological function of TREM-1 is the amplification and the maintenance of innate immune reaction initiated by TLR activation.^2^ In patients, the plasma levels of sTREM-1 reflect the level of activation of the TREM-1 pathway.^2^ Nangibotide is a 12 amino-acid peptidic fragment derived from TREM-Like Transcript-1 (TLT-1), a receptor protein belonging to the TREM-1 family. Nangibotide binds the TREM-1 agonist ligand and thereby modulates the amplification of the immune response caused by the activation of the TREM-1 pathway in acute inflammation.^3^

There is a burgeoning evidence base that in patients with COVID-19 pneumonitis, a dysregulated immune state is responsible, at least in part, for the development of severe disease as evidenced by the therapeutic benefits associated with corticosteroid therapy and anti-IL-6 approaches.^4^^,^^5^ Observational data confirm that the TREM-1 pathway is activated and associated with subsequent disease severity, prolonged duration of mechanical ventilation and outcome,^6^, ^7^, ^8^ in COVID-19 and non COVID-19 ARDS.^2^^,^^9^, ^10^, ^11^

In addition, rodent and porcine models demonstrate the protective effect of nangibotide on lung injury in the context of inhaled lipopolysaccharide^12^, ^13^, ^14^ and septic shock animal models.^3^^,^^15^, ^16^, ^17^

Nangibotide has been shown to be safe and well tolerated in both healthy volunteers in doses up to 6 mg/kg/h and patients with septic shock at doses up to 3 mg/kg/h.^18^^,^^19^ Furthermore, following a supportive phase 2a,^19^ the phase 2b ASTONISH study in patients with septic shock demonstrated that TREM-1 modulation with nangibotide in patients with elevated levels of sTREM-1 lead to a pattern of improved acute morbidity.^20^

Here we present the results of the ESSENTIAL study, a randomized controlled trial assessing the safety and tolerability of nangibotide in patients with COVID-19 and hypoxic respiratory failure receiving respiratory support in the intensive care unit (ICU).

## Methods

### Design

ESSENTIAL was a randomized, double-blind, placebo-controlled trial, in which one dose of nangibotide was tested versus placebo. The study was conducted in 14 academic/university hospitals in France and Belgium. The study was overseen by an independent Data Monitoring Committee (DMC).

The study was divided into two parts running sequentially without unblinding. Part 1 evaluated safety and tolerability and included 60 patients, randomized in a 2:1 ratio of nangibotide to placebo. Part 2 had an initially planned sample size of 370 patients (including those recruited in part 2), randomized in a 1:1 ratio of nangibotide to placebo. The sample size required to evaluate the primary endpoint was based upon the demonstration of a comparable effect size to that observed in early phase trials of immunomodulatory therapies in severe COVID-19.^21^ A computer generated randomization scheme was developed by an independent statistician who was not part of the study team. Randomization assignment was implemented using an Interactive Response Technology (IRT) with randomization stratified by site. Patients were assigned in a blinded fashion to one of the treatment groups.

A complete list of investigators and contributors to the study is provided in the electronic supplementary material (ESM 1).

### Ethics

All patients or their legally authorised representatives provided written informed consent or, in relevant countries, an independent physician, confirmed patient eligibility for enrolment in the trial.

The trial procedures and the informed consent form (ICF) process were approved by the respective independent ethics committee (IEC) following international standards and national requirements of each participating country. The study has been approved by the responsible ethics committees/institutional review boards in all study countries. The study was registered in the EU Clinical Trials Register (EudraCT Number: 2020-001504-42) ClinicalTrials.gov: NCT04429334.

### Participants

Patients (aged 18–75 years inclusive) were eligible for enrolment within 48 h of the initiation of non-invasive, including high flow oxygen, or invasive respiratory support in an ICU setting primarily for the treatment of SARS-CoV-2 induced respiratory failure if they met all inclusion and no exclusion criteria. Main inclusion criteria were the presence of documented SARS-CoV-2 infection in the preceding seven days, non-invasive (a baseline clinical status score of 5) or invasive (a clinical status of 6) respiratory support, a PaO_2_:FiO_2_ ratio of <200 mmHg (<26.7 kPa) and a FiO2 ≥0.6 before the initiation of study drug; a degree of hypoxia consistent with moderate to severe Acute Respiratory Distress Syndrome (ARDS). The PaO_2_:FiO_2_ ratio was estimated using the inspired oxygen fraction set by the supportive device that was employed i.e. the ventilator, non invasive ventilation (NIV) or high flow nasal oxygen (HFNO) at the time of screening. The partial pressure of oxygen was measured using arterial blood gas analysis.

The main exclusion criteria related to the presence of severe life limiting comorbidity, pregnancy and chronic immunosuppression. A full list of inclusion and exclusion criteria is included in the ESM 2.

### Intervention

Patients received a continuous infusion of nangibotide at 1.0 mg/kg/h or a matched placebo. Study drug was issued as a lyophilized white powder in 50 ml glass vials containing either nangibotide or placebo. The powder was solubilized with water for injection at the site and infused at the prescribed rate based upon actual body weight as a continuous infusion via a central vein. Treatment was initiated as early as possible, but no later than 48 h after the initiation of respiratory support (NIV, HFNO or invasive ventilation). Patients were treated with study drug for a total duration of 5 days (120 ± 2 h) or until ICU discharge, whichever was sooner. The rationale for administering nangibotide was that this is the period of sustained TREM-1 activation observed in both preclinical models and observational human data sets.

The treatment was administered in addition to standard of care. Follow-up visits were performed daily until day 14. The end of study visit was at day 28 and a further follow up visit assessing survival was conducted at day 60.

### Outcome measures

The primary endpoint in part 1 of the trial was safety described by the incidence of adverse events and mortality until day 28. The primary endpoint of part 2 (assessed in all patients included in both parts of the study) was the improvement in clinical status (7-point Ordinal Scale) assessed at day 28, with a score of 1 reflecting a patient at home and independent, and a score of 7 being allocated to a non survivor. The categorizations of the score are provided in Supplementary Table S1.

Secondary outcomes included all-cause mortality at day 28 and day 60 (day 60 mortality data for patients included in part 1 of the trial was collected after completion of the study, detailed in ESM 2), duration and nature of supported ventilation and hospital free survival (the proportion of patients alive and not hospitalized on day 28). Exploratory safety outcomes included the incidence of thromboembolic events and the rate of secondary infection. At the day 60 visit, subjects were invited to complete (in person or by telephone) the EQ5D-3L assessment. In brief, this involved 5 questions regarding functional status across a range of categories (mobility, self care, usual activities, pain/discomfort, anxiety/depression) graded on a three point scale including severe limitation, some limitation or no limitation as possible responses.

### Interim analyses

Two pre-planned interim analyses were conducted by an independent unblinded data monitoring committee (DMC) after 60 patients and after 130 patients had been randomized. The first interim evaluated safety and the second interim analysis assessed both safety and futility. The DMC charter and statistical analysis plan (SAP) for the futility analysis are provided in the ESM 3 and 4. The DMC did not raise concerns during either interim analysis or the study proceeded without modification.

### Data analysis and statistics

Summaries of the methods employed in the analysis of safety and efficacy are provided in the statistical analysis plans (DMC SAP, ESSENTIAL SAP) (ESM 4, 5).

Efficacy, safety, and tolerability endpoints were assessed in all patients who were randomized to a treatment group and received at least one dose of study drug (modified intent-to-treat (ITT) analysis). The per-protocol (PP) analysis included all patients who, in addition, received the trial medication according to the protocol with minor deviations only and satisfied all major entry criteria. Demographic and medical background data, safety variables and secondary endpoints were analysed using descriptive statistics. Continuous data were analysed based on the mean (SD) or median (IQR) depending upon the distribution of the data. Categorial variables are summarized using counts and frequencies for contingency tables. All-cause mortality was evaluated using data collected from adverse event, study visit and mortality sections of the clinical report form.

In order to prevent any potential impact of imbalance between the study groups in terms of baseline respiratory support, *a priori,* the planned evaluation of improvement in clinical status was compared between groups with a Cochran-Mantel Haenszel test stratified only by clinical status at baseline (5 or 6) using modified ridit scores. The Wald test p-value for the difference in raw mean scores is provided. Patients with a missing clinical status at day 28 and who died prior to or on day 28 were assigned the category of 7 at day 28. Patients with a missing clinical status at day 28 and without documented death occurring prior to or on day 28 were assigned the last available clinical status.

The *a priori* planned analysis of the secondary endpoint all-cause mortality at day 28 was conducted using a logistic regression model adjusting for the same covariates used for the primary endpoint analysis (baseline clinical status only). The treatment effect comparing nangibotide vs placebo was estimated in terms of an odds ratio associated with a 95% CI and p-value. The PH Cox regression model included treatment and stratification factors. Hence, the hazard ratio for treatment is an adjusted hazard ratio. Hazard Proportionality was assessed by visual inspection of Schoenfeld residuals and tested with the Score Test based on weighted Schoenfeld residuals.

The relationship between the degree of TREM-1 activation (defined by sTREM-1 level at baseline) and treatment effect was explored with regards to selected clinically relevant outcomes. The treatment effect of each dose of nangibotide vs placebo was evaluated for potential in the overall population and in the group of patients with a sTREM-1 level above the median value at baseline (high sTREM-1 group).

Due to the exploratory nature of this trial that was stopped earlier than planned, an adjusted alpha nominal level was employed for the test of the primary endpoint to take into account the early stop and control the type 1 error at its usual 1-sided level of 0.025. A conservative alpha spending function was used blind to control the type 1 error: the nominal 1-sided alpha level considered is 0.003303, derived from the Lan DeMets Obrien-Fleming spending function at 220 patients. Preparation of the mITT and PP analyses was conducted based on the pre-planned SAP by the biostatistics team of the contract research organization. The exploratory evaluation of the relationship between the degree of TREM-1 activation and treatment response was conducted by the study statistician. No adjustment for multiple endpoints was performed as other endpoints in this trial are considered as exploratory. Statistical analyses were performed using SAS version 9.3, and R version 3.4.3.

### Role of the sponsor

The sponsor contributed to the design of the trial, design of the statistical analysis plan, post hoc exploratory analyses, preparation of the manuscript and the decision to submit. The sponsor played no role in patient screening, recruitment, collection of data, analysis or interpretation of the main outcomes.

## Results

### Patients and study treatment

The first patient was enrolled on 23rd September 2020 and the last patient on 29th April 2022. There was a pause in recruitment between the end of January 2021 at the end of part 1 and the start of part 2 in August 2021 to facilitate availability of additional study drug and matched placebo. Availability of study drug limited the maximum recruitment rate during part 2 of the trial.

**Fig. 1:**
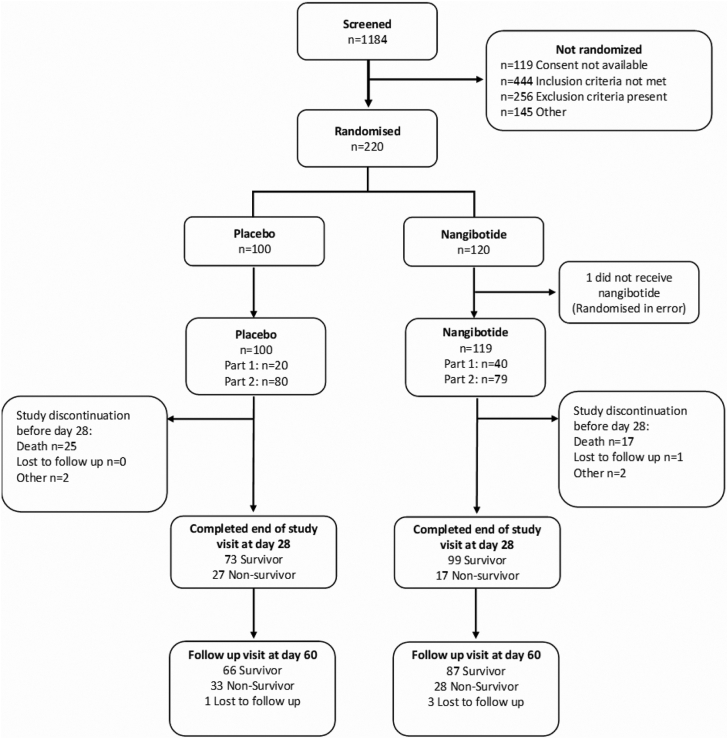
Patient recruitment flowchart.

1184 patients were screened and the study was stopped after 220 patients were randomized. Of these patients, one was identified as being ineligible for inclusion between randomization and initiation of treatment and did not receive study drug, therefore 219 patients were included in the mITT set. Analysis of the safety and efficacy of nangibotide in the mITT set was therefore based upon 119 patients treated with nangibotide and 100 patients treated with placebo (Fig. 1). The proportion of patients with a clinical status of 5 at baseline was 69/100 (69.0%) in the placebo and 80/119 (67.2%) in the nangibotide treated patients. The remainder of the patients, 31 (31.0%) placebo and 39 (32.8%) nangibotide had a baseline clinical status score of 6 (invasive mechanical ventilation). No patients were undergoing extracorporeal membrane oxygenation (ECMO) at randomization. During the course of the study, 3 (3.0%) patients in the placebo group and 5 patients (4.2%) underwent ECMO at any stage between baseline and day 28.

Low recruitment primarily due to reduced numbers of patients admitted to ICU with COVID-19 coupled with the changes in the proportion of patients developing severe disease that were considered eligible led to the early termination of the study with an adequate sample size available to design a phase 3 trial.

Baseline characteristics for the overall population and the group of patients with high sTREM-1 are shown in Table 1. The population as a whole displayed typical features of patients admitted to ICU with COVID-19. Whilst there were no significant imbalances between the study populations, the proportion of patients that received dexamethasone was 70% in the nangibotide treated population and 65% in the placebo group. The mean (SD) time from onset of support to inclusion was 17.4 (12.1) hours in the placebo group and 22.3 (14.5) hours in the nangibotide treated population.

**Table 1:** Baseline characteristics of patients included in the ESSENTIAL study.

	Placebo (n = 100)	Nangibotide (n = 119)
**Demographics**		
Age (Years)	61.3 (10.9)	62.4 (10.3)
Sex female N (%)	16 (16.0)	28 (23.5)
Body mass index (kg/m^2^)	28.8 (4.0)	28.9 (4.7)
**Ethnicity**		
White or Caucasian n (%)	96 (96.0)	111 (93.3)
Black or African American n (%)	2 (2.0)	4 (3.4)
Asian n (%)	0 (0.0)	2 (1.7)
Hispanic, Latino or of Spanish origin n (%)	1 (1.0)	0 (0.0)
Native Hawaiian or other pacific islander n (%)	0 (0.0)	1 (0.8)
Other n (%)	1 (1.0)	1 (0.8)
**Respiratory Support at baseline**		
Invasive ventilation, n (%)	31 (31.0)	39 (32.8)
Non-invasive ventilation, n (%)	1 (1.0)	4 (3.4)
High flow, n (%)	68 (66.0)	81 (68.1)
Period: Initiation of respiratory support to study drug start (hrs)	17.4 (12.1)	22.3 (14.5)
**Organ Support at baseline**		
Vasopressor n (%)	16 (16)	18 (15)
Renal replacement therapy n (%)	0 (0)	0 (0)
**sTREM-1 AT BASELINE (pg/ml)**		
Mean (SD)	290.8 (216.2)	273.7 (166.6)
Median	255.0	231.0
Min-max	87–1810	91–1140
**ARDS severity**		
PaO_2_:FiO_2_ ratio^a^	88.0 (71.4–111.2)	92.2 (77.0–118.9)
PaO_2_:FiO_2_ ratio 100–200 n (%)	27 (27)	43 (36)
PaO_2_:FiO_2_ ratio <100 n (%)	60 (60)	56 (47)
**SOFA at Baseline**		
Mean (SD)	5.3 (2.11)	4.8 (2.28)
**IMP Exposure (hrs)**		
Median	120.0	120.0
Min-max	23.7–132.0	28.7–132.2
**Co-administered Therapies**		
Dexamethasone n (%)	65 (65)	84 (70.6)
Tocilizumab n (%)	17 (17)	17 (14.2)
Vaccinated against SARS-CoV-2 n (%)	20 (20)	21 (17.7)

Data presented as mean (SD) or median (IQR) unless specified. Patients reported as having received at least one dose of a licensed vaccine for COVID-19 were considered vaccinated. A number of patients were reported as receiving intermittent high flow nasal oxygen or non invasive ventilation, and both were recorded as baseline support. Data are reported as recorded by the sites.

a All patients had PaO_2_:FiO_2_ evaluated at screening, however 13 (13%) of placebo and 21 (18%) of nangibotide treated patients were missing PaO_2_:FiO_2_ at baseline and are not reported in this table of features at the time of initiation of study drug.

### Safety of nangibotide

The safety of nangibotide was the primary outcome of part one of the study and was assessed throughout the trial. Results of the analysis of safety and tolerability are summarized in Table 2. The incidence of treatment emergent adverse events (TEAEs) and serious TEAEs was similar in the placebo and treatment groups. 194/219 (88.6%) of patients experienced at least one TEAE. Classification of TEAE by system organ class is provided in the supplementary files for the overall population and patients in the high sTREM-1 group (ESM 6). Four patients experienced a serious TEAE considered possibly related to study drug with three of those patients in the placebo group and one in the nangibotide group. None of the serious TEAEs considered possibly related to study drug led to the premature cessation of study drug. Exploratory safety outcomes related to common complication of ICU admission for COVID-19 revealed no increase in the rate of thromboembolic events with 16/100 (16.0%) of placebo patients diagnosed with thrombus in at least one site compared to 19/119 (16.0%) in the nangibotide treated patients. 53/119 (44.5%) of nangibotide treated patients developed at least one secondary respiratory infection compared to 57/100 (57.0%) of those that received placebo, ventilator associated pneumonia was reported in 54/100 (54%) of the placebo patients and 48/119 (40.3%) of nangibotide treated patients.

**Table 2:** Cumulative incidence (n, %) of adverse events in patients treated with placebo and nangibotide in the ESSENTIAL trial between initiation of study drug and study day 28.

	Placebo (n = 100)	Nangibotide (n = 119)
Total AE n	587	659
Patients with at least 1 AE n (%)	90 (90.0)	104 (87.4)
Total AE related to study drug n	5	4
Patients with at least 1 AE related to study drug n (%)	4 (4.0)	4 (3.4)
Total AE leading to death n	27	19
Total SAE n	60	63
Patients with at least 1 SAE n (%)	43 (43.0)	33 (27.7)
Total SAE related to study drug n	3	1
Patients with at least 1 SAE related to study drug n (%)	3 (3.0)	1 (0.8)

### Efficacy of nangibotide

#### Primary outcome

**Fig. 2:**
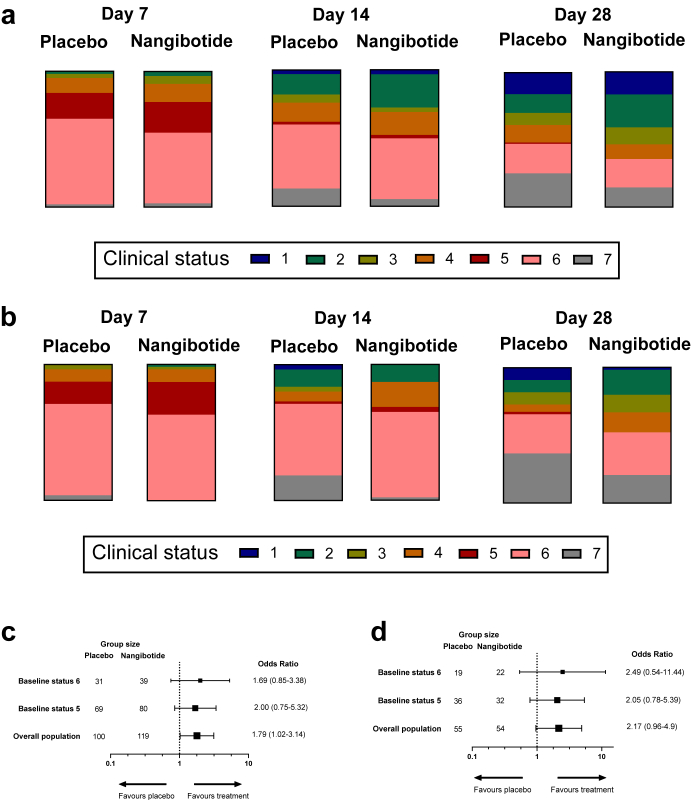
The impact of nangibotide administration on clinical status at days 7, 14 and 28 and all-cause mortality. Clinical status was evaluated using a seven point ordinal scale to describe the condition of patients at each time point with a score of 1 describing patients who were Not hospitalized, with no limitations of activities at the time point assessed, 2: Not hospitalized, some limitations of activities, 3: Hospitalized, not requiring supplemental oxygen, 4: Hospitalized, requiring supplemental oxygen, 5:Hospitalized, on non-invasive ventilation or high flow oxygen devices, 6: Hospitalized, on invasive mechanical ventilation or ECMO and 7: those who were not alive at the time of the visit. The distribution of clinical status was assessed at multiple time points, and the distribution described at the day 7, 14 and 28 visits in **a**; the overall study population and **b**: the group of patients with a sTREM-1 level above the median value at baseline. The impact of nangibotide therapy on the odds ratio for improvement in clinical status (categorised as improved or not improved at day 28). The odds ratio for improvement is presented in patients with a baseline clinical status score of 6, a baseline status score of 5 and in the overall population (Adjusted for baseline clinical status) in **c**: the overall and **d**: the high sTREM-1 population.

The primary efficacy outcome assessed in all patients in the trial was the odds ratio for improvement in clinical status at day 28 from baseline. Table 3 describes the distribution of clinical status on day 28 for the nangibotide and placebo patients in the overall population and in the group of patients with a high sTREM-1 at baseline. The evolution of clinical status at days 7, 14 and 28 is described for the overall population in Fig. 2a and the high sTREM-1 group in Fig. 2b. The Cochran-Mantel Haenszel test stratified by clinical status at baseline (5 or 6) using modified ridit scores resulted in a p value of 0.04 in the overall population and p value of 0.128 in the high sTREM-1 group. The pattern of efficacy observed in the primary analysis was consistent in the subgroups of patients with a baseline score of 5 or 6 (Supplementary Tables S2 and S3).

**Table 3:** The distribution of clinical status at day 28 in patients included in the ESSENTIAL trial.

Value	Clinical status definition	Overall population		High sTREM-1	
		Placebo	Nangibotide	Placebo	Nangibotide
		n (%)	n (%)	n (%)	n (%)
1	Not hospitalized, no limitations of activities	16 (16.0%)	20 (16.8%)	5 (9.1%)	1 (1.9%)
2	Not hospitalized, limitations of activities	14 (14.0%)	29 (24.4%)	5 (9.1%)	10 (18.5%)
3	Hospitalized, not requiring supplemental oxygen	9 (9.0%)	15 (12.6%)	5 (9.1%)	7 (13.0%)
4	Hospitalized, requiring supplemental oxygen	13 (13.0%)	13 (10.9%)	3 (5.5%)	8 (14.8%)
5	Hospitalized, on non-invasive ventilation or high flow oxygen devices	1 (1.0%)	0 (0.0%)	1 (1.8%)	0 (0.0%)
6	Hospitalized, on invasive mechanical ventilation or ECMO	22 (22.0%)	25 (21.0%)	16 (29.1%)	17 (31.5%)
7	Death	25 (25.0%)	17 (14.3%)	20 (36.4%)	11 (20.4%)

The number (%) of patients in each of the seven categories is described for patients that received placebo and nangibotide in the overall population and in the subgroup of patients with high sTREM-1 defined by a sTREM-1 above the median value (239 pg/ml) at baseline.

A planned sensitivity analysis categorizing the patients as improved or not improved from baseline at day 28 was conducted. All patients started with a baseline clinical status of 5 or 6 and any reduction in that score from baseline at the time of the day 28 visit was considered improvement. This analysis was also adjusted for baseline clinical status (5 or 6). Fifty-two (52.0%) of patients had improved in the placebo group compared to 77 (64.7%) of the nangibotide treated population, an odds ratio (95% CI) of 1.79 (1.02–3.14), p = 0.043 (Fig. 2c). In the high sTREM-1 population, 18 (32.7%) of placebo patients had improved by day 28 compared to 26 (48.1%) of treated patients, with an odds ratio (95% CI) of 2.17 (0.96–4.90), p = 0.063 (Fig. 2d). Odds ratio for improvement in clinical status in patients with a baseline status score of 5 was 2.00 (0.75–5.32) in the overall population and 2.05 (0.78–5.39) in the high sTREM-1 group. In patients with a baseline status score of 6, the odds ratio for improvement was 1.69 (0.85–3.38) and 2.49 (0.54–11.44) in the overall and high sTREM-1 groups respectively.

#### Secondary outcomes

**Fig. 3:**
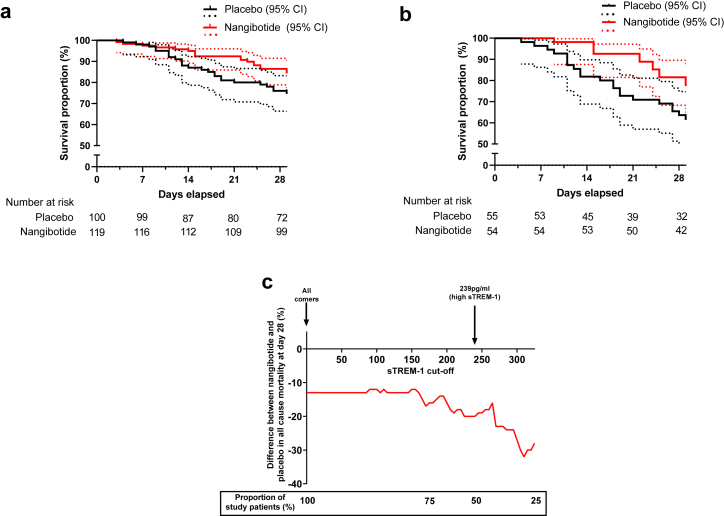
Impact of nangibotide on survival at day 28 and relationship between sTREM-1 at baseline and treatment response. Patients with death occurring between start of study drug infusion and the day 28 visit which occurred at 28 days (with a 2 day visit window) are considered dead at day 28. One patient (nangibotide group) was lost to follow up following discharge from hospital alive on day 11 and was censored at the date last known to be alive. Time to event analysis of all-cause mortality in the **a**: overall and **b**: high sTREM-1 populations up to day 28 with 95% confidence intervals and the number of patients at risk at each time point reported with a y axis range of 50–100% survival percentage **c**: The relationship between increasing baseline sTREM-1 level and treatment efficacy of nangibotide. The difference in mortality between placebo and nangibotide treated patients is plotted (red line) for increasing cut-off values of sTREM-1. The proportion of patients included in the analyses at selected cut offs is provided in the boxed section.

The key secondary outcome was all cause mortality at day 28. In the overall population, 28 (28.0%) of placebo treated patients were not alive at the day 28 visit compared to 19 (16.0%) of nangibotide treated patients, with an absolute reduction (95% CI) in mortality, adjusted for baseline clinical status of 12.1% (23.05–1.18), p = 0.030. In the high sTREM-1 population, 23 (41.8%) of patients in the placebo group and 12 (22.2%) of patients in the nangibotide group were not alive at day 28 with an adjusted reduction in mortality of 19.88% (36.98–2.78), p = 0.023. Time to event analysis of death before day 28 demonstrated a hazard ratio (95% CI) for death of 0.52 (0.29–0.93) with a p = 0.027 (Cox Proportional Hazards) in the overall population (Fig. 3a) and a hazard ratio (95% CI) of 0.44 (0.22–0.89), p = 0.023 in the high sTREM-1 group (Fig. 3b). Of note, in patients with a sTREM-1 level below the median value (<240 pg/ml), the placebo group mortality was 5/44 (11.4%) and 6/64 (9.4%) in the nangibotide treated population with an adjusted difference of 2.14 (−9.5% to 13.7%).

An exploratory analysis of the impact of therapy in patients treated with dexamethasone who did not receive tocilizumab (n = 119) identified 15/51 (29.4%) and 14/68 (20.6%) all-cause mortality at day 28, an adjusted difference of −8.82% (−24.61 to 6.96%). In the subgroup of these patients with high sTREM-1, mortality was 12/27 (44.4%) vs 9/31 (29.0%), an adjusted difference of −14.92% (−39.7 to 9.85%) (Supplementary Fig. S1).

At day 60, survival was evaluated as a binary endpoint in patients included in both parts of the study. The same pattern of benefit following nangibotide therapy was observed. Non-survivors made up 33 (33.0%) of placebo treated patients and 28 (23.5%) of those that received study drug in the overall population at day 60, with an adjusted difference in mortality of −9.50% (−21.43 to 2.43%) p = 0.118. In the high sTREM-1 subgroup, the mortality at the day 60 visit was 27/55 (49.1%) in the placebo group and 19/54 (35.2%) in the nangibotide treated population, with an adjusted difference in mortality of −14.86% (−33.05 to 3.33%) p = 0.109.

The proportion of patients alive and free of respiratory support at day 28 (defined as the liberation from any of: invasive ventilation, NIV or HFNO at the day 28 visit) was 73/100 (61.3%) compared to 51/119 (51.0%) in the overall population and 24/54 (44.4%) vs 18/55 (32.7%) in the high sTREM-1 patients, in the nangibotide treated and placebo groups respectively. Descriptive analysis was undertaken in part 2 of the study of the EQ5D-3L functional status evaluation at day 60. The data revealed that nangibotide treatment did not result in an increased proportion of patients reporting severe limitation of function compared to no or some dysfunction following nangibotide therapy in either the overall or the high sTREM-1 groups (Supplementary Tables S4 and S5 respectively).

### Evaluation of the relationship between TREM-1 activation and treatment response

The planned exploration of the relationship between sTREM-1 at baseline and subsequent outcome was undertaken using day 28 mortality. Treatment effect was described for patients with a sTREM-1 level categorized by each percentile from 0 to 75. Increasing levels of sTREM-1 prior to the initiation of therapy was associated with a potential increase in effect of nangibotide (Fig. 3c).

### Per protocol analysis

Eleven patients were excluded from the PP analysis, 10 due to deviation from inclusion or exclusion criteria and one due to deviation from protocolised drug administration, details are listed in Supplementary Table S6. Patient characteristics for the PP population were similar to those of the mITT set. No substantial differences were detected in the primary outcome in either the overall or the high sTREM-1 patients.

### Interpretation

The ESSENTIAL study is the first to evaluate the safety and efficacy of a TREM-1 modulatory strategy with nangibotide in critically ill patients with COVID-19. Treatment led to improved clinical status at day 28 and an absolute reduction in mortality of 12% in the overall population and 19.9% on the group of patients that met the *a priori* definition of elevated sTREM-1 at baseline, a pattern of benefit that persisted at 60 days after randomization. Secondary and exploratory analyses suggested that nangibotide treated patients displayed a pattern of fewer adverse events and secondary infections compared to placebo. Notably, patients in the placebo group with a sTREM-1 level below the median value had a lower risk of death compared to those with a high sTREM-1 level, consistent with the prognostic power of sTREM-1 in this setting.

The use of a TREM-1 modulatory strategy in COVID-19 was based upon existing preclinical and clinical proof of concept. TREM-1 expression is upregulated following stimulation of human monocytes, endothelial cells, and neutrophils with various TLR ligands, including those implicated in antiviral response (mainly TLR 3, 7, 8, 9). Co-activation of TREM-1 with these stimuli results in an increased production of cytokines.^22^, ^23^, ^24^, ^25^, ^26^ Translational evidence implicating TREM-1 in the pathophysiology of severe disease has been shown with a number of single strand RNA viruses.^27^, ^28^, ^29^ An extensive preclinical data set supporting the approach in general and specifically with nangibotide has been developed in a number of models of inflammation and infection.^3^^,^^17^^,^^30^ In addition, observational studies in patients admitted to hospital with COVID-19 have shown an association between the degree of TREM-1 activation, the development of severe disease and ultimate outcome.^6^^,^^8^

This study explored the hypothesis that the efficacy of nangibotide was associated with the degree of TREM-1 activation (defined by the level of sTREM-1 in the circulation), an observation that has been made in a phase 2a septic shock trial^31^ and was simultaneously evaluated in the phase 2b ASTONISH study.^20^ Consistent with the localized endothelial and immune dysregulation that typifies severe COVID-19 pneumonitis, the absolute value of sTREM-1 measured in the circulation in the ESSENTIAL population is similar to that seen in observational studies^8^ and lower (median sTREM-1 of 239 pg/ml) than that seen in septic shock, where systemic endothelial and immune dysregulation dominates and higher average levels are observed.^31^ This study confirms the prognostic potential of the biomarker in severe COVID-19 with mortality in the placebo group substantially greater in the high sTREM-1 patients, a pattern also observed in previous observational trials.^6^^,^^8^ Furthermore, whilst the available sample size limits statistical inference, this study also supports the potential for sTREM-1 to act as tool to identify potential treatment populations for nangibotide therapy, with a higher modifiable mortality and relative effect size in the group of patients with an elevated sTREM-1 at baseline. This observation would facilitate the design of a precision medicine phase 3 trial in acute respiratory distress syndrome (ARDS).

This study allowed the use of all standard of care therapies for the treatment of COVID-19 before and after inclusion in the trial. Immunomodulatory medications considered the standard of care for this population evolved during the study period, with 68% of patients in the trial receiving dexamethasone, a pattern that reflects the recommendation for the increased use of dexamethasone following the results of the RECOVERY trial.^5^ 15.5% of patients received tocilizumab therapy which, following conflicting results in double blind randomized trials^32^ was ultimately approved for use following the results of large platform trials,^4^^,^^33^ although uptake has been variable. Exploratory analysis of the data suggests that treatment with dexamethasone does not reduce the efficacy of nangibotide in this setting.

This trial was terminated earlier than planned due to the substantial reduction in the number of eligible patients admitted to ICU in France and Belgium where the study was conducted, which has consequences in terms of statistical inference and the p value required to achieve formal statistical significance in this trial was not achieved for the primary outcome. However as an exploratory trial, the sample size of 220 patients is sufficient to detect signals to efficacy in this population.

In conclusion, this study presents results of an exploratory trial exploring the impact of nangibotide therapy in patients with life threatening COVID-19. A pattern of improved outcome was observed following nangibotide treatment, with exploratory evidence supporting a greater magnitude of effect in patients with higher levels of TREM-1 pathway activation. A future study will explore the further development of a precision medicine strategy for nangibotide therapy in life threatening infectious pneumonitis.

## Contribution

SL drafted the first version of the manuscript. BF, PFL, ML, JMG and JJG revised the first draft. SL, JJG, MD, SG, VC, MSM, JMG, BF, PFL, ML and ALLP all contributed to the development and design of the protocol, data collection and analysis. PA, CD, JPM, JPQ, JL, EM, JCL, CV, TF, JH, SG and JB recruited ten or more patients to the trial and participated in data collection. SL and JJG verified the data. SL, JJG, JMG and BF had access to unblinded raw data only after database lock. All authors reviewed the manuscript and approved the final version.

## Data sharing statement

Data will be shared with investigators whose proposed approach is methodologically sound and is designed to achieve the aims of the proposed research. Proposals should be submitted to the corresponding author. To gain access, data requestors will need to sign a data access agreement.

## Declaration of interests

SL, MSM, JJG, MD, VC and ALLP are employees of Inotrem SA. BF, ML and PFL are members of the steering committee. BF reports personal fees for consulting from Aridis, Enlivex, AM-Pharma, and Inotrem outside the submitted work. JJG, MD, MSM, SG, SL and ALLP hold shares in Inotrem. MD, JJG, SG and MSM are designated as an inventor on patent(s) related to Inotrem. PFL reports consulting fees from Adrenomed and Inotrem outside the submitted work. SG and SL reports consulting fees from Inotrem outside the submitted work. SL is director and holds shares in Critical Pressure Ltd, and is designated as an inventor on a patent related to Critical Pressure Ltd, outside the submitted work. JJG reports consulting travelling support from Inotrem outside the submitted work. JH reports honoraria for lectures from Diagnostica Stago, Pfizer PFE France, Sanofi Aventis France, Inotrem, MSD, Octapharma and Shionogi outside the submitted wok. PA's institution received payment for the recruitment of patients in the study. JMG, CD, JPM, JPQ, JL, EM, JCL, CV, TF and JB have no conflict of interests to report.

## Figures and Tables

**Figure:**
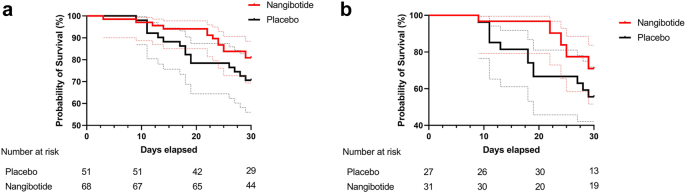
Supplementary Fig. S1

## Appendix A Supplementary data

ESM 6 TEAE by SOC

ESM3 MOT-C-204_DMC charter-v3.0_FE

ESM4 INOMOTC204 DMC SAP

ESM5 INOMOTC204 Final SAP

ESSENTIAL ESM1

ESSENTIAL-eClinicalMedicine-ESM2-R1-CLEAN
