# Supplementary material for: Evaluation of the efficacy and safety of TREM-1 inhibition with nangibotide in patients with COVID-19 receiving respiratory support: the ESSENTIAL randomised, double-blind trial
Source: eClinicalMedicine. 2023 May 31;60:102013. doi: 10.1016/j.eclinm.2023.102013 (PMC10231876; doi:10.1016/j.eclinm.2023.102013)
Supplement: ESM 6 TEAE by SOC [file mmc1.pdf]

Treatment-Emergent Adverse Events with Outcome of Death by System Organ Class and Preferred Term
Safety Set

System Organ Class<br>Preferred Term	Placebo<br>(N=100)<br>n (%) [E]	Nangibotide<br>1.0 mg/kg/h<br>(N=119)<br>n (%) [E]	Total<br>(N=219)<br>n (%) [E]
TOTAL NUMBER OF TREATMENT-EMERGENT ADVERSE EVENTS WITH OUTCOME OF DEATH	27	19	46
NUMBER OF PATIENTS WITH AT LEAST ONE TREATMENT-EMERGENT ADVERSE EVENT WITH OUTCOME OF DEATH	27 (27.0)	19 (16.0)	46 (21.0)
RESPIRATORY, THORACIC AND MEDIASTINAL DISORDERS	13 (13.0) [13]	9 (7.6) [9]	22 (10.0) [22]
ACUTE RESPIRATORY DISTRESS SYNDROME	1 (1.0) [1]	1 (0.8) [1]	2 (0.9) [2]
ACUTE RESPIRATORY FAILURE	1 (1.0) [1]	0	1 (0.5) [1]
HAEMOTHORAX	0	1 (0.8) [1]	1 (0.5) [1]
HYPOXIA	4 (4.0) [4]	3 (2.5) [3]	7 (3.2) [7]
PULMONARY EMBOLISM	0	1 (0.8) [1]	1 (0.5) [1]
RESPIRATORY DISTRESS	0	2 (1.7) [2]	2 (0.9) [2]
RESPIRATORY FAILURE	7 (7.0) [7]	1 (0.8) [1]	8 (3.7) [8]
INFECTIONS AND INFESTATIONS	6 (6.0) [6]	6 (5.0) [6]	12 (5.5) [12]
COVID-19	1 (1.0) [1]	1 (0.8) [1]	2 (0.9) [2]
SEPTIC SHOCK	5 (5.0) [5]	5 (4.2) [5]	10 (4.6) [10]
GENERAL DISORDERS AND ADMINISTRATION SITE CONDITIONS	6 (6.0) [6]	0	6 (2.7) [6]
MULTIPLE ORGAN DYSFUNCTION SYNDROME	6 (6.0) [6]	0	6 (2.7) [6]
NERVOUS SYSTEM DISORDERS	1 (1.0) [1]	2 (1.7) [2]	3 (1.4) [3]
BRAIN INJURY	0	1 (0.8) [1]	1 (0.5) [1]
CEREBRAL HAEMORRHAGE	0	1 (0.8) [1]	1 (0.5) [1]
HAEMORRHAGIC STROKE	1 (1.0) [1]	0	1 (0.5) [1]

At each level of patient summarization, a patient is counted once if the patient reported one or more events. [E] represents the number of events at each level of summarization. Adverse Events were coded using MedDRA, Version 23.0.
Source Data: Listing 11

Treatment-Emergent Adverse Events with Outcome of Death by System Organ Class and Preferred Term
Safety Set

System Organ Class<br>Preferred Term	Placebo<br>(N=100)<br>n (%) [E]	Nangibotide<br>1.0 mg/kg/h<br>(N=119)<br>n (%) [E]	Total<br>(N=219)<br>n (%) [E]
VASCULAR DISORDERS	1 (1.0) [1]	2 (1.7) [2]	3 (1.4) [3]
PERIPHERAL ISCHAEMIA	0	1 (0.8) [1]	1 (0.5) [1]
SHOCK	1 (1.0) [1]	1 (0.8) [1]	2 (0.9) [2]

At each level of patient summarization, a patient is counted once if the patient reported one or more events. [E] represents the number of events at each level of summarization. Adverse Events were coded using MedDRA, Version 23.0.
Source Data: Listing 11

Treatment-Emergent Adverse Events with Outcome of Death by System Organ Class and Preferred Term
Safety Set Patients with Baseline sTREM-1 Level Above or Equal to the Median

System Organ Class<br>Preferred Term	Placebo<br>(N=55)<br>n (%) [E]	Nangibotide<br>1.0 mg/kg/h<br>(N=54)<br>n (%) [E]	Total<br>(N=109)<br>n (%) [E]
TOTAL NUMBER OF TREATMENT-EMERGENT ADVERSE EVENTS WITH OUTCOME OF DEATH	22	13	35
NUMBER OF PATIENTS WITH AT LEAST ONE TREATMENT-EMERGENT ADVERSE EVENT WITH OUTCOME OF DEATH	22 (40.0)	13 (24.1)	35 (32.1)
RESPIRATORY, THORACIC AND MEDIASTINAL DISORDERS	11 (20.0) [11]	7 (13.0) [7]	18 (16.5) [18]
ACUTE RESPIRATORY DISTRESS SYNDROME	1 (1.8) [1]	1 (1.9) [1]	2 (1.8) [2]
ACUTE RESPIRATORY FAILURE	1 (1.8) [1]	0	1 (0.9) [1]
HYPOXIA	3 (5.5) [3]	3 (5.6) [3]	6 (5.5) [6]
PULMONARY EMBOLISM	0	1 (1.9) [1]	1 (0.9) [1]
RESPIRATORY DISTRESS	0	2 (3.7) [2]	2 (1.8) [2]
RESPIRATORY FAILURE	6 (10.9) [6]	0	6 (5.5) [6]
INFECTIONS AND INFESTATIONS	6 (10.9) [6]	3 (5.6) [3]	9 (8.3) [9]
COVID-19	1 (1.8) [1]	1 (1.9) [1]	2 (1.8) [2]
SEPTIC SHOCK	5 (9.1) [5]	2 (3.7) [2]	7 (6.4) [7]
GENERAL DISORDERS AND ADMINISTRATION SITE CONDITIONS	4 (7.3) [4]	0	4 (3.7) [4]
MULTIPLE ORGAN DYSFUNCTION SYNDROME	4 (7.3) [4]	0	4 (3.7) [4]
NERVOUS SYSTEM DISORDERS	1 (1.8) [1]	2 (3.7) [2]	3 (2.8) [3]
BRAIN INJURY	0	1 (1.9) [1]	1 (0.9) [1]
CEREBRAL HAEMORRHAGE	0	1 (1.9) [1]	1 (0.9) [1]
HAEMORRHAGIC STROKE	1 (1.8) [1]	0	1 (0.9) [1]

At each level of patient summarization, a patient is counted once if the patient reported one or more events. [E] represents the number of events at each level of summarization. Adverse Events were coded using MedDRA, Version 23.0.
Source Data: Listing 11

Treatment-Emergent Adverse Events with Outcome of Death by System Organ Class and Preferred Term
Safety Set Patients with Baseline sTREM-1 Level Above or Equal to the Median

System Organ Class<br>Preferred Term	Placebo<br>(N=55)<br>n (%) [E]	Nangibotide<br>1.0 mg/kg/h<br>(N=54)<br>n (%) [E]	Total<br>(N=109)<br>n (%) [E]
VASCULAR DISORDERS	0	1 (1.9) [1]	1 (0.9) [1]
PERIPHERAL ISCHAEMIA	0	1 (1.9) [1]	1 (0.9) [1]

At each level of patient summarization, a patient is counted once if the patient reported one or more events. [E] represents the number of events at each level of summarization. Adverse Events were coded using MedDRA, Version 23.0.
Source Data: Listing 11
