# Supplementary material for: Evaluation of the efficacy and safety of TREM-1 inhibition with nangibotide in patients with COVID-19 receiving respiratory support: the ESSENTIAL randomised, double-blind trial
Source: eClinicalMedicine. 2023 May 31;60:102013. doi: 10.1016/j.eclinm.2023.102013 (PMC10231876; doi:10.1016/j.eclinm.2023.102013)
Supplement: ESM3 MOT-C-204_DMC charter-v3.0_FE [file mmc2.pdf]

DocuSign Envelope ID: E826A649-51DD-4828-9171-DF850DA937EA

DATA MONITORING COMMITTEE (DMC) CHARTER

MOT-C-204

Efficacy and Safety Study Exploring Nangibotide Treatment in COVID-19 pAtients with ventiLatory support
A randomized, double-blind, placebo-controlled study with adaptive features

The ESSENTIAL Study

**Test Product:**	Nangibotide
**Sponsor:**	INOTREM S.A.<br>54 rue de Ponthieu<br>75008 Paris<br>France
**Sponsor's Medical Officer:**	Jean-Jacques Garaud, MD
**Sponsor Protocol Number:**	MOT-C-204
**EudraCT Number:**	2020-001504-42
**pIND**	149168
**Development Phase:**	II/III
**DMC Chair:**	Michel Wolff, MD
**Version:**	3.0
**Date:**	30 June 2021

Confidential
Information in this protocol is confidential and should not be disclosed, other than to those directly involved in the execution or the ethical/regulatory review of the study, without written authorisation from INOTREM SA or its affiliates.

DocuSign Envelope ID: E826A649-51DD-4828-9171-DF850DA937EA

1 TABLE OF CONTENTS

1 Table of Contents 2
2 History of Changes 3
3 Approval Signatures 4
4 Study Contact Information 5
5 Preamble 6
6 Members 6
7 Responsibilities 6
8 DMC Meetings 6
9 DMC Meeting Minutes 9
10 DMC Recommendations 9
11 Record Retention 10
12 Reporting of Study Progress and Important Events to DMC 10
13 Communication Policy 10
14 Acceptance of DMC Recommendations 10
Appendix 1: DMC Members and Contact Information 12
Appendix 2: Safety Data Listings for DMC Review 13
Appendix 3: DMC CHAIR RECOMMNEDATION FORM 14

DocuSign Envelope ID: E826A649-51DD-4828-9171-DF850DA937EA

2 HISTORY OF CHANGES

Change from v2.0 dated 1 March 2021 to version v3.0 dated 30 June 2021:

– Change in study title and addition of an acronym

Changes from v1.0 dated 15 June 2020 to version v2.0 dated 1 March 2021:

- Changes in the responsibility of the DMC: DMC will review safety and efficacy data from unblinded interim analyses for futility.
- Addition of review meetings for the futility analyses of Part 2 of the study.
- Addition of information on data required from the futility analyses.

DocuSign Envelope ID: E826A649-51DD-4828-9171-DF850DA937EA

3 APPROVAL SIGNATURES

This study will be performed in compliance with the final protocol or approved amendments, the current Helsinki Declaration, Good Clinical Practices (GCP) and applicable regulatory requirements.

juillet 9, 2021 | 12:06 PM CEST

DocuSigned by:
Jean-Jacques GARAUD
F94C059B3693400...

Sponsor's Officer	Date:	Signature:

Jean-Jacques GARAUD, MD,
INOTREM

juillet 9, 2021 | 12:15 PM CEST

DocuSigned by:
Michel Wolff
1D5D93802FB84BB...

DMC Chair	Date:	Signature:

Michel Wolff, MD
DMC Chair

DocuSign Envelope ID: E826A649-51DD-4828-9171-DF850DA937EA

4 STUDY CONTACT INFORMATION

Sponsor's Representative and Medical Officer	Jean-Jacques Garaud, MD<br>Chief Executive Officer<br>Email: jjg@inotrem.com<br>Tel: +33 6 30 62 86 51
Sponsor's Main Contact	Valérie Cuvier<br>Project Manager<br>Email: vc@inotrem.com<br>Tel: +33 6 80 61 52 05
Sponsor Site	INOTREM SA<br>54 rue de Ponthieu<br>75008 Paris, France
Coordinating Investigator	Professor Bruno François, MD<br>Réanimation Médicale<br>Hôpital Universitaire Dupuytren<br>Limoges, France<br>Email: bruno.francois@chru-limoges.fr<br>Tel: +33 5 55 05 62 54<br>Fax: +33 5 55 05 80 57
Data Monitoring Committee (Chair)	Michel Wolff, MD - DMC Chair<br>Service d'Anesthésie-Réanimation Neurochirurgicale<br>Pôle Neuro Sainte Anne<br>GHU Paris, Psychiatrie & Neurosciences<br>1, rue Cabanis<br>75674, Cedex 14, Paris, France<br>Tel : + 33 1 45 65 86 41<br>Mobile : + 33 6 87 33 28 45<br>Email: m.wolff@ghu-paris.fr and michel.wolff50@gmail.com
Unblinded Study Statistician	Nicolas Girard.<br>PPD France SAS<br>27-35 Rue Victor Hugo<br>94200 Ivry sur Seine - France<br>+33 1 82 88 66 43<br>nicolas.girard@ppdi.com
Pharmacovigilance Provider	STRAGEN SERVICES SAS<br>Le Quadrille 1<br>19 rue Jacqueline Auriol<br>69008 Lyon - France<br><br>Tel: +33 4 78 42 95 26<br>Fax: +33 4 78 42 55 71<br>Mobile + 33 6 22 35 68 20<br>pharmacovigilance@stragen.com

DocuSign Envelope ID: E826A649-51DD-4828-9171-DF850DA937EA

5 PREAMBLE

An independent data monitoring committee (DMC) will be appointed to monitor the safety of study MOT-C-204 *"Exploratory study of the safety, tolerability and efficacy of nangibotide in mechanically ventilated patients with COVID-19 and features of systemic inflammation. A randomized, double-blind, placebo-controlled study with adaptive features"*

6 MEMBERS

The DMC will comprise experts with experience in intensive care medicine and the conduct of clinical trials in this environment. It will consist of 4 members including 3 specialists in intensive care medicine and a methodologist (biostatistician) with experience in the conduct and the safety assessment of clinical trials. All four are voting members. The chair of the DMC will be appointed by the sponsor, INOTREM. Other DMC members will be mutually agreed between the sponsor and the DMC chair. The DMC members should be free of financial or scientific conflicts of interest and are responsible for advising the Sponsor of any changes in interests that would affect their objectivity. All DMC members will provide a signed CV and financial disclosure forms to the sponsor prior to the constitution of the DMC.

The current list of members and their contact information is provided in Appendix 1.

7 RESPONSIBILITIES

The DMC will be responsible for:

- Reviewing safety data from unblinded safety reports after each 20 randomized patients complete day 14 after randomization
- Reviewing SAEs on an ongoing basis
- Reviewing the general progress of the clinical study with regard to accrual/withdrawals or drop-out rates and clinical study conduct
- Reviewing safety and efficacy data from unblinded interim analyses for futility and at the end of each study part AND:
  - Making a recommendation for the study to stop in case of futility, continue unchanged or recommending an increase of sample size
  - Review the incidence of secondary infection, adverse events and death categorized by study drug and co-administered medication.

In addition, DMC members may have access to unblinded safety data for one or more patients where necessary. However, no members of the study team at INOTREM, at the Contract Research Organization (CRO) or the sites will have access to unblinded data until after database lock is declared. In case unblinded data needs to be handled or analyzed before database lock, these tasks will be performed by persons independent from the study team (e.g. unblinded statistician at the CRO).

8 DMC MEETINGS

8.1 Meeting Schedule

DMC meetings will be held at the following timepoints:

- Initial meeting for the set-up of the DMC and its procedures (e.g. charter, analysis plans)
- Part 1:
  - Unblinded scheduled meetings after every 20 randomized patients (i.e. after 20 and 40 patients) completed day 14 after randomization for the assessment of

DocuSign Envelope ID: E826A649-51DD-4828-9171-DF850DA937EA

safety. A first DMC review will occur after the first 20 patients (10 nangibotide and 10 placebo) have completed the 14 days period after randomization. The DMC review process will be repeated after 40 and 60 patients have completed the 14 days period after randomization.

- Part 2:
  - In the second part of the study, unblinded scheduled meetings (interim analyses for futility) will take place after a total of 130 and 250 patients have completed day 28 after randomization (parts 1 and 2 combined).
  - After 370 patients have been recruited, final assessment of the part 2 primary outcome will be undertaken. In addition, the DMC will conduct an interim futility analysis for mortality after 370 patients have been recruited and completed day 28 after randomisation.
- Part 3:
  - There will be no further scheduled DMC reviews

- Additional or unscheduled meetings may take place throughout the study as required.

8.2 Format and Scheduling of Meetings

The preferred meeting format will be teleconference/videoconference. However, face-to-face meetings may be conducted, depending on feasibility. The meeting will consist of an open and a closed part. During the open part, the sponsor will present available data and provide responses to questions. During the subsequent closed part, only the DMC members will discuss the data and decide on recommendations to the sponsor. The DMC may consult with the unblinded statistician during the closed meeting. The closed session discussions are confidential.

The meetings will be scheduled by the DMC chair in cooperation with PPD DMC Coordination and the sponsor. Effort should be made to keep the timelines between the data lock point and the DMC recommendations as short as possible. The sponsor will communicate the current study status on a regular basis and agree timelines with the DMC for their review in advance.

The content of the DMC briefing packages will be agreed between the sponsor and the DMC chair and may be adjusted during the course of the study. This will be documented in a separate document.

The data listing reports will be provided to the DMC members prior to the scheduled meeting. Due to the nature of the study, these may be available only on a short notice. Efforts will be made to provide these at least 24 hours prior to the meeting. INOTREM will liaise with the DMC chair to ensure availability of the DMC members for review.

8.3 Initial DMC Meetings

The first meeting of the DMC will be an organizational meeting. It will be held during the final stage of study set-up, to provide advisory review of the scientific and ethical issues relating to the study design and conduct, to discuss the standard operating procedures for the role and functioning of the DMC, and to discuss the format of reports that will be used to present trial data at future DMC meetings.

8.4 Unblinded DMC Meetings for the Assessment of Interim Analyses

In part 1 of the study, unblinded DMC meetings are scheduled after 20, 40 and 60 randomized patients have completed day 14 after randomization for the assessment of safety.

In part 2, unblinded review will take place as soon as possible after the appropriate patients reaches day 28.

8.4.1 Interim Assessments

After the completion of day 14 of the last patient of the respective review point, the sponsor will provide a report with cumulative safety data for the DMC to review. The «data lock point» is defined as day 14 of the last patient for the respective timepoint in part 1 of the study and day 28 of part 2 of the study.

The following data will be required:

**Data**	**Description/Format**
Serious Adverse Events	All **SAE** data as line listing and CIOMS forms
Adverse Events	Tabular report of **all adverse events**, available in the eCRF system 24 hours after the data lock point. Sites should make effort to complete data entry for safety data for all patients until then.
Demographics and Baseline Characteristics	• Demographics data listing<br>• Baseline characteristics data listing
Clinical Laboratory	Tabular report of the parameters available in the eCRF system:<br>• Chemistry: Aspartate aminotransferase, alanine aminotransferase, alkaline phosphatase, total bilirubin, creatinine, urea, glucose, sodium, potassium, chloride, calcium, inorganic phosphate, total protein, albumin, lactate, procalcitonin<br>• Haematology: Hemoglobin, hematocrit, leucocytes, basophils, eosinophils, neutrophils, lymphocytes, monocytes, platelet count<br>• Coagulation: International Normalized Ratio (INR), disseminated intravascular coagulation (DIC) score<br>• Pregnancy test.
Patient narratives	Narratives (discharge letters) of for all patients if available
**Futility analyses**	
Clinical Status	• Clinical status of included patients at Day 1(before IMP) and subsequent study days to day 28<br>• Change in clinical status from baseline at a number of study timepoints including day 28
Mortality	Incidence and date of death when reported to day 28
Organ support	• Duration of organ support (ventilation, renal and vasopressor)<br>• Days free of organ support (respiratory, renal and vasopressor)<br>• Respiratory support will be categorized by invasive and non invasive respiratory support
Additional data	• Additional clinical data required for analysis of safety or futility as requested by DMC members

Details on the briefing documentation such as tables, listings and figures will be defined in a separate document (Appendix 2).

DocuSign Envelope ID: E826A649-51DD-4828-9171-DF850DA937EA

8.4.2 Interim assessment of safety

The decision to continue the study will be based upon the comparative incidence of adverse events, serious adverse events and death in the two study groups.

8.4.3 Interim analyses of safety and efficacy (Part 2 only):

Unblinded data will be reviewed by an independent DMC. A DMC review of both safety and efficacy will occur after the first 130 patients have completed the 28-day period after randomization. All patients who received at least one dose and were scheduled for completion of the 28 days period, including early withdrawals, will be included in this count.

The DMC review of both safety and efficacy will be repeated after an additional 120 (250 total) patients have completed the 28 days period after randomization. Futility and progression rules for each analysis will be detailed in the DMC SAP.

A DMC review of both safety and efficacy will occur after the 370 patients have completed the 28-day period after randomization. All patients who received at least one dose and were scheduled for completion of the 28-day period, including early withdrawals, will be included in this count. After 370 patients have been recruited, final assessment of the part 2 primary outcome will be undertaken.

At the same time, the DMC will conduct an interim futility analysis for mortality after 370 patients have been recruited. If the absolute difference in mortality is less than the proposed futility boundary, the study will be stopped. If the overall incidence of death at day 28 is higher or the difference in death rates between groups is less than expected but still of clinical relevance, the DMC may recommend an adaptive increase in sample size beyond the planned part 3 total (i.e. 730 patients) to maintain the power at the planned target as much as possible.

Safety will be assessed at each interim analysis.

8.5 Additional DMC Meetings

Additional meetings of the DMC can be scheduled by the DMC chair or the sponsor as deemed necessary for the assessment and discussion of safety aspects.

9 DMC MEETING MINUTES

The DMC will prepare closed (unblinded) minutes of their meeting. The minutes will be signed by all DMC members and originals kept by the Chairperson until unblinding of the study. Following each meeting, summary minutes will be drafted and distributed within 2 business days by the Chairperson to the other DMC members. PPD may assist with the drafting and circulation of the meeting minutes as deemed necessary by the DMC chair. The minutes will be reviewed and approved by the DMC members within 5 business days of distribution and signed by the chair of the DMC. The Final minutes will be kept securely by the DMC chair and transferred to the sponsor after database lock.

10 DMC RECOMMENDATIONS

The DMC recommendations after each DMC meeting will include one of the following:

- Continuation of the study without modifications
- Continuation of the study with modifications. The recommended modifications may be within or beyond the adaptive feature of the study protocol
- Request for additional information

DocuSign Envelope ID: E826A649-51DD-4828-9171-DF850DA937EA

- Suspension of the study (put study “on-hold”) on grounds of safety for consultation with regulatory authorities
- Discontinuation of the study on grounds of safety

The DMC chair will inform the sponsor in a blinded fashion about the DMC recommendations immediately after the meeting via the DMC Chair Recommendation form (Appendix 3). The document may be sent as an attachment to the Sponsor with PPD DMC Coordinator in copy.

11 RECORD RETENTION

The PPD DMC coordination team will retain copies of all meeting minutes, notes, copies of safety reports and findings, vote results, recommendations and reports used in the evaluation of safety of protocol until database lock is declared by the sponsor, at which time the materials will be transferred into the PPD eTMF.

12 REPORTING OF STUDY PROGRESS AND IMPORTANT EVENTS TO DMC

The sponsor will report the progress of the study to the DMC members on a regular basis. The following important events will be reported to the DMC immediately:

- Adverse events and Serious Adverse Events on a monthly basis
- All suspected unexpected serious adverse reactions (SUSARs)
- Quarterly monitoring reports from pharmacovigilance provider

13 COMMUNICATION POLICY

It is the responsibility of the Sponsor to communicate with the sites and ethics committees (where required) on a regular basis. The initial communication will include a copy of this charter and previous DMC recommendations.

Country specific procedure in France: The sponsor will put a process in place for prompt submission of DMC meeting minutes and recommendations to ANSM by the DMC.

14 ACCEPTANCE OF DMC RECOMMENDATIONS

It should be understood that the DMC represents an advisory body for the Sponsor. While it is unlikely that the sponsor would ignore DMC recommendations, the company is not obliged to follow DMC recommendations. Thus, upon receiving a DMC recommendation, the Sponsor will decide upon appropriate potential steps in consultation with the DMC, and and/or relevant regulatory authorities, as required. It is understood that the Sponsor can elect to continue, interrupt, or close the study even if the DMC were to recommend a different course of action.

If the DMC recommends study interruption/termination, the Sponsor may also request consultation from relevant regulatory authorities.

Once a DMC recommendation to stop the study is accepted, the Sponsor will be responsible for notifying regulatory agencies, investigators, and ethics committees of the DMC’s recommendations and the company’s decision to stop the study. These activities will be performed consistent with GCP guidelines and relevant regulatory requirements.

If the DMC recommends that the trial be stopped, but the Sponsor decides to continue the trial, the Sponsor will provide a written explanation of the decision to the DMC within 14 business days. If a DMC recommendation to stop the study is rejected by the Sponsor, the company will be responsible for notifying regulatory agencies, investigators and ethics committees of the DMC’s recommendations and the company’s decision to continue the study. These activities will be performed consistent with GCP guidelines and relevant regulatory requirements.

DocuSign Envelope ID: E826A649-51DD-4828-9171-DF850DA937EA

The Sponsor or the investigators are at liberty to interrupt or close the study at one or more sites without consulting the DMC. However, it is expected that the DMC would be notified of such actions.

DocuSign Envelope ID: E826A649-51DD-4828-9171-DF850DA937EA

APPENDIX 1: DMC MEMBERS AND CONTACT INFORMATION

Michel Wolff, MD - DMC Chair
Service d'Anesthésie-Réanimation Neurochirurgicale
Pôle Neuro Sainte Anne
GHU Paris, Psychiatrie & Neurosciences
1, rue Cabanis
75674, Cedex 14, Paris, France
Tel : + 33 1 45 65 86 41
Mobile : + 33 6 87 33 28 45
Email: m.wolff@ghu-paris.fr and michel.wolff50@gmail.com

Jean Chastre, MD
Service de Réanimation Médicale
Institut de Cardiologie
Groupe Hospitalier Pitié-Salpêtrière
47-83 boulevard de l'Hôpital
75651 Paris Cedex 13, France
Tel: +33 142163822
Fax: +33 142163811
Email: jean.chastre@aphp.fr

Steven Opal, MD
Clinical Professor of Medicine, Rhode Island Hospital, Infectious Disease Division, Alpert Medical School of Brown University
Co-Director, Ocean State Clinical Coordinating Center, 1 Virginia Avenue, Suite 105, Providence, RI 02905, USA
Tel: +1-401-575-1814
Fax +1-401-444-1489
Email: Steven_Opal@brown.edu

Stephen Senn, PhD - Statistician
29 Merchiston Crescent
Edinburgh
EH10 5AJ
UK
+44 7811166929
stephen@senns.uk

DocuSign Envelope ID: E826A649-51DD-4828-9171-DF850DA937EA

APPENDIX 2: SAFETY DATA LISTINGS FOR DMC REVIEW

Provided as separate document

DocuSign Envelope ID: E826A649-51DD-4828-9171-DF850DA937EA

APPENDIX 3: DMC CHAIR RECOMMNEDATION FORM

Inotrem MOT-C-204

DATA MONITORING Committee

CHAIR RECOMMENDATION FORM

Date of Meeting: _________________ **DMC Chair:** (*Insert Chair Name*)

(DD MMM YYYY)

**The DMC recommendations below are based on a comprehensive evaluation of the data presented, their medical expertise, and their participation in the DMC meeting. The DMC members understand that the recommendations are based on a vote.**	
**Were any safety concerns identified which would lead the DMC to recommend changes to the conduct of the study?**<br>*(Please specify under recommendations)*	Yes ☐ No ☐
**Is additional follow up requested?**<br>*(Please specify under recommendations)*	Yes ☐ No ☐
**Should the study continue?**	Yes ☐ No ☐
**Recommendations:**	
**DMC Comments:**<br><br>Chair Signature______________________ Date___ ___________	
Inotrem Response (attach documents if necessary)<br><br>***Inotrem signature*****_______________________** ***Date*****_______________**	
