# Supplementary material for: Evaluation of the efficacy and safety of TREM-1 inhibition with nangibotide in patients with COVID-19 receiving respiratory support: the ESSENTIAL randomised, double-blind trial
Source: eClinicalMedicine. 2023 May 31;60:102013. doi: 10.1016/j.eclinm.2023.102013 (PMC10231876; doi:10.1016/j.eclinm.2023.102013)
Supplement: ESM4 INOMOTC204 DMC SAP [file mmc3.pdf]

INOTREM

MOT-C-204 ESSENTIAL

Exploratory study of the safety, tolerability and efficacy of nangibotide in patients with COVID-19 receiving ventilatory support and features of systemic inflammation

A randomized, double-blind, placebo-controlled study with adaptive features

14DEC2021

DMC Statistical Analysis Plan of IA number 1 and IA number 2 of Study Part 2

Version 1.0

Prepared by:

PPD
Granta Park, Great Abington,
Cambridge, CB21 6GQ

TABLE OF CONTENTS

LIST OF ABBREVIATIONS IV

1. INTRODUCTION 1

2. OBJECTIVES 1

2.1. PRIMARY 1
2.2. SECONDARY 1

3. INVESTIGATIONAL PLAN 2

3.1. DATA MONITORING COMMITTEE 2
3.2. TREATMENTS 2
3.3. STUDY ENDPOINTS RELEVANT TO INTERIM ANALYSES 3
3.3.1. First and second interim analysis in part 2 3
3.3.2. Futility endpoints 3
3.3.3. Secondary efficacy and safety endpoints 3
3.3.4. End of part 2: third interim analysis 3

4. GENERAL STATISTICAL CONSIDERATIONS 3

4.1. TABULATIONS AND PRESENTATIONS 3
4.2. SAMPLE SIZE 5
4.3. RANDOMIZATION, STRATIFICATION, AND BLINDING 5
4.3.1. Randomization 5
4.3.2. Stratification 6
4.3.3. Blinding 6
4.4. ANALYSIS SET 6
4.4.1. Safety Set 6
4.4.2. All Randomized Patients (Intent-to-Treat (ITT)) Set 6
4.4.3. Modified Intent-to-Treat (mITT) Set 6
4.4.4. Per Protocol (PP) Set 6

5. PATIENT DISPOSITION 7

5.1. DISPOSITION 7

6. DEMOGRAPHICS AND BASELINE CHARACTERISTICS 7

6.1. DEMOGRAPHICS 7
6.2. BASELINE DISEASE CHARACTERISTICS 7
6.3. MEDICAL HISTORY 8

7. TREATMENTS AND MEDICATIONS 8

7.1. PRIOR AND CONCOMITANT MEDICATIONS 8
7.1.1. Prior medications related to COVID-19 9
***7.1.2. Concomitant medications related to COVID-19** 9*
7.2. EXTENT OF EXPOSURE 9

8. EFFICACY ANALYSES 9

8.1. ANALYSIS OF THE PRIMARY ENDPOINT OF PART 2: CLINICAL STATUS 9

8.2. ANALYSIS OF THE PRIMARY ENDPOINT OF PART 3, I.E. MORTALITY AT DAY 28 (ACM28) 12
8.3. EXPLORATORY EFFICACY ANALYSES FOR FIRST AND SECOND IA 13
8.3.1. Days free of organ support (respiratory, renal and vasopressor) 13
8.3.2. Respiratory support type 13
8.4. FUTILITY BOUNDARIES 14
8.4.1. Futility boundaries for the clinical status 14
8.4.2. Futility boundaries for All-Cause Mortality at Day 28 14
8.5. DECISION RULES TO STOP/CONTINUE THE TRIAL 15
8.5.1. First interim analysis 15
8.5.2. Second interim analysis 16
8.5.3. Third interim analysis/End of part 2 analysis 16

9. SAFETY ANALYSIS 16

9.1. ADVERSE EVENTS 16
9.1.1. Treatment Emergent Adverse Event 17
9.1.2. Incidence of adverse events 17
9.1.3. Relationship of adverse events to study drug 17
9.1.4. Serious Adverse Events 18
9.1.4.1. Serious Adverse Events, related to study drug 18
9.1.5. Adverse events leading to treatment discontinuation 18
9.1.6. Death 18
9.1.7. Overview summary 19
9.2. CLINICAL LABORATORY EVALUATIONS 19
9.2.1. Hematology 19
9.2.2. Plasma biochemistry 19
9.2.3. Coagulation 19
9.2.4. Pregnancy test 19
9.3. SECONDARY INFECTION AND INFECTION TYPE 19
9.4. CO-ADMINISTERED IMMUNOMODULATORY MEDICATIONS, DEATHS, SAES AND SECONDARY INFECTIONS 20

10. REFERENCES 21

11. APPENDICES 22

11.1. SCHEDULE OF STUDY PROCEDURES 22

11.2. INTERIM LIST OF OUTPUTS FOR FIRST AND SECOND INTERIM ANALYSIS 24

List of Abbreviations

AE	Adverse Event
AR	Adverse Reaction
ATC	Anatomical Therapeutic Chemical
CI	Confidence Interval
COVID-19	Coronavirus disease 2019
CoV	Coronavirus
CRF	Case Report Form
CRP	C Reactive Protein
d/D	Day
DMC	Data Monitoring Committee
ECMO	Extracorporeal Membrane Oxygenation
EoS	End of Study
EoT	End of Treatment
FU	Follow-up
h/H	Hour
IA	Interim Analysis
ICH	International Council for Harmonisation of Technical Requirements for Pharmaceuticals for Human Use
ICU	Intensive Care Unit
IL-6	Interleukin-6
IMV	Invasive Mechanical Ventilation
IMP	Investigational Medicinal Product
INR	International Normalized Ratio
IQR	Interquartile Range
ITT	Intent-to-Treat
MedDRA	Medical Dictionary for Regulatory Activities
MH	Mantel-Haenszel
MITT	Modified Intent-to-Treat
PCT	Procalcitonin
PD	Pharmacodynamic
PP	Per Protocol
PT	Preferred Term
SAE	Serious Adverse Event
SAP	Statistical Analysis Plan
SD	Standard Deviation
SOC	System Organ Class
SOFA	Sequential Organ Failure Assessment
sTREM-1	Soluble TREM-1
TLFs	Tables, Listing and Figures
TEAE	Treatment-Emergent Adverse Events
TREM-1	Triggering Receptor Expressed on Myeloid Cells
WHODRUG	World Health Organization Drug Dictionary

1. Introduction

This study with an adaptive design was initially designed to assess safety and tolerability of nangibotide in patients with COVID-19 receiving ventilatory support and features of systemic inflammation with a sample size of 60 patients. An extension of the study that will also allow for appropriate assessment of efficacy was approved through protocol amendment 5 and after review of safety and tolerability on the 60 first patients that form the part 1 of the study.

According to protocol amendment 5 the study is now divided into three parts:

Part 1: A safety and tolerability study has completed recruitment of 60 patients. An independent Data Monitoring Committee (DMC) conducted an analysis of safety and tolerability and recommends continuation of part 2.
Part 2: Recruitment of 310 new patients including two interim futility analyses and one end of part 2 analysis for progression to part 3 or cessation due to futility.
Part 3: Recruitment of at least a further 360 patients (total 730) to assess the impact of study drug on mortality at Day 28 although this sample size may be increased based upon DMC recommendation.

This statistical analysis plan (SAP) describes the planned interim analyses and DMC safety reviews of the part 2 of the study MOT-C-204 study. Details on the study design can be found in the current version of the study protocol Version 5.0, dated 01MARCH2021. All final analyses for this study will be contained in a separate full SAP document. More information on the DMC, including members, responsibilities, the meetings and reporting can be found in the DMC Charter Version 2.0, dated 01MAR2021. This statistical analysis plan is based on International Council for Harmonization of Technical Requirements for Pharmaceuticals for Human Use (ICH) E3 and E9 Guidelines.

2. Objectives

The full study objectives are as follows:

2.1. Primary

- To evaluate the impact of nangibotide on the progression of disease in patients receiving ventilatory support due to COVID-19

2.2. Secondary

Secondary objectives of the study are to investigate the safety and efficacy of nangibotide in patients receiving ventilatory support due to COVID-19.

This includes:
• To evaluate the effects of nangibotide exposure over 5 days in patients with COVID-19

• To evaluate the effect of nangibotide on clinical parameters
• To evaluate the safety and tolerability of nangibotide in patients with COVID-19
• To evaluate PD relationship to TREM-1 pathway related markers
• To evaluate the natural history of the disease regarding the activation of the TREM-1 pathway measured by soluble TREM-1 plasma concentrations over time
over time

Next sections of this document more specifically focus on part 2 of the study.

3. Investigational Plan

3.1. Data Monitoring Committee

In part 2, the DMC will monitor the safety of the MOT-C-204 study and assess the study for potential futility at the following scheduled timepoints:

**Timepoint**	**Assessment**
130 (60 from part 1 and 70 from part 2) patients have completed the 28-day period after randomization.	Safety, Efficacy futility on clinical status 7-point ordinal scale and mortality at Day 28.
250 patients have completed the 28-day period after randomization	Safety, Efficacy futility on clinical status 7-point ordinal scale and mortality at Day 28.
370 patients have completed the 60-day period after randomization	Safety, Efficacy on clinical status 7-point ordinal scale and futility on mortality at the end of part 2 for progression to part 3.

Additional meetings of the DMC can be scheduled by the DMC chair or the sponsor as deemed necessary for the assessment and discussion of safety aspects.

Appendix 11.2 contains the list of outputs that will be included for the above interim analyses.

3.2. Treatments

In all parts of the study, the intervention will remain the same.
Patients will receive a continuous intravenous (i.v.) infusion of nangibotide at 1.0 mg/kg/h or a matching placebo. Treatment with study drug must be initiated as early as possible but no later than 48 hours after the initiation of ventilatory support. Patients will be treated for 5 days or until discharge from critical care, whichever is sooner. The treatment with study drug will be in addition to standard of care. Follow-up visits will be performed on days 8 and 14. The end of study visit is at Day 28. A further follow up visit will be undertaken on day 60.

3.3. Study Endpoints Relevant to Interim Analyses

3.3.1. First and second interim analysis in part 2

3.3.2. Futility endpoints

- Primary endpoint of part 2: Clinical status 7-point ordinal scale at Day 28
- Key secondary endpoint of part 2: All-cause mortality at Day 28

3.3.3. Secondary efficacy and safety endpoints

- Clinical status as improvement from baseline (Yes vs No) at Day 28
- Clinical status as a 3-point ordinal scale (change from baseline categorized as improved, unchanged, worsened) at Day 28
- All-cause mortality on day 60
- Days free of organ support (respiratory, renal and vasopressor) at Day 28
- Respiratory support type at Day 28
- Safety laboratory tests (as part of routine clinical care): hematology, coagulation, plasma biochemistry
- Adverse events (AEs), serious adverse events (SAEs) and deaths
- Suspected adverse drug reactions (serious and non-serious)
- Secondary infections

3.3.4. End of part 2: third interim analysis

Details of third IA at the end of part 2 will be provided in a separate SAP.

4. General Statistical Considerations

4.1. Tabulations and Presentations

All summaries and results in Tables, Listings and Figures (TLFs) should be presented for the following treatment groups:

Treatment groups	Label in TLFs
1.0 mg/kg/h continuous intravenous (i.v.) infusion of nangibotide	Nangibotide 1.0 mg/kg/h
Matching placebo	Placebo

Continuous data will be described using descriptive statistics (i.e. n, mean, standard deviation, median, interquartile range (IQR), minimum, and maximum). Categorical data will be described using the patient count and percentage in each category. For the summary statistics of all numerical variables unless otherwise specified, minimum and maximum will be displayed to the same level of precision as reported. Mean, median and IQR will be

displayed to one level of precision greater than the data collected. Standard deviation will be displayed to two levels of precision greater than the data collected.

When count data are presented, the percentage will be suppressed when the count is zero in order to draw attention to the non-zero counts. A row denoted "Missing" will be included in count tabulations where specified on the shells to account for dropouts and missing values. The denominator for all percentages will be the number of patients in that treatment within the Analysis Set of interest, unless otherwise specified. Percentages will be presented to one decimal place.

Any imputed data will be used in summary tables and figures and the original collected data will be displayed in listings. Data will be displayed in all listings sorted by treatment group. Patients will be identified in the listings by the patient identification number concatenated with the investigator number.

Two-sided p-values will be displayed except for the primary analysis of clinical status (7-point ordinal scale) at Day 28 used for the futility decision.

All analyses will be conducted using SAS Version 9.4 or higher.

Baseline and Study Day

Unless otherwise specified, baseline (day 1) will be defined as the last non-missing evaluation prior to or on the date that the first dose of treatment is given.
For visits prior to day of first study dose, study day will be calculated as:
assessment date – first study dose date.
For visits on or after the day of first study dose, the study day will be calculated as:
assessment date - first study dose date + 1.

Visit Windows

All data summarized by visit will be based on the visit name collected on the electronic Case Report Form (eCRF) page. For data from unscheduled visits, these will be listed but not included in any by-visit summaries or analyses.

Missing Start and Stop Dates

For the purpose of inclusion in prior and/or concomitant medication and AE tables, incomplete medication start and stop dates will be imputed as follows:

Missing start dates (where UN, UNK and UNKN indicate unknown or missing day, month and year respectively):

- UN-MMM-YYYY: If the month and year are different from the month and year of the first dose of study drug, assume 01-MMM-YYYY. If the month and year are the same as the first dose of study drug month and year and the end date (after any imputation) is on or after the first dose of study drug, then assume the date of the first dose of study drug. If the month and year are the same as the first dose of study drug

month and year and the end date (after any imputation) is prior to the first dose of study drug, then assume the end date for the start date;

- DD-UNK-YYYY/UN-UNK-YYYY: If the year is different from the year of the first dose of study drug, assume 01-JAN-YYYY of the collected year. If the year is the same as the first dose of study drug year and the end date (after any imputation) is on or after the first dose of study drug, then assume the date of the first dose of study drug. If the year is the same as the first dose of study drug and the end date (after any imputation) is prior to the first dose of study drug, then assume the end date for the start date.

Missing stop dates (where UN, UNK and UNKN indicate unknown or missing day, month and year respectively):

- UN-MMM-YYYY: Assume the last day of the month;
- DD-UNK-YYYY/UN-UNK-YYYY: Assume 31-DEC-YYYY.

In case of the death of the patients and the imputed end date is after the date of death, the stop/end date will be imputed as the date of death.

4.2. Sample Size

A total of 370 patients are planned to be enrolled at end of part 2. Approximately 360 additional patients are planned to be enrolled if study progress to part 3 (total sample size 730). Details regarding sample size determination are given in the protocol.

The DMC will conduct an analysis of efficacy and an interim futility analysis for mortality at the end of part 2, i.e. after 370 patients have been recruited. If the absolute difference in mortality is less than the proposed futility boundary, the study will be stopped. If the overall incidence of death at Day 28 is higher than expected (i.e. resulting in a larger variance) or the difference in death rates between groups is less than expected but still of clinical relevance promising, the DMC may recommend an adaptive increase in sample size beyond the planned part 3 total (i.e. 730 patients) to maintain the power at the planned target as much as possible. Type 1 error will be kept at the usual 0.025 1-sided level for final analysis using the Cui Hung and Wang (1999) approach for sample size reassessment.

4.3. Randomization, Stratification, and Blinding

4.3.1. Randomization

The randomization scheme will be generated by an independent statistician who is not part of the study team. A randomization number will be assigned to each patient. The randomization scheme will be generated by a block randomization scheme.

In stage one part 1 of the study, eligible patients were randomized in a 1:1 ratio into 1 of the 2 treatment arms (20 first patients). In stage 2 part 1 of the study, patients were allocated to nangibotide treatment or placebo in a 3:1 ratio resulting in 20 subjects to be randomized on

Placebo and 40 subjects randomized on nangibotide at the end of part 1. In part 2 and 3 eligible patients will be randomized in a 1:1 ratio into 1 of the 2 treatment arms.

4.3.2. Stratification

The randomization will be stratified according to site.

4.3.3. Blinding

The study will be conducted in a double-blind fashion, whereby patients, clinical study site staff as well as sponsor and CRO personnel involved in the monitoring and evaluation of the study are blinded to study drug assignment.

Study drug and matching placebo will be provided as individually numbered vials in secondary packaging of identical appearance.

Upon completion of the study, after the database lock and after the blind is revealed, the randomization list will be filed in the Trial Master File.

4.4. Analysis Set

The Safety analyses will be based on the Safety Set and the Efficacy interim analyses will be based on mITT Set.

4.4.1. Safety Set

All enrolled patients having received at least one dose of the prescription of study medication (either nangibotide or placebo). The Safety Set will be the basis for safety analyses. Patients will be assigned to the treatment group as treated for the Safety Set. Patients receiving at least one dose of nangibotide will be reported in the nangibotide group.

4.4.2. All Randomized Patients (Intent-to-Treat (ITT)) Set

ITT set will include all patients who are randomized into the study and considered as randomized, i.e. with study drug assignments designated according to initial randomization, regardless of whether patients received what was assigned.

4.4.3. Modified Intent-to-Treat (mITT) Set

All randomized patients having received at least one dose of the prescription of study drug (either nangibotide or placebo). Patients will be assigned to the treatment group as randomized (i.e. with study drug assignments designated according to initial randomization, regardless of whether patients received what was assigned). The mITT Set will be considered as the primary set for interim and final efficacy analyses.

4.4.4. Per Protocol (PP) Set

All mITT patients without any major protocol deviation. Major protocol deviations will be identified during data review meetings that will be held before unblinding and database lock.

The PP Set will be considered as support analysis set for specific predefined endpoint in final efficacy analyses that will be described in Final SAP and will not be used for any interim analysis on which this interim SAP focuses.

5. Patient Disposition

5.1. Disposition

Patient disposition will be summarized using the All Enrolled Set. The total number of patients will be summarized for the following categories: those who were screened, and those who were not randomized. The number and percentage of patients within each treatment group will be presented by the following categories: randomized, received study drug, did not receive study drug, completed study drug, discontinued study drug (and reason), completed study, and withdrawn from study (and reason). Percentages will be based on the number of patients randomized.

Patient disposition data will be presented in a listing.

6. Demographics and Baseline Characteristics

6.1. Demographics

Demography data: age (years), sex and ethnic factors will be summarized by treatment group and overall for the Safety Set.

The following baseline characteristics will be summarized for the same analysis set:

- Patient characteristics: baseline height (cm), baseline weight (kg) and BMI (kg/m^2). Fertility status will also be presented for female patients.

Age, baseline height, baseline weight and BMI will be summarized using descriptive statistics. The number and percentage of patients by sex (Male, Female), ethnic factors (White or Caucasian, Black or African American, Asian, Hispanic, Latino or of Spanish Origin, American Indian or Alaska Native, Native Hawaiian or Other Pacific Islander, and Other Ethnic Factors) and fertility status (Childbearing potential; Yes, No, Unknown and Fertility Status; Sterile, Post-Menopausal, Potentially Able to Bear Children), will also be reported. Percentages will be based the total number of patients in the Safety set.

This data will be listed for the Safety Set.

6.2. Baseline Disease Characteristics

The type of ventilation the patients are on at baseline and whether this is invasive mechanical ventilation or not, the baseline clinical status and the baseline sTREM-1 level (above or equal to the median vs below) will be presented by treatment group and overall for the Safety set.

The following categories of mechanical ventilation are considered invasive:

- Ventilation requiring intubation
- ECMO
- Extracorporeal CO2 clearance

The following categories of mechanical ventilation are considered non-invasive:

- Non-invasive ventilation
- High flow

6.3. Medical History

Medical history will be coded using Medical Dictionary for Regulatory Activities (MedDRA) version 23.0 or higher. Medical history will be listed for the Safety Set.

7. Treatments and Medications

7.1. Prior and Concomitant Medications

All medications will be coded according to the World Health Organization drug dictionary (WHODRUG) March 2020, or higher.

A prior medication is defined as any medication with a start date that is prior to the date of the first study drug dose.

A concomitant medication is defined as any medication that has a start and stop date that is on or after the first study drug dose date or that has a start date prior to the first study drug dose date but an end date that is after the first study drug dose date.

For the purpose of inclusion in prior and/or concomitant medication tables, incomplete medication start and stop dates will be imputed as described in Section 4.1.

If start date is completely missing and end date is after first dose intake or completely missing, then the medication will be classified as both prior and concomitant. If the start date is completely missing and the end date is prior to the first dose of study drug, then the medication will be classified as prior. If the end date is missing and the start date is after first dose intake, then the medication will be considered as ongoing and classified as concomitant. If the end date is missing and the start date is before first dose intake, then the medication will be considered as ongoing and classified as both prior and concomitant.

Prior and concomitant medications related to COVID 19 will be presented in a listing for the Safety Set. Therapies will be flagged as prior (P), concomitant (C) or prior and concomitant (PC). Prior and concomitant medications related to COVID-19 will be selected based on CRF data using ‘For COVID-19’ item in the ‘Previous and Concomitant Therapies’ Form. Vasopressors collected in a specific form will be listed separately.

7.1.1. Prior medications related to COVID-19

The total number of prior medications related to COVID-19 and the number and percentages of patients with at least one prior medication related to COVID-19 will be summarized by treatment group and overall. The number and percentages of prior medications related to COVID-19 will be summarized by anatomical therapeutic chemical (ATC) classification category (level 4), preferred term, treatment group and overall for the Safety Set.

7.1.2. Concomitant medications related to COVID-19

The total number of concomitant medications related to COVID-19 and the number and percentages of patients with at least one concomitant medication related to COVID-19 will be summarized by treatment group and overall. The number and percentages of concomitant medications will be summarized by ATC classification category (level 4), preferred term, treatment group and overall for the Safety Set.

7.2. Extent of Exposure

Duration of exposure to study drug will be calculated in hours as:

Date and time of end of study drug infusion – date and time of start of study drug infusion.

Duration of exposure to study drug will be summarized by treatment group for all patients in the Safety Set.

The number and percentage of patients who had infusion interruptions will be summarized along with categorical summary of the number of interruptions for patients in the Safety Set.

All study drug information including reasons for drug interruptions will be listed for patients in the Safety Set.

8. Efficacy Analyses

The m-ITT set will be used for all interim efficacy analyses.

Three interim analyses (IA) in part 2 are planned at 130, 250 and 370 patients.

Interim decision rules to stop/continue the trial at the first and second IA will mainly be based on the analysis of the primary endpoint of part 2, i.e. the clinical status (7-point ordinal scale) assessed at Day 28 AND the analysis of mortality assessed at Day 28.

Interim decision rules to stop/continue the trial at the third IA (at the end of part 2) will be based on the analysis of the primary endpoint of part 3, i.e. mortality assessed at Day 28.

8.1. Analysis of the Primary Endpoint of Part 2: Clinical Status

The clinical status (7-point ordinal scale) at Day 28 will be compared between treatment group using a CMH stratified by baseline clinical status. At baseline, patients are

hospitalized on invasive mechanical ventilation or ECMO (Clinical status = 6) or hospitalized, on non-invasive ventilation or high flow oxygen devices (Clinical status = 5).

Patients with a missing clinical status at Day 28 visit and who died prior to or on Day 28 will be assigned the category of 7 at Day 28. Patients with a missing clinical status at Day 28 visit and without documented death occurring prior to or on Day 28 will be assigned the last available clinical status.

The distribution (counts and percentages) of the 7-point ordinal scale will be displayed by treatment group and baseline level (5 or 6) (i.e., the 2x2x7 contingency table).

The 7-point ordinal scale will be compared between treatment groups using a Cochran-Mantel-Haenszel test stratified by baseline clinical status (5 or 6) using modified ridit scores. The Wald test one-sided p-value for the difference in raw mean scores will be provided.

<u>Sensitivity analyses:</u>

<u>Primary sensitivity analysis:</u>
The treatment effect will be estimated in an ordinal logistic regression on the 7-point ordinal scale adjusting for treatment and baseline clinical status assuming odds proportionality. The odds ratio for treatment effect with its 95% confidence interval and p-value will be estimated in this model.

As the odds proportionality may not hold (the p-value of the test for odds proportionality will be provided), the 7-point ordinal scale will be dichotomized in six binary endpoints : <= cutoff vs > cut-off: 1 vs >1, <=2 vs >2, <=3 > 3, <=4 vs >4 , <=5 vs > 5, <=6 vs 6 (of note , the last cut-off corresponds to the analysis of mortality at day D28). For each cutoff, treatment groups will be compared by a Cochran-Mantel-Haenszel test stratified by baseline clinical status (5 or 6). Odds ratios (with their 95% confidence intervals and p-values) will also be provided. 2x2x2 contingency tables (counts and percentages) will be provided for each cut-off.

<u>Other sensitivity analyses:</u>

1) The 7-point ordinal scale will be compared between treatment groups using a Cochran-Mantel-Haenszel test stratified by randomization stage (part 1 stage 1, part 1 stage 2 and part 2) and baseline status (5 vs 6). The Wald test p-value for the difference in raw mean scores will be provided.

2) The change in the 7-point ordinal scale from baseline to Day 28 will be categorized in two categories: ‘improved’, ‘not improved’.

The distribution (counts and percentages) of the binary scale will be displayed by treatment group and baseline level (5 or 6) (i.e., the 2x2x2 contingency table).

The treatment groups will be compared by a Cochran-Mantel-Haenszel test stratified by baseline clinical status (5 or 6). The odds ratio (with its 95% confidence interval and p-values) will also be provided.

3) The change in the 7-point ordinal scale from baseline to Day 28 will be categorized in three categories: 'improved', 'unchanged', 'worsened'.

   The distribution (counts and percentages) of the 3-point ordinal scale will be displayed by treatment group and baseline level (5 or 6) (i.e., the 2x2x3 contingency table).

   The 3-point ordinal scale will be compared between treatment groups using a Cochran-Mantel-Haenszel test stratified by baseline clinical status (5 or 6) using modified ridit scores. The Wald test p-value for the difference in raw mean scores will be provided. The treatment effect will be estimated in an ordinal logistic regression on the 3-point ordinal scale adjusting for treatment and baseline clinical status assuming odds proportionality. The odds ratio for treatment effect with its 95% confidence interval and p-value will be estimated in this model.

Subgroup analyses

The clinical status will be described by baseline sTREM-1 level (above or equal to the median vs below).

Contingency tables for the binary scale (improved vs not improved) (2x2x2: baseline status x treatment x binary scale) will be provided by baseline sTREM-1 level. By baseline sTREM-1 level, treatment groups will be compared by a Cochran-Mantel-Haenszel test stratified by baseline clinical status (5 or 6). The common odds ratio with its 95% confidence interval will also be provided.

Contingency tables for the 3-point scale (improved/unchanged/worsened) (2x2x3: baseline status x treatment x 3-point ordinal scale) will be provided by baseline sTREM-1 level. By baseline sTREM-1 level, treatment groups will be compared by a Cochran-Mantel-Haenszel test stratified by baseline clinical status (5 or 6). By baseline sTREM-1 level, the treatment effect will be estimated in an ordinal logistic regression on the 3-point ordinal scale adjusting for treatment and baseline clinical status assuming odds proportionality. The odds ratio for treatment effect with its 95% confidence interval and p-value will be estimated in this model.

8.2. Analysis of the Primary Endpoint of Part 3, i.e. Mortality at Day 28 (ACM28)

The All-Cause Mortality (ACM) summaries and analyses below will be limited to the patients with a Day 28 ACM (ACM28) profile, i.e. at the data cut, only patients that have reached Day 28, or could have reached Day 28, but did not due to early termination or death will be included.

All-Cause Mortality will be assessed using survival status up to study day 31, i.e. any death occurring between the start of study drug infusion and the start of study drug infusion + 30 days.

The number and percentage of patient alive, dead and lost to follow-up at Day 28 will be summarized by treatment group. Any patient with last date known to be alive < Day 28 without death information will be considered lost to follow-up in this summary.

Last known date to be alive will be derived using the following information:

- Date of laboratory samples
- Death information
- Date of End of study visit, only if reason is not death or lost to follow-up
- End date of any adverse events
- 'Date Last known to be alive' documented by the investigator

Inferential analysis will be based on patients which status (alive or dead) is known at Day 28 (i.e. excluding patient lost to follow-up before Day 28). Missing data will not be imputed.

Mortality rate at Day 28 will be compared between treatment group using a CMH adjusted for baseline clinical status (5 vs 6). The common difference in mortality rates between nangibotide and placebo (nangibotide ACM28 rate - placebo ACM28 rate) will be estimated with its 95% confidence interval using the Mantel-Haenszel method. Hence, a negative difference in ACM28 rates favors nangibotide.

Sensitivity analysis :

1) The mortality rate at Day 28 will be compared between treatment group using a CMH adjusted for baseline clinical status (5 vs 6) and randomization stage (part 1 stage 1, part 1 stage 2 and part 2). The adjusted difference in mortality rates between nangibotide and placebo (nangibotide ACM28 rate - placebo ACM28 rate) will be estimated with its 95% confidence interval using Mantel-Haenszel method.

2) Kaplan-Meier estimates of mortality will also be estimated at Day 28

3) The same analyses mentioned above (including sensitivity analyses) will be performed at Day 60.

Subgroup analyses :

ACM28 will be described by baseline sTREM-1 level (above or equal to the median vs below). Contingency tables for ACM28 will be provided by baseline sTREM-1 level.
Mortality rate at Day D28 by baseline sTREM-1 level will be compared between treatment group using adjusting for baseline clinical status (5 vs 6). The common difference in mortality rates between nangibotide and placebo (nangibotide ACM28 rate - placebo ACM28 rate) will be estimated with its 95% confidence interval using the Mantel-Haenszel method.

The same analysis will be performed for ACM at Day 60.

8.3. Exploratory Efficacy Analyses for First and Second IA

Descriptive analyses will be provided only.

8.3.1. Days free of organ support (respiratory, renal and vasopressor)

The number of days free of organ support (respiratory, renal and vasopressor) will be calculated as the total number of calendar days from treatment start up (Day 1) to Day 28, when a patient is alive and does not require any organ support. If a patient died on or before Day 28, the number of days will be set to 0. For patients withdrawn alive before Day 28, number of days will be calculated until day of withdrawal. The mean, median, Q1 and Q3, min and max of the distribution of the number of days free of organ support will be provided by treatment group overall and by baseline clinical status.

8.3.2. Respiratory support type

The number and percentage of patients under each kind of ventilation at Day 28 and the number and percentage of patients under invasive mechanical ventilation at Day 28 will be described by treatment overall and by baseline clinical status.

The following categories of mechanical ventilation are considered invasive:

- Ventilation requiring intubation
- ECMO
- Extracorporeal CO2 clearance

The following categories of mechanical ventilation are considered non-invasive:

- Non-invasive ventilation
- High flow

Subjects who died or are lost to follow-up before Day 28 will be excluded from these summary tables.

8.4. Futility Boundaries

Interim decision rules to stop/continue the trial at the first and second IAs will mainly be based on the analysis of the primary endpoint of Part 2, i.e., the clinical status (7-point ordinal scale) assessed at Day 28 AND the analysis of the primary endpoint of Part 3, i.e., mortality assessed at Day 28.

Interim decision rules to stop/continue the trial at the third IA (at the end of part 2) will be based on the analysis of the primary endpoint of Part 3, i.e., mortality assessed at Day 28 and will be described in a separate SAP.

8.4.1. Futility boundaries for the clinical status

Futility Boundaries are expressed in terms of futility p-values for the clinical status (7-point ordinal scale) and in terms of difference in mortality rates for ACM28.

Although the analysis of the clinical status and the p-value will be based on the 7-point ordinal scale (i.e. the Wald test one-sided p-value for the difference in raw mean scores from a Cochran-Mantel-Haenszel test stratified by baseline clinical status (5 or 6) using modified ridit scores), the futility p-values are calculated assuming:

- an improvement odds ratio of 2.27 (55% with nangibotide and 35% with placebo)
- a 1-sided alpha of 0.025
- a planned total sample size of 360 patients (of note, given the imbalanced randomization ratio at stage 2 of part 1 and the subsequent loss of efficiency, the first two interim analysis final analysis and the final analysis are planned at 130,250 and 370 patients respectively).
- Two interim analyses performed at 120 and 240 patients respectively (information fractions of 1/3 and 2/3 respectively).
- A beta spending function from the gamma family with parameter 0.

Based on these considerations, the futility **1-sided futility p-values** are **0.459 and 0.126 for the first and second interim analyses** conducted at 130 and 250 patients respectively.

The power of the analysis of the clinical status is at least 94.4% assuming that the analysis based on the 7-point ordinal scale is more powerful than the analysis of the dichotomized clinical status (improvement vs not improvement).

Of note, at the final analysis (i.e., at 370 patients), the 1-sided p-value will be compared to **the usual 1-sided level of 0.025**.

8.4.2. Futility boundaries for All-Cause Mortality at Day 28

First, second and third interim analyses: they will be performed at 130, 250 and 370 patients respectively.

The futility boundaries will be expressed in terms of difference in mortality rates (nangibotide ACM28 rate - placebo ACM28 rate). Hence, a positive difference in ACM28 rates favors the placebo group.

Given the imbalance in allocation at stage 2 of part 1, calculations were performed at 120, 240 and 360 patients with a planned horizon of 720. The futility boundary will be taken equal at each interim analysis, i.e. equal to -0.02 = -2% in favor of nangibotide (absolute value).

8.5. Decision Rules to Stop/Continue the Trial

Although the decision of claiming futility will primarily be based on the prespecified rules described below, all sensitivity efficacy and safety analyses described in this document should also be taken into consideration when making the final decision to stop the trial.

8.5.1. First interim analysis

Futility rules for claiming futility at the first interim analysis (at ~ 130 patients) are as follows:

The interim one-sided p-value with regard to the analysis of the 7-point ordinal scale will be calculated based on the Wald test for the difference in raw mean scores from a Cochran-Mantel-Haenszel test stratified by baseline clinical status (5 or 6) using modified ridit scores.

The common difference in mortality rates (nangibotide ACM28 rate - placebo ACM28 rate) will be estimated from a Mantel Haenszel-based analysis stratified by baseline status (5 or 6).

- If the one-sided p-value (7-point ordinal scale based analysis mentioned above) is > 0.459 (along with an odds ratio > 1 as estimated in the ordinal logistic regression)

  AND

- The estimated absolute difference in ACM28 rates (nangibotide ACM28 rate - placebo ACM28 rate) > -2%

THEN

The trial is considered futile.

8.5.2. Second interim analysis

Futility rules for claiming futility at the second interim analysis (at ~ 250 patients) are as follows:

The interim one-sided p-value with regard to the analysis of the 7-point ordinal scale will be calculated based on the Wald test for the difference in raw mean scores from a Cochran-Mantel-Haenszel test stratified by baseline clinical status (5 or 6) using modified ridit scores.

The common difference in mortality rates (nangibotide ACM28 rate - placebo ACM28 rate) will be estimated from a Mantel Haenszel-based analysis stratified by baseline status (5 or 6).

- If the one-sided p-value (7-point ordinal scale based analysis mentioned above) is > 0.126 (along with an odds ratio > 1 as estimated in the ordinal logistic regression)

  AND

- The absolute difference in ACM28 rates > -2%

THEN

The trial is considered futile.

8.5.3. Third interim analysis/End of part 2 analysis

The third interim analysis will be further detailed in a separate IA SAP.

9. Safety Analysis

Safety assessments include hematology, coagulation, plasma biochemistry, adverse events (AEs), serious adverse events (SAEs) and deaths.

All safety measurements will use all available data for analyses, including data from unscheduled visits. All safety analyses will be based on the Safety Set as defined in Section 4.4.1.

9.1. Adverse Events

An AE is any untoward medical occurrence in a patient or clinical study subject administered a medicinal product and which does not necessarily have a causal relationship with the treatment. An AE can therefore be any unfavorable and unintended sign (including an abnormal laboratory finding), symptom of disease temporarily associated with the use of an investigational medicinal product (IMP), whether or not considered related to the product.

AEs that occur prior to the first administration will be considered part of baseline information in the evaluation of study data. An Adverse Reaction (AR) is any untoward and unintended response to an IMP dose administered.

AEs experienced by the patients will be collected throughout the entire study and will be coded using the MedDRA version 24.0 or higher.

All AEs will be listed in detail.

9.1.1. Treatment Emergent Adverse Event

A treatment-emergent AE (TEAE) is defined as an AE that meets any of the following conditions:

- begins on or after the first dose of study drug;
- begins before the first dose of study drug and worsens in severity on or after the first dose of study drug;
- is completely missing an onset date and end date;
- is completely missing an onset date and the end date is on or after the first dose of study drug.

Imputed AE data will be summarized but all the original collected AE data will be presented in a listing. TEAEs will be flagged (*) in all the AE listings and will be summarized.

In the case of missing or partially missing AE onset dates, the rules described in Section 4.1 will be applied.

9.1.2. Incidence of adverse events

Summaries of the total number of TEAEs and the number and percentage of patients with at least one TEAE will be provided by treatment group. The number and percentage of patients and the number of events will also be presented by SOC and PT. At each level of patient summarization, a patient is counted once if the patient reported one or more events. Percentages will be calculated out of the number of patients in the Safety Set. The number of events at each level of SOC and PT will also be summarized.

The summary of TEAEs will be presented in descending order from the SOC with the highest total incidence (that is, summed across all treatment groups) to the SOC with the lowest total incidence. If the total incidence for any two or more SOCs is equal, the SOCs will be presented in alphabetical order. Within each SOC, the PTs will be presented in alphabetical order.

All adverse events will be listed including relationship and flag for treatment emergent ones.

9.1.3. Relationship of adverse events to study drug

The investigator will provide an assessment of the relationship of the event to the study drug. The possible relationships are “Unrelated”, “Unlikely”, “Possible” and “Probable”. "Unrelated”, “Unlikely” AEs will be categorized as “Not Related”. All other AEs: Possible”,

"Probable" will be categorized as "Related". TEAEs that are missing a relationship will be presented in the summary table as "Related" but will be presented in the data listing with a missing relationship. If a patient reports multiple occurrences of the same TEAE, only the most closely related occurrence will be presented.

9.1.4. Serious Adverse Events

The seriousness of an AE should be assessed by the investigator independently from the severity of the AE. A serious AE (SAE) is any untoward medical occurrence that at any dose results in death, is life threatening, requires inpatient hospitalization or prolongation of existing hospitalization, results in persistent or significant disability or incapacity, is a congenital anomaly or birth defect, or is an important medical event.

Other events to be treated as SAEs by the investigator are:

- **Exposure to drug during pregnancy:** In principle, pregnancy and the lactation period are exclusion criteria for clinical studies involving investigational drugs, which are not directly related to the respective conditions. In the event of a pregnancy occurring during the course of this particular study, the patient should be withdrawn from study, but closely followed-up during the entire course of the pregnancy and postpartum period.

For missing dates, the rules stated in Section Section 4.1 will be followed. If the imputed start date is prior to the AE start date, then the SAE start date should be imputed as the AE start date.

All serious adverse events will be listed including relationship and flag for treatment emergent ones.

9.1.4.1. Serious Adverse Events, related to study drug

The Serious TEAE data will be categorized and presented by SOC, PT, and relationship (i.e. "Related" and "Not Related") in a manner similar to that described in Section 9.1.2.

9.1.5. Adverse events leading to treatment discontinuation

A summary of TEAEs with a study drug action taken of "Product Discontinued" will be presented in a table in a similar manner to that described in Section 9.1.2.

9.1.6. Death

A summary of TEAEs where the answer to "Outcome" is "Fatal" will be presented in a table in a similar manner to that described in Section 9.1.2.

All patients who have an AE with an outcome of "Fatal" will be presented in a listing.

9.1.7. Overview summary

An overview summary of the number and percentage of patients with any TEAE, serious TEAE, study drug-related TEAE, study drug-related serious TEAE, TEAE leading to treatment discontinuation, TEAE leading to study discontinuation, and AE leading to death will be provided by treatment group.

9.2. Clinical laboratory evaluations

Laboratory assessments such as plasma biochemistry, hematology and coagulation will be obtained from local laboratories on the visit days specified in Section 11.1.

Summary tables presenting observed values and changes from baseline at each scheduled visit will be presented for numerical clinical laboratory tests by treatment group and overall. Summary box plots of observed values will be presented for numeric clinical laboratory results, by treatment group, including mean value over time. This will be for patients in the Safety Set.

If multiple values are collected within a post- baseline visit, the first value will be used in summary tables and figures. All values will be listed.

9.2.1. Hematology

The following hematology parameters will be summarized: hemoglobin, hematocrit, leucocytes, basophils, eosinophils, neutrophils, lymphocytes, monocytes and platelets count.

9.2.2. Plasma biochemistry

The following plasma biochemistry parameters will be summarized: aspartate aminotransferase, alanine aminotransferase, alkaline phosphatase, total bilirubin, creatinine, urea, glucose, sodium, potassium, chloride, calcium, inorganic phosphate, total protein, albumin, lactate, C Reactive Protein (CRP) and Ferritin.

9.2.3. Coagulation

The following coagulation parameters will be summarized: International Normalized Ratio (INR) and D Dimer.

9.2.4. Pregnancy test

Serum or urine pregnancy test will be performed in all female patients of childbearing potential at the Screening visit and will be listed for patients in the Safety Set.

9.3. Secondary Infection and Infection Type

Secondary infection and infection type assessments include site of infection, type of infection (Bacterial/Viral/Fungal/Combined), bacterial results (Positive/Negative) and antibiotics taken (Yes/No). Number and percentages will be calculated for patients in the Safety Set within the subgroup category. The number and percentage of patients with at least one secondary infection during the study, the number of infections per patient for patients

with at least one infection, the site of infection and the Type of infection will be summarized by treatment group and overall.

All data will be listed.

9.4. Co-administered Immunomodulatory Medications, Deaths, SAEs and Secondary Infections

At each part 2 analysis, the interaction between co-administered immunomodulatory medications and study drug will be reviewed by the DMC with data regarding the incidence of secondary infection, adverse events and death categorized by study drug and co-administered medication.

For the subgroup of subjects who died, experienced SAE or secondary infections, a listing of presence/absence, start/end date and name of co-administered immunomodulatory medications will be produced by treatment group including presence/absence, start/end date of the event.

10. References

ICH E9 Statistical Principles for Clinical Trials. https://www.ema.europa.eu/en/documents/scientific-guideline/ich-e-9-statistical-principles-clinical-trials-step-5_en.pdf

Cui, Lu., Hung, H.M. James. and Wang, Sue-Jane. (1999). Modification of sample size in groups sequential clinical trials. Biometrics. 55, 853-857

11. Appendices

11.1. Schedule of Study Procedures

	**SCR**						**EoT**			**EoS[11]**	**FU[12] Blinded**
**Assessment / Day[1]**	**≤48H**	**1**	**2**	**3**	**4**	**5**	**6**	**8 +2d**	**14 +4d**	**28[11] +7d**	**60 +7d**
Informed consent[2]	X[2]										
Inclusion/exclusion criteria	X										
Medical history and demographics	X										
Pregnancy test[3]	X										
Height and weight[4]	X										
Physical Examination	X										
Randomization	X										
Study Drug Infusion[5]		X	X	X	X	X	X				
Prior[6] and concomitant therapies	X	X	X	X	X	X	X	X	X	X	X[13]
Oxygenation and ventilation	X	X[10]	X	X	X	X	X	X	X	X	
Clinical Status[7]		X[10]	X	X	X	X	X	X	X	X	X
SOFA score		X[10]								X	
Thromboembolic events[7]		X[10]	X	X	X	X	X	X	X	X	
Secondary infections[7]		X[10]	X	X	X	X	X	X	X	X	
Adverse events[8]		X	X	X	X	X	X	X	X	X	X[14]
Hemodynamic support (vasopressor)	X	X[10]	X	X	X	X	X	X	X	X	
Renal replacement Therapy	X	X[10]	X	X	X	X	X	X	X	X	
Mortality		X	X	X	X	X	X	X	X	X	X
Clinical Laboratory (see table 2)	X	X[10]	X	X	X	X	X	X	X	X	
Procalcitonin (PCT) (see table 2)		X[10]			X		X	X	X	X	
sTREM-1 (see table 2)		X[10]			X		X	X	X	X	
IL-6 (see table 2)		X[10]			X		X	X	X	X	

Blood sample for retention (see table 2)		X[10]			X		X	X	X	X	
Discharge letter collection[9]										X	X
Quality of Life/Functional assessment											X
SCR : screening ; EoT: end of treatment; EoS : end of study											

1: Days refer to calendar days, day 1 is defined as the calendar day of first study drug administration
2: In case of emergency consent, confirmation of consent as soon as patient is capable
3: Women of childbearing potential
4: Estimate in case measurement is not feasible
5: Patients will be treated for 5 days (120 hours) or until ICU discharge, whichever is sooner
6: All medication taken within the last 24 hours before start of treatment should be documented. This does include any other study medication from other trials.
7: Assessments will be performed before IMP initiation (Day 1) thendaily until day 14. Further assessment will be made on day 28 and day 60 even in case patient is discharged from ICU.
8: AEs will be collected until day 28
9: Discharge letters from ICU discharge (where permissible and possible) at day 28 and day 60
10: Before first treatment administration
11: Day 28 and Day 60 visit may be completed by site via telephone call if visit to study center not possible.
12: By telephone or in person (Patients in part 2 or 3 only)
13: Review only of medications reported as ongoing at day 28
14: Drug related SAEs only after day 28

11.2. Interim List of Outputs for First and Second Interim Analysis

Tables
Table 1 Clinical status 7-point scale at Day 28 – mITT Set
Table 2 All-Cause Mortality Rate and Differences in Rates at Day 28 mITT Set
Table 3 Summary Table for Futility Analysis mITT Set
Table 4 Sensitivity Analysis 1 of Clinical status 7-point scale at Day 28 using Logistic Regression – mITT Set
Table 5 Sensitivity Analysis 2 of Clinical status 7-point scale at Day 28 dichotomized in six binary endpoints – mITT Set
Table 6 Sensitivity Analysis 3 of Clinical status 7-point scale at Day 28 by Randomization Stage – mITT Set
Table 7 Sensitivity Analysis 4 of Clinical status 7-point scale at Day 28 Categorized as Improved/Not Improved from Baseline – mITT Set
Table 8 Sensitivity Analysis 5 of Clinical status 7-point scale at Day 28 Categorized as Improved/Unchanged/Worsened from Baseline – mITT Set
Table 9 Subgroup Analysis 1 of Clinical status 7-point scale at Day 28 Categorized as Improved/Not Improved from Baseline by Baseline sTREM-1 level– mITT Set
Table 10 Subgroup Analysis 2 of Clinical status 7-point scale at Day 28 Categorized as Improved/Unchanged/ Worsened from Baseline by Baseline sTREM-1 level– mITT Set
Table 11 Sensitivity Analysis 1 of All-Cause Mortality Rate and Differences in Rates at Day 28 by Randomization Stage - mITT Set
Table 12 Sensitivity Analysis 2 of All-Cause Mortality at Day 28 using Kaplan-Meier Analysis - mITT Set
Table 13 Sensitivity Analysis 3 of All-Cause Mortality Rate and Differences in Rates at Day 60 - ACM60 mITT Set
Table 14 Sensitivity Analysis 4 of All-Cause Mortality Rate and Differences in Rates at Day 60 by Randomization Stage – ACM60 mITT Set
Table 15 Sensitivity Analysis 5 of All-Cause Mortality at Day 60 using Kaplan-Meier Analysis – ACM60 mITT Set
Table 16 Subgroup Analysis 1 of All-Cause Mortality Rate and Differences in Rates at Day 28 by Baseline sTREM-1 level– mITT Set
Table 17 Subgroup Analysis 2 of All-Cause Mortality Rate and Differences in Rates at Day 60 by Baseline sTREM-1 level – ACM60 mITT Set
Table 18 Number of Days Free of Organ Support at Day 28, Overall and by Baseline Clinical Status– mITT set
Table 19 Ventilation Type at Day 28, Overall and by Baseline Clinical Status– mITT set
Table 20 Disposition - All Enrolled Set

**Tables**
Table 21 Demographics - Safety Set
Table 22 Baseline Disease Characteristics – Ventilation Type and Clinical Status - Safety Set
Table 23 Prior Medications for Covid-19 - Safety Set
Table 24 Concomitant Medications for Covid-19 - Safety Set
Table 25 Study Drug Infusion - Safety Set
Table 26 Summary of Adverse Events - Safety Set
Table 27 Treatment-Emergent Adverse Events by System Organ Class and Preferred Term - Safety Set
Table 28 Treatment-Emergent Serious Adverse Events by Relationship to Study Drug, System Organ Class and Preferred Term - Safety Set
Table 29 Treatment-Emergent Adverse Events with Outcome of Death by System Organ Class and Preferred Term - Safety Set
Table 30 Treatment-Emergent Adverse Events Leading to Treatment Discontinuation by System Organ Class and Preferred Term Safety Set
Table 31 Summary of Hematology by Visit - Safety Set
Table 32 Summary of Plasma Biochemistry by Visit - Safety Set
Table 33 Summary of Coagulation Parameters by Visit - Safety Set
Table 34 Summary of Secondary Infections - Safety Set

**Listings**
Listing 1 Disposition - All Enrolled Set
Listing 2 Demographics - Safety Set
Listing 3 Medical History – Safety Set
Listing 4 Prior and Concomitant Medications for Covid-19 - Safety Set
Listing 5 Study Drug Infusion - Safety Set
Listing 6 All-Cause Mortality - mITT Set
Listing 7 Clinical Status - mITT Set
Listing 8 Ventilation - mITT Set
Listing 9 Renal Replacement Therapy - mITT Set
Listing 10 Vasopressor Use - mITT Set
Listing 11 Adverse Events - Safety set
Listing 12 Serious Adverse Events - Safety Set
Listing 13 Adverse Events Resulting in Death - Safety Set
Listing 14 Hematology - Safety Set
Listing 15 Plasma Biochemistry - Safety Set
Listing 16 Coagulation - Safety Set

Listings
Listing 17 Secondary Infections - Safety Set
Listing 18 Co-administered Immunomodulatory Medications and Selected Events - Safety Set

Figures
Figure 1 Boxplots of Hematology - Safety Set
Figure 2 Boxplots of Biochemistry - Safety Set
Figure 3 Boxplots of Coagulation - Safety Set
