# Supplementary material for: Evaluation of the efficacy and safety of TREM-1 inhibition with nangibotide in patients with COVID-19 receiving respiratory support: the ESSENTIAL randomised, double-blind trial
Source: eClinicalMedicine. 2023 May 31;60:102013. doi: 10.1016/j.eclinm.2023.102013 (PMC10231876; doi:10.1016/j.eclinm.2023.102013)
Supplement: ESM5 INOMOTC204 Final SAP [file mmc4.pdf]

INOTREM

MOT-C-204 ESSENTIAL

Exploratory study of the safety, tolerability and efficacy of nangibotide in patients with COVID-19 receiving ventilatory support and features of systemic inflammation

A randomized, double-blind, placebo-controlled study with adaptive features

02AUG2022

Statistical Analysis plan of study - early termination

Version 1.0

Prepared by:

PPD
Granta Park, Great Abington,
Cambridge, CB21 6GQ

TABLE OF CONTENTS

LIST OF ABBREVIATIONS IV

1. INTRODUCTION 1

2. OBJECTIVES 1

2.1. PRIMARY 1
2.2. SECONDARY 1

3. INVESTIGATIONAL PLAN 2

3.1. DATA MONITORING COMMITTEE 2
3.2. TREATMENTS 2
3.3. STUDY ENDPOINTS 2
3.3.1. Primary and Key secondary endpoints 2
3.3.2. Other Secondary efficacy and safety endpoints 3

4. GENERAL STATISTICAL CONSIDERATIONS 3

4.1. TABULATIONS AND PRESENTATIONS 3
4.2. SAMPLE SIZE 5
4.3. RANDOMIZATION, STRATIFICATION, AND BLINDING 6
4.3.1. Randomization 6
4.3.2. Stratification 6
4.3.3. Blinding 6
4.4. ANALYSIS SET 6
4.4.1. Safety Set 6
4.4.2. All Randomized Patients (Intent-to-Treat (ITT)) Set 6
4.4.3. Modified Intent-to-Treat (mITT) Set 7
4.4.4. Per Protocol (PP) Set 7

5. PATIENT DISPOSITION 7

5.1. DISPOSITION 7

6. DEMOGRAPHICS AND BASELINE CHARACTERISTICS 7

6.1. DEMOGRAPHICS 7
6.2. BASELINE DISEASE CHARACTERISTICS 8
6.3. MEDICAL HISTORY 8

7. TREATMENTS AND MEDICATIONS 8

7.1. PRIOR AND CONCOMITANT MEDICATIONS 8
7.1.1. Prior medications related to COVID-19 9
***7.1.2. Concomitant medications related to COVID-19** 9*
7.2. EXTENT OF EXPOSURE 9

8. EFFICACY ANALYSES 10

8.1. ANALYSIS OF THE PRIMARY ENDPOINT OF PART 2: CLINICAL STATUS 10
8.2. ANALYSIS OF MORTALITY AT DAY 28 (ACM28) 12
8.3. EXPLORATORY EFFICACY ANALYSES 13

8.3.1. Days free of organ support (respiratory, renal and vasopressor) ... *13*
8.3.2. Respiratory support type ... *13*
8.3.3. Hospital Free Survival at day 28 ... *14*
8.3.4. Sustained remission ... *14*
8.3.5. Alive Free of Respiratory Support at Day 28 and at Day 60 ... *14*
8.4. EVALUATION OF THE OPTIMAL TREATMENT POPULATION ... 15
9. SAFETY ANALYSIS ... 15
9.1. ADVERSE EVENTS ... 15
9.1.1. Treatment Emergent Adverse Event ... *15*
9.1.2. Incidence of adverse events ... *16*
9.1.3. Relationship of adverse events to study drug ... *16*
9.1.4. Serious Adverse Events ... *16*
9.1.4.1. Serious Adverse Events, related to study drug ... *17*
9.1.5. Adverse events leading to treatment discontinuation ... *17*
9.1.6. Death ... *17*
9.1.7. Overview summary ... *17*
9.2. CLINICAL LABORATORY EVALUATIONS ... 17
9.2.1. Hematology ... *18*
9.2.2. Plasma biochemistry ... *18*
9.2.3. Coagulation ... *18*
9.2.4. Pregnancy test ... *18*
9.3. SECONDARY INFECTION AND INFECTION TYPE ... 18
9.4. CO-ADMINISTERED IMMUNOMODULATORY MEDICATIONS, DEATHS, SAES AND SECONDARY INFECTIONS ... 18
10. REFERENCES ... 19
11. APPENDICES ... 20
11.1. SCHEDULE OF STUDY PROCEDURES ... 20

List of Abbreviations

AE	Adverse Event
AR	Adverse Reaction
ATC	Anatomical Therapeutic Chemical
CI	Confidence Interval
COVID-19	Coronavirus disease 2019
CoV	Coronavirus
CRF	Case Report Form
CRP	C Reactive Protein
d/D	Day
DMC	Data Monitoring Committee
ECMO	Extracorporeal Membrane Oxygenation
EoS	End of Study
EoT	End of Treatment
FU	Follow-up
h/H	Hour
IA	Interim Analysis
ICH	International Council for Harmonisation of Technical Requirements for Pharmaceuticals for Human Use
ICU	Intensive Care Unit
IL-6	Interleukin-6
IMV	Invasive Mechanical Ventilation
IMP	Investigational Medicinal Product
INR	International Normalized Ratio
IQR	Interquartile Range
ITT	Intent-to-Treat
MedDRA	Medical Dictionary for Regulatory Activities
MH	Mantel-Haenszel
MITT	Modified Intent-to-Treat
PCT	Procalcitonin
PD	Pharmacodynamic
PP	Per Protocol
PT	Preferred Term
SAE	Serious Adverse Event
SAP	Statistical Analysis Plan
SD	Standard Deviation
SOC	System Organ Class
SOFA	Sequential Organ Failure Assessment
sTREM-1	Soluble TREM-1
TLFs	Tables, Listing and Figures
TEAE	Treatment-Emergent Adverse Events
TREM-1	Triggering Receptor Expressed on Myeloid Cells
WHODRUG	World Health Organization Drug Dictionary

1. Introduction

This study with an adaptive design was initially designed to assess safety and tolerability of nangibotide in patients with COVID-19 receiving ventilatory support and features of systemic inflammation with a sample size of 60 patients. An extension of the study that will also allow for appropriate assessment of efficacy was approved through protocol amendment 5 and after review of safety and tolerability on the 60 first patients that form the part 1 of the study.

According to protocol amendment 7.0 the study was divided into three parts:

Part 1: A safety and tolerability study has completed recruitment of 60 patients. An independent Data Monitoring Committee (DMC) conducted an analysis of safety and tolerability and recommends continuation of part 2.
Part 2: Recruitment of 310 new patients including two interim futility analyses and one end of part 2 analysis for progression to part 3 or cessation due to futility.
Part 3: Recruitment of at least a further 360 patients (total 730) to assess the impact of study drug on mortality at Day 28 although this sample size may be increased based upon DMC recommendation.

Due primarily to a low enrolment rate as a consequence of the low proportion of eligible COVID-19 patients requiring admission to intensive care, the study has been terminated early with a sample size of 220 patients

This statistical analysis plan (SAP) describes the planned analyses for the MOT-C-204 study that was terminated early. Details on the study design can be found in the current version of the study protocol Version 8.0, dated 01JUNE2022. This statistical analysis plan is based on International Council for Harmonization of Technical Requirements for Pharmaceuticals for Human Use (ICH) E3 and E9 Guidelines.

2. Objectives

The full study objectives are as follows:

2.1. Primary

- To evaluate the impact of nangibotide on the progression of disease in patients receiving ventilatory support due to COVID-19

2.2. Secondary

Secondary objectives of the study are to investigate the safety and efficacy of nangibotide in patients receiving ventilatory support due to COVID-19.

This includes:
• To evaluate the effects of nangibotide exposure over 5 days in patients with COVID-19
• To evaluate the effect of nangibotide on clinical parameters
• To evaluate the safety and tolerability of nangibotide in patients with COVID-19
• To evaluate PD relationship to TREM-1 pathway related markers
• To evaluate the natural history of the disease regarding the activation of the TREM-1 pathway measured by soluble TREM-1 plasma concentrations over time
over time

Next sections of this document more specifically focus on part 2 of the study.

3. Investigational Plan

3.1. Data Monitoring Committee

In part 2, the DMC monitored the safety of the MOT-C-204 studied and assessed the study for potential futility at the following scheduled timepoint:

Timepoint	Assessment
130 (60 from part 1 and 70 from part 2) patients have completed the 28-day period after randomization.	Safety, Efficacy futility on clinical status 7-point ordinal scale and mortality at Day 28.

An independent SAP dated on the 14DEC2021 described the analyses performed for this interim analysis. The first of interim analyses assessing the safety and efficacy of nangibotide in the first 130 patients in terms of clinical status and mortality. The DMC recommended continuation of the study without modification.

3.2. Treatments

In all parts of the study, the intervention will remain the same.
Patients will receive a continuous intravenous (i.v.) infusion of nangibotide at 1.0 mg/kg/h or a matching placebo. Treatment with study drug must be initiated as early as possible but no later than 48 hours after the initiation of ventilatory support. Patients will be treated for 5 days or until discharge from critical care, whichever is sooner. The treatment with study drug will be in addition to standard of care. Follow-up visits will be performed on days 8 and 14. The end of study visit is at Day 28. A further follow up visit will be undertaken on day 60.

3.3. Study Endpoints

3.3.1. Primary and Key secondary endpoints

- Primary endpoint of part 2: Clinical status 7-point ordinal scale at Day 28
- Key secondary endpoint of part 2: All-cause mortality at Day 28

3.3.2. Other Secondary efficacy and safety endpoints

- Clinical status as improvement from baseline (Yes vs No) at Day 28 and Day 60
- Clinical status as a 3-point ordinal scale (change from baseline categorized as improved, unchanged, worsened) at Day 28 and Day 60
- All-cause mortality on day 28 and day 60
- Days free of organ support (respiratory, renal and vasopressor) at Day 28
- Respiratory support type at Day 28 and Day 60
- Safety laboratory tests (as part of routine clinical care): hematology, coagulation, plasma biochemistry
- Adverse events (AEs), serious adverse events (SAEs) and deaths
- Suspected adverse drug reactions (serious and non-serious)
- Secondary infections
- Hospital free survival (the proportion of patients alive and not hospitalized on day 28)
- Sustained improvement (The proportion of patients alive and not hospitalized on day 60)
- Alive and free of respiratory support at Day 28 and Day 60

4. General Statistical Considerations

4.1. Tabulations and Presentations

All summaries and results in Tables, Listings and Figures (TLFs) should be presented for the following treatment groups:

Treatment groups	Label in TLFs
1.0 mg/kg/h continuous intravenous (i.v.) infusion of nangibotide	Nangibotide 1.0 mg/kg/h
Matching placebo	Placebo

Continuous data will be described using descriptive statistics (i.e. n, mean, standard deviation, median, interquartile range (IQR), minimum, and maximum). Categorical data will be described using the patient count and percentage in each category. For the summary statistics of all numerical variables unless otherwise specified, minimum and maximum will be displayed to the same level of precision as reported. Mean, median and IQR will be displayed to one level of precision greater than the data collected. Standard deviation will be displayed to two levels of precision greater than the data collected.

When count data are presented, the percentage will be suppressed when the count is zero in order to draw attention to the non-zero counts. A row denoted “Missing” will be included in count tabulations where specified on the shells to account for dropouts and missing values. The denominator for all percentages will be the number of patients in that treatment within

the Analysis Set of interest, unless otherwise specified. Percentages will be presented to one decimal place.

Any imputed data will be used in summary tables and figures and the original collected data will be displayed in listings. Data will be displayed in all listings sorted by treatment group. Patients will be identified in the listings by the patient identification number concatenated with the investigator number.

Two-sided p-values will be displayed except for the primary analysis of clinical status (7-point ordinal scale) at Day 28.

Due to the exploratory nature of this prematurely stopped trial, an adjusted alpha nominal level is proposed for the test of the primary endpoint to take into account the early stop and control the type 1 error at its usual 1-sided level of 0.025. A **conservative** alpha spending function (i.e. the Lan DeMets Obrien-Fleming spending function) is proposed blind to control the type I error: the nominal 1-sided alpha level considered is 0.003303, derived from the Lan DeMets Obrien-Fleming spending function at 220 patients (i.e. ~210 patients incorporating the loss of efficiency due to the randomization imbalance in part 1). The choice of this alpha spending function (i.e. the Lan DeMets Obrien-Fleming spending function) and therefore of a low alpha nominal level of 0.003303 disfavours the rejection of the null hypothesis and can be considered as reasonably conservative compared to the very stringent and highly conservative nominal alpha level of Haybittle Peto (i.e., 0.0001). There is one primary endpoint clearly defined. No adjustment for multiple endpoints will be performed as other endpoints in this prematurely stopped trial are considered as exploratory.

Other p-values derived on secondary endpoints are considered as exploratory only.

Tipping point analyses and multiple imputation approaches may be used as post-hoc analyses if relevant.

Subgroups analyses based on patients baseline sTREM-1 level (above or equal to the median vs below) described in this SAP will be based on calculation performed on the Safety Set.

All analyses will be conducted using SAS Version 9.4 or higher.

Baseline and Study Day

Unless otherwise specified, baseline (day 1) will be defined as the last non-missing evaluation prior to or on the date that the first dose of treatment is given.
For visits prior to day of first study dose, study day will be calculated as:
assessment date – first study dose date.
For visits on or after the day of first study dose, the study day will be calculated as:
assessment date - first study dose date + 1.

Visit Windows

All data summarized by visit will be based on the visit name collected on the electronic Case Report Form (eCRF) page. For data from unscheduled visits, these will be listed but not included in any by-visit summaries or analyses.

Missing Start and Stop Dates

For the purpose of inclusion in prior and/or concomitant medication and AE tables, incomplete medication start and stop dates will be imputed as follows:

Missing start dates (where UN, UNK and UNKN indicate unknown or missing day, month and year respectively):

- UN-MMM-YYYY: If the month and year are different from the month and year of the first dose of study drug, assume 01-MMM-YYYY. If the month and year are the same as the first dose of study drug month and year and the end date (after any imputation) is on or after the first dose of study drug, then assume the date of the first dose of study drug. If the month and year are the same as the first dose of study drug month and year and the end date (after any imputation) is prior to the first dose of study drug, then assume the end date for the start date;
- DD-UNK-YYYY/UN-UNK-YYYY: If the year is different from the year of the first dose of study drug, assume 01-JAN-YYYY of the collected year. If the year is the same as the first dose of study drug year and the end date (after any imputation) is on or after the first dose of study drug, then assume the date of the first dose of study drug. If the year is the same as the first dose of study drug and the end date (after any imputation) is prior to the first dose of study drug, then assume the end date for the start date.

Missing stop dates (where UN, UNK and UNKN indicate unknown or missing day, month and year respectively):

- UN-MMM-YYYY: Assume the last day of the month;
- DD-UNK-YYYY/UN-UNK-YYYY: Assume 31-DEC-YYYY.

In case of the death of the patients and the imputed end date is after the date of death, the stop/end date will be imputed as the date of death.

4.2. Sample Size

A total of 370 patients were planned to be enrolled at end of part 2.
Due primarily to a low enrolment rate as a consequence of the low proportion of eligible COVID-19 patients requiring admission to intensive care, the study was terminated early with a sample size of 220 patients.

Safety, Efficacy futility on clinical status 7-point ordinal scale and mortality at Day 28 were analyzed by the DMC after 130 (60 from part 1 and 70 from part 2) patients had completed the 28-day period after randomization (refer to DMC SAP).

4.3. Randomization, Stratification, and Blinding

4.3.1. Randomization

The randomization scheme will be generated by an independent statistician who is not part of the study team. A randomization number will be assigned to each patient. The randomization scheme will be generated by a block randomization scheme.

In stage one part 1 of the study, eligible patients were randomized in a 1:1 ratio into 1 of the 2 treatment arms (20 first patients). In stage 2 part 1 of the study, patients were allocated to nangibotide treatment or placebo in a 3:1 ratio resulting in 20 subjects to be randomized on Placebo and 40 subjects randomized on nangibotide at the end of part 1. In part 2 eligible patients will be randomized in a 1:1 ratio into 1 of the 2 treatment arms.

4.3.2. Stratification

The randomization will be stratified according to site.

4.3.3. Blinding

The study will be conducted in a double-blind fashion, whereby patients, clinical study site staff as well as sponsor and CRO personnel involved in the monitoring and evaluation of the study are blinded to study drug assignment.

Study drug and matching placebo will be provided as individually numbered vials in secondary packaging of identical appearance.

Upon completion of the study, after the database lock and after the blind is revealed, the randomization list will be filed in the Trial Master File.

4.4. Analysis Set

The Safety analyses will be based on the Safety Set and the Efficacy analyses will be based on mITT Set. Main efficacy analyses may be repeated on the ITT set.

4.4.1. Safety Set

All enrolled patients having received at least one dose of the prescription of study medication (either nangibotide or placebo). The Safety Set will be the basis for safety analyses. Patients will be assigned to the treatment group as treated for the Safety Set. Patients receiving at least one dose of nangibotide will be reported in the nangibotide group.

4.4.2. All Randomized Patients (Intent-to-Treat (ITT)) Set

ITT set will include all patients who are randomized into the study and considered as randomized, i.e. with study drug assignments designated according to initial randomization, regardless of whether patients received what was assigned. Main efficacy analyses may be repeated on the ITT set but the primary analysis is on mITT set defined below.

4.4.3. Modified Intent-to-Treat (mITT) Set

All randomized patients having received at least one dose of the prescription of study drug (either nangibotide or placebo). Patients will be assigned to the treatment group as randomized (i.e. with study drug assignments designated according to initial randomization, regardless of whether patients received what was assigned). The mITT Set will be considered as the primary set for interim and final efficacy analyses.

4.4.4. Per Protocol (PP) Set

All mITT patients without any major protocol deviation. Major protocol deviations will be identified during data review meetings that will be held before unblinding and database lock. The PP Set will be considered as support analysis set for primary and key secondary endpoint in final efficacy analyses.

5. Patient Disposition

5.1. Disposition

Patient disposition will be summarized using the All Enrolled Set. The total number of patients will be summarized for the following categories: those who were screened, and those who were not randomized. The number and percentage of patients within each treatment group will be presented by the following categories: randomized, received study drug, did not receive study drug, completed study drug, discontinued study drug (and reason), completed study, and withdrawn from study (and reason). Percentages will be based on the number of patients randomized. Disposition data will be presented as well according to the baseline sTREM-1 level (above or equal to the median vs below).

Patient disposition data will be presented in a listing.

6. Demographics and Baseline Characteristics

6.1. Demographics

Demography data: age (years), sex and ethnic factors will be summarized by treatment group and overall for the Safety Set. Demography data will be presented as well according to the baseline sTREM-1 level (above or equal to the median vs below).

The following baseline characteristics will be summarized for the same analysis set:

- Patient characteristics: baseline height (cm), baseline weight (kg) and BMI (kg/m^2). Fertility status will also be presented for female patients.

Age, baseline height, baseline weight and BMI will be summarized using descriptive statistics. The number and percentage of patients by sex (Male, Female), ethnic factors (White or Caucasian, Black or African American, Asian, Hispanic, Latino or of Spanish Origin, American Indian or Alaska Native, Native Hawaiian or Other Pacific Islander, and

Other Ethnic Factors) and fertility status (Childbearing potential; Yes, No, Unknown and Fertility Status; Sterile, Post-Menopausal, Potentially Able to Bear Children), will also be reported. Percentages will be based the total number of patients in the Safety set.

This data will be listed for the Safety Set.

6.2. Baseline Disease Characteristics

The type of ventilation the patients are on at baseline and whether this is invasive mechanical ventilation or not, the baseline clinical status and the baseline sTREM-1 level (above or equal to the median vs below) will be presented by treatment group and overall for the Safety set. Baseline characteristics data will be presented as well according to the baseline sTREM-1 level (above or equal to the median vs below).

The following categories of mechanical ventilation are considered invasive:

- Ventilation requiring intubation
- ECMO
- Extracorporeal CO2 clearance

The following categories of mechanical ventilation are considered non-invasive:

- Non-invasive ventilation
- High flow

6.3. Medical History

Medical history will be coded using Medical Dictionary for Regulatory Activities (MedDRA) version 23.0 or higher. The number and percentage of patients with medical history will be summarized overall and by System Organ Class (SOC) and Preferred Term (PT) for patients in the Safety Set. Medical history will be listed for the Safety Set.

7. Treatments and Medications

7.1. Prior and Concomitant Medications

All medications will be coded according to the World Health Organization drug dictionary (WHODRUG) March 2020, or higher.

A prior medication is defined as any medication with a start date that is prior to the date of the first study drug dose.

A concomitant medication is defined as any medication that has a start and stop date that is on or after the first study drug dose date or that has a start date prior to the first study drug dose date but an end date that is after the first study drug dose date.

For the purpose of inclusion in prior and/or concomitant medication tables, incomplete medication start and stop dates will be imputed as described in Section 4.1.

If start date is completely missing and end date is after first dose intake or completely missing, then the medication will be classified as both prior and concomitant. If the start date is completely missing and the end date is prior to the first dose of study drug, then the medication will be classified as prior. If the end date is missing and the start date is after first dose intake, then the medication will be considered as ongoing and classified as concomitant. If the end date is missing and the start date is before first dose intake, then the medication will be considered as ongoing and classified as both prior and concomitant.

All Prior and concomitant medications and those related to COVID 19 will be presented in separate listings for the Safety Set. Therapies will be flagged as prior (P), concomitant (C) or prior and concomitant (PC).

Prior and concomitant medications related to COVID-19 will be selected based on CRF data using 'For COVID-19' item in the 'Previous and Concomitant Therapies' Form. Vasopressors collected in a specific form will be listed separately.

7.1.1. Prior medications related to COVID-19

The total number of prior medications related to COVID-19 and the number and percentages of patients with at least one prior medication related to COVID-19 will be summarized by treatment group and overall. The number and percentages of prior medications related to COVID-19 will be summarized by anatomical therapeutic chemical (ATC) classification category (level 4), preferred term, treatment group and overall for the Safety Set.

7.1.2. Concomitant medications related to COVID-19

The total number of concomitant medications related to COVID-19 and the number and percentages of patients with at least one concomitant medication related to COVID-19 will be summarized by treatment group and overall. The number and percentages of concomitant medications will be summarized by ATC classification category (level 4), preferred term, treatment group and overall for the Safety Set.

7.2. Extent of Exposure

Duration of exposure to study drug will be calculated in hours as:

Date and time of end of study drug infusion – date and time of start of study drug infusion.

Duration of exposure to study drug will be summarized by treatment group for all patients in the Safety Set.

The number and percentage of patients who had infusion interruptions will be summarized along with categorical summary of the number of interruptions for patients in the Safety Set.

All study drug information including reasons for drug interruptions will be listed for patients in the Safety Set.

Extent of Exposure data will be presented as well according to the baseline sTREM-1 level (above or equal to the median vs below).

8. Efficacy Analyses

The m-ITT set will be used for all efficacy analyses. Main efficacy analyses may be repeated on the ITT and Per Protocol analysis sets.

8.1. Analysis of the Primary Endpoint of Part 2: Clinical Status

The clinical status (7-point ordinal scale) at Day 28 will be compared between treatment group using a CMH stratified by baseline clinical status. At baseline, patients are hospitalized on invasive mechanical ventilation or ECMO (Clinical status = 6) or hospitalized, on non-invasive ventilation or high flow oxygen devices (Clinical status = 5).

Patients with a missing clinical status at Day 28 visit and who died prior to or on Day 28 will be assigned the category of 7 at Day 28. Patients with a missing clinical status at Day 28 visit and without documented death occurring prior to or on Day 28 will be assigned the last available clinical status.

The distribution (counts and percentages) of the 7-point ordinal scale will be displayed by treatment group and baseline level (5 or 6) (i.e., the 2x2x7 contingency table).

The 7-point ordinal scale will be compared between treatment groups using a Cochran-Mantel-Haenszel test stratified by baseline clinical status (5 or 6) using modified ridit scores. The Wald test one-sided p-value for the difference in raw mean scores will be provided.

The same analysis will be conducted in patients with a sTREM-1 above the median value at baseline vs below.

Sensitivity analyses:

Primary sensitivity analysis: these will be performed in overall patients and according to the baseline sTREM-1 level (above or equal to the median vs below).

The treatment effect will be estimated in an ordinal logistic regression on the 7-point ordinal scale adjusting for treatment and baseline clinical status assuming odds proportionality. The odds ratio for treatment effect with its 95% confidence interval and p-value will be estimated in this model.

As the odds proportionality may not hold (the p-value of the test for odds proportionality will be provided), the 7-point ordinal scale will be dichotomized in six binary endpoints : <= cutoff vs > cut-off: 1 vs >1, <=2 vs >2, <=3 > 3, <=4 vs >4 , <=5 vs > 5, <=6 vs 6 (of

note , the last cut-off corresponds to the analysis of mortality at day D28). For each cutoff, treatment groups will be compared by a Cochran-Mantel-Haenszel test stratified by baseline clinical status (5 or 6). Odds ratios (with their 95% confidence intervals and p-values) will also be provided. 2x2x2 contingency tables (counts and percentages) will be provided for each cut-off.

Other sensitivity analyses: these will be performed in overall patients and according to the baseline sTREM-1 level (above or equal to the median vs below) except for analyses stratified by randomization stage.

1) The 7-point ordinal scale will be compared between treatment groups using a Cochran-Mantel-Haenszel test stratified by randomization stage (part 1 stage 1, part 1 stage 2 and part 2) and baseline status (5 vs 6). The Wald test p-value for the difference in raw mean scores will be provided.

2) The change in the 7-point ordinal scale from baseline to Day 28 will be categorized in two categories: 'improved', 'not improved'.

   The distribution (counts and percentages) of the binary scale will be displayed by treatment group and baseline level (5 or 6) (i.e., the 2x2x2 contingency table).

   The treatment groups will be compared by a Cochran-Mantel-Haenszel test stratified by baseline clinical status (5 or 6). The odds ratio (with its 95% confidence interval and p-values) will also be provided.

3) The change in the 7-point ordinal scale from baseline to Day 28 will be categorized in three categories: 'improved', 'unchanged', 'worsened'.

   The distribution (counts and percentages) of the 3-point ordinal scale will be displayed by treatment group and baseline level (5 or 6) (i.e., the 2x2x3 contingency table).

   The 3-point ordinal scale will be compared between treatment groups using a Cochran-Mantel-Haenszel test stratified by baseline clinical status (5 or 6) using modified ridit scores. The Wald test p-value for the difference in raw mean scores will be provided. The treatment effect will be estimated in an ordinal logistic regression on the 3-point ordinal scale adjusting for treatment and baseline clinical status assuming odds proportionality. The odds ratio for treatment effect with its 95% confidence interval and p-value will be estimated in this model.

8.2. Analysis of Mortality at Day 28 (ACM28)

All-Cause Mortality will be assessed using survival status up to study day 31, i.e. any death occurring between the start of study drug infusion and the start of study drug infusion + 30 days.

The number and percentage of patient alive, dead and lost to follow-up at Day 28 will be summarized by treatment group. Any patient with last date known to be alive < Day 28 without death information will be considered lost to follow-up in this summary.

Last known date to be alive will be derived using the following information:

- Death date
- If death date is not documented take the maximum of the following dates:
    - 'Date last known to be alive' documented by the investigator
    - Last patient subject visit date including date of documented follow-up clinical status planned at Day 28 and Day 60
- If derived last known alive date still isn't populated the maximum of the following can be considered:
    - Date of laboratory samples
    - Date of end of organ support
    - Date of end of follow-up, only if reason is completed
    - End date of any adverse events

Inferential analysis will be based on patients which status (alive or dead) is known at Day 28 (i.e. excluding patient lost to follow-up before Day 28). Missing data will not be imputed.

Mortality rate at Day 28 will be compared between treatment group using a CMH adjusted for baseline clinical status (5 vs 6). The common difference in mortality rates between nangibotide and placebo (nangibotide ACM28 rate - placebo ACM28 rate) will be estimated with its 95% confidence interval using the Mantel-Haenszel method. Hence, a negative difference in ACM28 rates favors nangibotide.

This analysis will be done as well according to the baseline sTREM-1 level (above or equal to the median vs below).

Sensitivity analysis :

1) The mortality rate at Day 28 will be compared between treatment group using a CMH adjusted for baseline clinical status (5 vs 6) and randomization stage (part 1 stage 1, part 1 stage 2 and part 2). The adjusted difference in mortality rates between nangibotide and placebo (nangibotide ACM28 rate - placebo ACM28 rate) will be estimated with its 95% confidence interval using Mantel-Haenszel method.

2) Kaplan-Meier estimates of mortality will also be estimated at Day 28. The same analysis will be conducted in patients with a sTREM-1 above vs below the median value at baseline.

3) The same analyses mentioned above (including sensitivity analyses) will be performed at Day 60.

8.3. Exploratory Efficacy Analyses

Descriptive analyses will be provided only.

8.3.1. Days free of organ support (respiratory, renal and vasopressor)

The number of days free of organ support (respiratory, renal and vasopressor) will be calculated as the total number of calendar days from treatment start up (Day 1) to Day 28, when a patient is alive and does not require any organ support. If a patient died on or before Day 28, the number of days will be set to 0. For patients withdrawn alive before Day 28, number of days will be calculated until day of withdrawal. The mean, median, Q1 and Q3, min and max of the distribution of the number of days free of organ support will be provided by treatment group overall and by baseline clinical status.

Subgroup analyses :

Days free of organ support will be described by baseline sTREM-1 level (above or equal to the median vs below).

8.3.2. Respiratory support type

The number and percentage of patients under each kind of ventilation at Day 28 and the number and percentage of patients under invasive mechanical ventilation at Day 28 and Day 60 will be described by treatment overall and by baseline clinical status.

The following categories of mechanical ventilation are considered invasive:

- Ventilation requiring intubation
- ECMO
- Extracorporeal CO2 clearance

The following categories of mechanical ventilation are considered non-invasive:

- Non-invasive ventilation
- High flow

Patients who died or are lost to follow-up before Day 28 or before Day 60 will be excluded from these summary tables.

Subgroup analyses :

Respiratory support will be described by baseline sTREM-1 level (above or equal to the median vs below).

8.3.3. Hospital Free Survival at day 28

The number and percentage of patients alive and not hospitalized at Day 28 will be described by treatment overall and by baseline clinical status. Patients withdrawn alive before Day 28 and without any information on survival/hospitalization status on or after Day 28 will be considered as unknown.

Subgroup analyses :

Hospitalization free survival at day 28 will be described by baseline sTREM-1 level (above or equal to the median vs below).

8.3.4. Sustained remission

The number and percentage of patients alive and not hospitalized at Day 60 will be described by treatment overall and by baseline clinical status. Patients withdrawn alive before Day 60 and without any information on survival/hospitalization status on or after Day 60 will be considered as unknown.

Subgroup analyses :

Sustained remission will be described by baseline sTREM-1 level (above or equal to the median vs below).

8.3.5. Alive Free of Respiratory Support at Day 28 and at Day 60

The number and percentage of patients alive and free of any respiratory support (all ventilation types listed in section 8.3.2) at Day 28 and Day 60 will be described by treatment overall and by baseline clinical status. Patients withdrawn alive before Day 28/60 and without any information on survival/clinical status on or after Day 28/60 will be considered as unknown.

Subgroup analyses :

The number and percentage of patients alive and free of respiratory support at day 28 and Day 60 will be described by baseline sTREM-1 level (above or equal to the median vs below).

8.4. Evaluation of the optimal treatment population

The baseline sTREM-1 level and other clinical characteristics will be investigated in order to identify which patients are most likely to respond nangibotide therapy. The best cut-off value for baseline sTREM-1 as a predictive biomarker will be determined based on clinically relevant outcomes which may include but are **not** limited to clinical status, all cause mortality at day 28 and day 60, other clinical outcomes at day 28 and day 60.

9. Safety Analysis

Safety assessments include hematology, coagulation, plasma biochemistry, adverse events (AEs), serious adverse events (SAEs) and deaths.

All safety measurements will use all available data for analyses, including data from unscheduled visits. All safety analyses will be based on the Safety Set as defined in Section 4.4.1.

All safety analyses will be described by baseline sTREM-1 level (above or equal to the median vs below).

9.1. Adverse Events

An AE is any untoward medical occurrence in a patient or clinical study subject administered a medicinal product and which does not necessarily have a causal relationship with the treatment. An AE can therefore be any unfavorable and unintended sign (including an abnormal laboratory finding), symptom of disease temporarily associated with the use of an investigational medicinal product (IMP), whether or not considered related to the product. AEs that occur prior to the first administration will be considered part of baseline information in the evaluation of study data. An Adverse Reaction (AR) is any untoward and unintended response to an IMP dose administered.

AEs experienced by the patients will be collected throughout the entire study and will be coded using the MedDRA version 24.0 or higher.

All AEs will be listed in detail.

9.1.1. Treatment Emergent Adverse Event

A treatment-emergent AE (TEAE) is defined as an AE that meets any of the following conditions:

- begins on or after the first dose of study drug;
- begins before the first dose of study drug and worsens in severity on or after the first dose of study drug;
- is completely missing an onset date and end date;
- is completely missing an onset date and the end date is on or after the first dose of study drug.

Imputed AE data will be summarized but all the original collected AE data will be presented in a listing. TEAEs will be flagged (*) in all the AE listings and will be summarized.

In the case of missing or partially missing AE onset dates, the rules described in Section 4.1 will be applied.

9.1.2. Incidence of adverse events

Summaries of the total number of TEAEs and the number and percentage of patients with at least one TEAE will be provided by treatment group. The number and percentage of patients and the number of events will also be presented by SOC and PT. At each level of patient summarization, a patient is counted once if the patient reported one or more events. Percentages will be calculated out of the number of patients in the Safety Set. The number of events at each level of SOC and PT will also be summarized.

The summary of TEAEs will be presented in descending order from the SOC with the highest total incidence (that is, summed across all treatment groups) to the SOC with the lowest total incidence. If the total incidence for any two or more SOCs is equal, the SOCs will be presented in alphabetical order. Within each SOC, the PTs will be presented in alphabetical order.

All adverse events will be listed including relationship and flag for treatment emergent ones.

9.1.3. Relationship of adverse events to study drug

The investigator will provide an assessment of the relationship of the event to the study drug. The possible relationships are “Unrelated”, “Unlikely”, “Possible” and “Probable”. "Unrelated”, “Unlikely” AEs will be categorized as “Not Related”. All other AEs: Possible”, “Probable” will be categorized as “Related”. TEAEs that are missing a relationship will be presented in the summary table as “Related” but will be presented in the data listing with a missing relationship. If a patient reports multiple occurrences of the same TEAE, only the most closely related occurrence will be presented.

9.1.4. Serious Adverse Events

The seriousness of an AE should be assessed by the investigator independently from the severity of the AE. A serious AE (SAE) is any untoward medical occurrence that at any dose results in death, is life threatening, requires inpatient hospitalization or prolongation of existing hospitalization, results in persistent or significant disability or incapacity, is a congenital anomaly or birth defect, or is an important medical event.

Other events to be treated as SAEs by the investigator are:

- **Exposure to drug during pregnancy:** In principle, pregnancy and the lactation period are exclusion criteria for clinical studies involving investigational drugs, which are not directly related to the respective conditions. In the event of a pregnancy occurring during the course of this particular study, the patient should be

withdrawn from study, but closely followed-up during the entire course of the pregnancy and postpartum period.

For missing dates, the rules stated in Section **Erreur ! Source du renvoi introuvable.** will be followed. If the imputed start date is prior to the AE start date, then the SAE start date should be imputed as the AE start date.

All serious adverse events will be listed including relationship and flag for treatment emergent ones.

9.1.4.1. Serious Adverse Events, related to study drug

The Serious TEAE data will be categorized and presented by SOC, PT, and relationship (i.e. "Related" and "Not Related") in a manner similar to that described in Section 9.1.2.

9.1.5. Adverse events leading to treatment discontinuation

A summary of TEAEs with a study drug action taken of "Product Discontinued" will be presented in a table in a similar manner to that described in Section 9.1.2.

9.1.6. Death

A summary of TEAEs where the answer to "Outcome" is "Fatal" will be presented in a table in a similar manner to that described in Section 9.1.2.

All patients who have an AE with an outcome of "Fatal" will be presented in a listing.

9.1.7. Overview summary

An overview summary of the number and percentage of patients with any TEAE, serious TEAE, study drug-related TEAE, study drug-related serious TEAE, TEAE leading to treatment discontinuation, TEAE leading to study discontinuation, and AE leading to death will be provided by treatment group.

9.2. Clinical laboratory evaluations

Laboratory assessments such as plasma biochemistry, hematology and coagulation will be obtained from local laboratories on the visit days specified in Section 11.1.

Summary tables presenting observed values and changes from baseline at each scheduled visit will be presented for numerical clinical laboratory tests by treatment group and overall. Summary box plots of observed values will be presented for numeric clinical laboratory results, by treatment group, including mean value over time. This will be for patients in the Safety Set.

If multiple values are collected within a post- baseline visit, the first value will be used in summary tables and figures. All values will be listed.

9.2.1. Hematology

The following hematology parameters will be summarized: hemoglobin, hematocrit, leucocytes, basophils, eosinophils, neutrophils, lymphocytes, monocytes and platelets count.

9.2.2. Plasma biochemistry

The following plasma biochemistry parameters will be summarized: aspartate aminotransferase, alanine aminotransferase, alkaline phosphatase, total bilirubin, creatinine, urea, glucose, sodium, potassium, chloride, calcium, inorganic phosphate, total protein, albumin, lactate, C Reactive Protein (CRP) and Ferritin.

9.2.3. Coagulation

The following coagulation parameters will be summarized: International Normalized Ratio (INR) and D Dimer.

9.2.4. Pregnancy test

Serum or urine pregnancy test will be performed in all female patients of childbearing potential at the Screening visit and will be listed for patients in the Safety Set.

9.3. Secondary Infection and Infection Type

Secondary infection and infection type assessments include site of infection, type of infection (Bacterial/Viral/Fungal/Combined), bacterial results (Positive/Negative) and antibiotics taken (Yes/No). Number and percentages will be calculated for patients in the Safety Set within the subgroup category. The number and percentage of patients with at least one secondary infection during the study, the number of infections per patient for patients with at least one infection, the site of infection and the Type of infection will be summarized by treatment group and overall and will be described by baseline sTREM-1 level (above or equal to the median vs below).

All data will be listed.

9.4. Co-administered Immunomodulatory Medications, Deaths, SAEs and Secondary Infections

At each part 2 analysis, the interaction between co-administered immunomodulatory medications and study drug will be reviewed by the DMC with data regarding the incidence of secondary infection, adverse events and death categorized by study drug and co-administered medication.

For the subgroup of patients who died, experienced SAE or secondary infections, a listing of presence/absence, start/end date and name of co-administered immunomodulatory medications will be produced by treatment group including presence/absence, start/end date of the event.

10. References

ICH E9 Statistical Principles for Clinical Trials.
https://www.ema.europa.eu/en/documents/scientific-guideline/ich-e-9-statistical-principles-clinical-trials-step-5_en.pdf

Cui, Lu., Hung, H.M. James. and Wang, Sue-Jane. (1999). Modification of sample size in groups sequential clinical trials. Biometrics. 55, 853-857

11. Appendices

11.1. Schedule of Study Procedures

	SCR						EoT			EoS[11]	FU[12] Blinded
**Assessment / Day[1]**	**≤48H**	**1**	**2**	**3**	**4**	**5**	**6**	**8 +2d**	**14 +4d**	**28[11] +7d**	**60 +7d**
Informed consent[2]	X[2]										
Inclusion/exclusion criteria	X										
Medical history and demographics	X										
Pregnancy test[3]	X										
Height and weight[4]	X										
Physical Examination	X										
Randomization	X										
Study Drug Infusion[5]		X	X	X	X	X	X				
Prior[6] and concomitant therapies	X	X	X	X	X	X	X	X	X	X	X[13]
Oxygenation and ventilation	X	X[10]	X	X	X	X	X	X	X	X	
Clinical Status[7]		X[10]	X	X	X	X	X	X	X	X	X
SOFA score		X[10]								X	
Thromboembolic events[7]		X[10]	X	X	X	X	X	X	X	X	
Secondary infections[7]		X[10]	X	X	X	X	X	X	X	X	
Adverse events[8]		X	X	X	X	X	X	X	X	X	X[14]
Hemodynamic support (vasopressor)	X	X[10]	X	X	X	X	X	X	X	X	
Renal replacement Therapy	X	X[10]	X	X	X	X	X	X	X	X	
Mortality		X	X	X	X	X	X	X	X	X	X
Clinical Laboratory (see table 2)	X	X[10]	X	X	X	X	X	X	X	X	
Procalcitonin (PCT) (see table 2)		X[10]			X		X	X	X	X	
sTREM-1 (see table 2)		X[10]			X		X	X	X	X	
IL-6 (see table 2)		X[10]			X		X	X	X	X	
Blood sample for retention (see table 2)		X[10]			X		X	X	X	X	
Discharge letter collection[9]										X	X
Quality of Life/Functional assessment											X

SCR : screening ; EoT: end of treatment; EoS : end of study	

1: Days refer to calendar days, day 1 is defined as the calendar day of first study drug administration
2: In case of emergency consent, confirmation of consent as soon as patient is capable
3: Women of childbearing potential
4: Estimate in case measurement is not feasible
5: Patients will be treated for 5 days (120 hours) or until ICU discharge, whichever is sooner
6: All medication taken within the last 24 hours before start of treatment should be documented. This does include any other study medication from other trials.
7: Assessments will be performed before IMP initiation (Day 1) thendaily until day 14. Further assessment will be made on day 28 and day 60 even in case patient is discharged from ICU.
8: AEs will be collected until day 28
9: Discharge letters from ICU discharge (where permissible and possible) at day 28 and day 60
10: Before first treatment administration
11: Day 28 and Day 60 visit may be completed by site via telephone call if visit to study center not possible.
12: By telephone or in person (Patients in part 2 or 3 only)
13: Review only of medications reported as ongoing at day 28
14: Drug related SAEs only after day 28

INOTREM

MOT-C-204 ESSENTIAL

Exploratory study of the safety, tolerability and efficacy of nangibotide in patients with COVID-19 receiving ventilatory support and features of systemic inflammation

A randomized, double-blind, placebo-controlled study with adaptive features

02DEC2022

Statistical Analysis Plan Addendum

Version 0.1

Prepared by:

PPD
Granta Park, Great Abington,
Cambridge, CB21 6GQ

TABLE OF CONTENTS

LIST OF ABBREVIATIONS III

1. INTRODUCTION 1

2. GENERAL STATISTICAL CONSIDERATIONS 1

2.1. TABULATIONS AND PRESENTATIONS 1
2.2. ANALYSIS SET 1

3. ADDITIONAL SUMMARIES 1

3.1. DISPOSITION 1
3.2. PROTOCOL DEVIATIONS 1
3.3. SURVIVAL AT DAY 28 AND DAY 60 2
3.4. CLINICAL STATUS 2
3.5. ARTERIAL BLOOD GAS AND PAO2:FIO2 RATIO 2
3.6. ADDITIONAL LISTINGS 2

4. REFERENCES 3

List of Abbreviations

CI	Confidence Interval
COVID-19	Coronavirus disease 2019
CRF	Case Report Form
EQ-5D	EuroQol (5 dimensions)
ICH	International Council for Harmonisation of Technical Requirements for Pharmaceuticals for Human Use
ITT	Intent-to-Treat
MITT	Modified Intent-to-Treat
PP	Per Protocol
SAP	Statistical Analysis Plan
sTREM-1	Soluble TREM-1
TREM-1	Triggering Receptor Expressed on Myeloid Cells

1. Introduction

The purpose of this post-unblinding and post-database lock Statistical Analysis Plan (SAP) Addendum is to describe additional summaries and additional statistical methods to those described in the main SAP of the MOT-C-204 study, Version 1.0, dated 02AUG2022 and approved before unblinding.

This statistical analysis plan is based on International Conference on Harmonisation (ICH) E3 and E9 Guidelines and on the study protocol MOT-C-204 Version 8.0 dated 01JUN2022.

2. General Statistical Considerations

2.1. Tabulations and Presentations

All summaries and results will be described and presented as indicated in the main SAP.

2.2. Analysis Set

There is no change in analysis sets definitions with regard to approved SAP before unblinding.

3. Additional Summaries

3.1. Disposition

Number and percentage of Randomized patients and in ITT Set, mITT Set, Safety Set and Per protocol Set will be summarized by treatment group and overall for the Randomized patients.

Patients inclusion in the different analysis sets will be listed for all enrolled subjects.

Randomization information, unblinding and kit numbers will be listed for the ITT Set.

3.2. Protocol Deviations

All protocol deviations will be listed. Major deviations are those which are considered to potentially impact upon the interpretation of the key efficacy endpoints in the study, or that may potentially impact on the interpretation of safety. Patients with major protocol deviations will be excluded from per-protocol analysis set (PP). The final list of protocol deviations with categorization into major or minor was finalized and documented prior to unblinding the study data.

3.3. Survival at Day 28 and Day 60

As planned in the protocol version 8.0, in sensitivity analyses of mortality at Day 28 and Day 60, a log-rank test stratified by baseline clinical status will be used to compare the treatment arms. In addition, a Proportional Hazard Cox model adjusting for treatment, baseline clinical status will be fitted to estimate the treatment effect expressed in terms of a hazard ratio with the 95% CI and p-value for Survival at Day 28 and Day 60. Survival curves will be produced. These analyses will be performed overall, for patients with baseline sTREM-1 below the median and for patients with baseline sTREM-1 equal or above the median separately.

3.4. Clinical Status

Clinical status (7-point scale and categorized as Improved/Unchanged/Worsened from baseline) will be displayed at each visit from Day 1 to Day 14 overall and by baseline sTREM-1 level.

3.5. Arterial Blood Gas and PaO2:FiO2 ratio

Change from baseline in PaO2/FiO2 Ratio from Day 2 to Day 6 and at Day 8 will be analyzed using the following categories:

- Worsening / Died
- >0%; <=10%
- >10%;<=20%
- >20%

These descriptive analyses will be performed overall, for patients with baseline sTREM-1 below the median and for patients with baseline sTREM-1 equal or above the median separately.

All arterial blood gas data will be listed for the mITT set.

3.6. Additional Listings

Health-related Quality of Life (EuroQol / EQ-5D) questionnaire data collected via telephone interview at Day 60 will be listed for the mITT Set.
Thromboembolic data collected in the CRF will be listed for the Safety Set.

4. References

EuroQol, G. (1990). EuroQol--a new facility for the measurement of health-related quality of life. Health Policy 16, 199-208.

ICH E9 Statistical Principles for Clinical Trials. https://www.ema.europa.eu/en/documents/scientific-guideline/ich-e-9-statistical-principles-clinical-trials-step-5_en.pdf
