# Supplementary material for: Evaluation of the efficacy and safety of TREM-1 inhibition with nangibotide in patients with COVID-19 receiving respiratory support: the ESSENTIAL randomised, double-blind trial
Source: eClinicalMedicine. 2023 May 31;60:102013. doi: 10.1016/j.eclinm.2023.102013 (PMC10231876; doi:10.1016/j.eclinm.2023.102013)
Supplement: ESSENTIAL ESM1 [file mmc5.docx]

Delphine Chartier (CHRU Tours-Hôpital Bretonneau Service de Réanimation Médicale et Polyvalente, 2 Boulevard Tonnellé, Tours 37000, France), Véronique Simeon-Vieules (CHRU Tours-Hôpital Bretonneau Service de Réanimation Médicale et Polyvalente, 2 Boulevard Tonnellé, Tours 37000, France), Sophie Jacquier (CHRU Tours-Hôpital Bretonneau Service de Réanimation Médicale et Polyvalente, 2 Boulevard Tonnellé, Tours 37000, France), Laetitia Bodet Contentin (CHRU Tours-Hôpital Bretonneau Service de Réanimation Médicale et Polyvalente, 2 Boulevard Tonnellé, Tours 37000, France), Denis Garot (CHRU Tours-Hôpital Bretonneau Service de Réanimation Médicale et Polyvalente, 2 Boulevard Tonnellé, Tours 37000, France), Pierre-Francois Dequin (CHRU Tours-Hôpital Bretonneau Service de Réanimation Médicale et Polyvalente, 2 Boulevard Tonnellé, Tours 37000, France), Stephan Ehrmann (CHRU Tours-Hôpital Bretonneau Service de Réanimation Médicale et Polyvalente, 2 Boulevard Tonnellé, Tours 37000, France), Antoine Guillon (CHRU Tours-Hôpital Bretonneau Service de Réanimation Médicale et Polyvalente, 2 Boulevard Tonnellé, Tours 37000, France), Annick Legras (CHRU Tours-Hôpital Bretonneau Service de Réanimation Médicale et Polyvalente, 2 Boulevard Tonnellé, Tours 37000, France), Charlotte Salmon (CHRU Tours-Hôpital Bretonneau Service de Réanimation Médicale et Polyvalente, 2 Boulevard Tonnellé, Tours 37000, France), Maeva Gourraud (CHRU Tours-Hôpital Bretonneau Service de Réanimation Médicale et Polyvalente, 2 Boulevard Tonnellé, Tours 37000, France), Lucile Rouault (CHRU Tours-Hôpital Bretonneau Service de Réanimation Médicale et Polyvalente, 2 Boulevard Tonnellé, Tours 37000, France), Lysiane Brick (CHRU Tours-Hôpital Bretonneau Service de Réanimation Médicale et Polyvalente, 2 Boulevard Tonnellé, Tours 37000, France), Emmanuel Gaillard (CHRU Tours-Hôpital Bretonneau Service de Réanimation Médicale et Polyvalente, 2 Boulevard Tonnellé, Tours 37000, France), Gaelle Fajolle (CHRU Tours-Hôpital Bretonneau Service de Réanimation Médicale et Polyvalente, 2 Boulevard Tonnellé, Tours 37000, France), Hélène Bansard (CHRU Tours-Hôpital Bretonneau Service de Réanimation Médicale et Polyvalente, 2 Boulevard Tonnellé, Tours 37000, France), Maeva Dieu (CHRU Tours-Hôpital Bretonneau Service de Réanimation Médicale et Polyvalente, 2 Boulevard Tonnellé, Tours 37000, France), Elodie Boulay (CHRU Tours-Hôpital Bretonneau Service de Réanimation Médicale et Polyvalente, 2 Boulevard Tonnellé, Tours 37000, France), Emmanuelle Mercier (CHRU Tours-Hôpital Bretonneau Service de Réanimation Médicale et Polyvalente, 2 Boulevard Tonnellé, Tours 37000, France), Silvine Taillard (CHRU Tours-Hôpital Bretonneau Service de Réanimation Médicale et Polyvalente, 2 Boulevard Tonnellé, Tours 37000, France), Charlotte Larrat (CHRU Tours-Hôpital Bretonneau Service de Réanimation Médicale et Polyvalente, 2 Boulevard Tonnellé, Tours 37000, France), Christophe Guitton (Centre Hospitalier Le Mans Service de Réanimation, 194 Avenue Rubillard, Le Mans 72037, France), Alain Robert (Centre Hospitalier Le Mans Service de Réanimation, 194 Avenue Rubillard, Le Mans 72037, France), Marie-Helene Leroyer (Centre Hospitalier Le Mans Service de Réanimation, 194 Avenue Rubillard, Le Mans 72037, France), Nicoles Chudeau (Centre Hospitalier Le Mans Service de Réanimation, 194 Avenue Rubillard, Le Mans 72037, France), Patrice Tirot (Centre Hospitalier Le Mans Service de Réanimation, 194 Avenue Rubillard, Le Mans 72037, France), Jean-Chrisophe Callahan (Centre Hospitalier Le Mans Service de Réanimation, 194 Avenue Rubillard, Le Mans 72037, France), Charlene Lemoal (Centre Hospitalier Le Mans Service de Réanimation, 194 Avenue Rubillard, Le Mans 72037, France), Juliette Meunier (Centre Hospitalier Le Mans Service de Réanimation, 194 Avenue Rubillard, Le Mans 72037, France), Eliott Le Basnier (Centre Hospitalier Le Mans Service de Réanimation, 194 Avenue Rubillard, Le Mans 72037, France), Cedric Darreau (Centre Hospitalier Le Mans Service de Réanimation, 194 Avenue Rubillard, Le Mans 72037, France), Marjorie Saint-Martin (Centre Hospitalier Le Mans Service de Réanimation, 194 Avenue Rubillard, Le Mans 72037, France), Remy Marnai (Centre Hospitalier Le Mans Service de Réanimation, 194 Avenue Rubillard, Le Mans 72037, France), Mickael Landais (Centre Hospitalier Le Mans Service de Réanimation, 194 Avenue Rubillard, Le Mans 72037, France), Sabine Janowski (Centre Hospitalier de Béthune Unité de recherche clinique, 27 Rue Delbecque, Béthune 62408, France), Anne Dewatine (Centre Hospitalier de Béthune Unité de recherche clinique, 27 Rue Delbecque, Béthune 62408, France), Christophe Vinsonneau (Centre Hospitalier de Béthune Service de Réanimation et Surveillance continue, 27 Rue Delbecque, Béthune 62408, France), Caroline Sejourne (Centre Hospitalier de Béthune Service de Réanimation et Surveillance continue, 27 Rue Delbecque, Béthune 62408, France), Martin Gerard (Centre Hospitalier de Béthune Service de Réanimation et Surveillance continue, 27 Rue Delbecque, Béthune 62408, France), Pascal Andreu (CHRU Dijon Complexe Du Bocage Service De Réanimation, 14 Rue Gaffarel, Dijon 21079, France), Francois Aptel (CHRU Dijon Complexe Du Bocage Service De Réanimation, 14 Rue Gaffarel, Dijon 21079, France), Marie Labruyere (CHRU Dijon Complexe Du Bocage Service De Réanimation, 14 Rue Gaffarel, Dijon 21000, France), Jean-Baptiste Roudaut (CHRU Dijon Complexe Du Bocage Service De Réanimation, 14 Rue Gaffarel, Dijon 21079, France), Mathilde Audry (CHRU Dijon Complexe Du Bocage Service De Réanimation, 14 Rue Gaffarel, Dijon 21079, France), Solenne Villot (CHRU Dijon Complexe Du Bocage Service De Réanimation, 14 Rue Gaffarel, Dijon 21079, France), Ludivine Garrier (CHRU Dijon Complexe Du Bocage Service De Réanimation, 14 Rue Gaffarel, Dijon 21000, France), Jean-Pierre Quenot (CHRU Dijon Complexe Du Bocage Service De Réanimation, 14 rue Gaffarel, Dijon 21079, France), Hayat Allam (CHU de Strasbourg Service de Réanimation Médicale, 1 Place de l'hôpital, Strasbourg 67091, France), Ferhat Meziani (CHU de Strasbourg Service de Réanimation Médicale, 1 Place de l'hôpital, Strasbourg 67091, France), Hamid Merdji (CHU de Strasbourg Service de Réanimation Médicale, 1 Place de l'hôpital, Strasbourg 67091, France), Alexandra Monnier (CHU de Strasbourg Service de Réanimation Médicale, 1 Place de l'hôpital, Strasbourg 67091, France), Antoine Studer (CHU de Strasbourg Service de Réanimation Médicale, 1 Place de l'hôpital, Strasbourg 67091, France), Christine Kummerlen (CHU de Strasbourg Service de Réanimation Médicale, 1 Place de l'hôpital, Strasbourg 67091, France), Leonie Thiebaut (CHU de Strasbourg Service de Réanimation Médicale, 1 Place de l'hôpital, Strasbourg 67091, France), Ouafa Hakkari (CHU de Strasbourg Service de Réanimation Médicale, 1 Place de l'hôpital, Strasbourg 67091, France), Léa Dudenhoeffer (CHU de Strasbourg Service de Réanimation Médicale, 1 Place de l'hôpital, Strasbourg 67091, France), Hassene Rahmani (CHU de Strasbourg Service de Réanimation Médicale, 1 Place de l'hôpital, Strasbourg 67091, France), Louise-Marie Jandeaux (CHU de Strasbourg Service de Réanimation Médicale, 1 Place de l'hôpital, Strasbourg 67091, France), Julien Demiselle (CHU de Strasbourg Service de Réanimation Médicale, 1 Place de l'hôpital, Strasbourg 67091, France), Julie Helms (CHU de Strasbourg Service de Réanimation Médicale, 1 Place de l'hôpital, Strasbourg 67091, France), Séverine Bitsch (Hôpital Nord Franche-Comté Unité de réanimation, 100 route de Moval, Trevenans 90015, France), Alexia Rohmer (Hôpital Nord Franche-Comté Unité de réanimation, 100 route de Moval, Trevenans 90015, France), Delphine Korner (Hôpital Nord Franche-Comté Unité de réanimation, 100 route de Moval, Trevenans 90015, France), Hakim Slimani (Hôpital Nord Franche-Comté Unité de réanimation, 100 route de Moval, Trevenans 90015, France), Stefan Georgiev (Hôpital Nord Franche-Comté Unité de réanimation, 100 route de Moval, Trevenans 90015, France), Chaouki Mezher (Hôpital Nord Franche-Comté Unité de réanimation, 100 route de Moval, Trevenans 90015, France), Elodie Bouvier (Hôpital Nord Franche-Comté Unité de réanimation, 100 route de Moval, Trevenans 90015, France), Sylvain Malfroy (Hôpital Nord Franche-Comté Unité de réanimation, 100 route de Moval, Trevenans 90015, France), Amish-Bhaskar Seeruttun (Hôpital Nord Franche-Comté Unité de réanimation, 100 route de Moval, Trevenans 90015, France), Charlotte Bourgoin (Hôpital Nord Franche-Comté Unité de réanimation, 100 route de Moval, Trevenans 90015, France), Nahila Himer (Hôpital Nord Franche-Comté Unité de réanimation, 100 route de Moval, Trevenans 90015, France), Julio Badie (Hôpital Nord Franche-Comté Unité de réanimation, 100 route de Moval, Trevenans 90015, France), Angélique Reyen (Hôpital Nord Franche-Comté Unité de réanimation, 100 route de Moval, Trevenans 90015, France), Nadezhda-Yanakieva Georgieva (Hôpital Nord Franche-Comté Unité de réanimation, 100 route de Moval, Trevenans 90015, France), Laurent Faivre (Hôpital Nord Franche-Comté Unité de réanimation, 100 route de Moval, Trevenans 90015, France), Natacha Maquigneau (Centre Hospitalier Départemental de Vendée Service de réanimation polyvalente, Boulevard Stéphane Moreau, La Roche-Sur-Yon 85925, France), Jean-Claude Lacherade (Centre Hospitalier Départemental de Vendée Service de réanimation polyvalente, Boulevard Stéphane Moreau, La Roche-Sur-Yon 85925, France), Marie-Ange Azais (Centre Hospitalier Départemental de Vendée Service de réanimation polyvalente, Boulevard Stéphane Moreau, La Roche-Sur-Yon 85925, France), Konstantinos Bachoumas (Centre Hospitalier Départemental de Vendée Service de réanimation polyvalente, Boulevard Stéphane Moreau, La Roche-Sur-Yon 85925, France), Gwenhael Colin (Centre Hospitalier Départemental de Vendée Service de réanimation polyvalente, Boulevard Stéphane Moreau, La Roche-Sur-Yon 85925, France), Maud Fiancette (Centre Hospitalier Départemental de Vendée Service de réanimation polyvalente, Boulevard Stéphane Moreau, La Roche-Sur-Yon 85925, France), Matthieu Henry-Lagarrigue (Centre Hospitalier Départemental de Vendée Service de réanimation polyvalente, Boulevard Stéphane Moreau, La Roche-Sur-Yon 85925, France), Hugo Hille (Centre Hospitalier Départemental de Vendée Service de réanimation polyvalente, Boulevard Stéphane Moreau, La Roche-Sur-Yon 85925, France), Christine Lebert (Centre Hospitalier Départemental de Vendée Service de réanimation polyvalente, Boulevard Stéphane Moreau, La Roche-Sur-Yon 85925, France), Laurent Martin-Lefevre (Centre Hospitalier Départemental de Vendée Service de réanimation polyvalente, Boulevard Stéphane Moreau, La Roche-Sur-Yon 85925, France), Pierre Millour (Centre Hospitalier Départemental de Vendée Service de réanimation polyvalente, Boulevard Stéphane Moreau, La Roche-Sur-Yon 85925, France), Caroline Pouplet (Centre Hospitalier Départemental de Vendée Service de réanimation polyvalente, Boulevard Stéphane Moreau, La Roche-Sur-Yon 85925, France), Aurelia Toussaint (Centre Hospitalier Départemental de Vendée Service de réanimation polyvalente, Boulevard Stéphane Moreau, La Roche-Sur-Yon 85925, France), Isabelle Vinatier (Centre Hospitalier Départemental de Vendée Service de réanimation polyvalente, Boulevard Stéphane Moreau, La Roche-Sur-Yon 85925, France), Aihem Yehia (Centre Hospitalier Départemental de Vendée Service de réanimation polyvalente, Boulevard Stéphane Moreau, La Roche-Sur-Yon 85925, France), Vanessa Zinzoni (Centre Hospitalier Départemental de Vendée Service de réanimation polyvalente, Boulevard Stéphane Moreau, La Roche-Sur-Yon 85925, France), Cécile Rousseau (Centre Hospitalier Départemental de Vendée Service de réanimation polyvalente, Boulevard Stéphane Moreau, La Roche-Sur-Yon 85925, France), Hélène Pelerin (Centre Hospitalier Départemental de Vendée Service de réanimation polyvalente, Boulevard Stéphane Moreau, La Roche-Sur-Yon 85925, France), Aurélie Cravoisy (Hôpital Central Service de Réanimation médicale, 29, Avenue du Maréchal de Lattre de Tassigny, Nancy 54035, France), Marie Conrad (Hôpital Central Service de Réanimation médicale, 29, Avenue du Maréchal de Lattre de Tassigny, Nancy 54035, France), Sébastien Gibot (Hôpital Central Service de Réanimation médicale, 29, Avenue du Maréchal de Lattre de Tassigny, Nancy 54035, France), Lionel Nace (Hôpital Central Service de Réanimation médicale, 29, Avenue du Maréchal de Lattre de Tassigny, Nancy 54035, France), Pierre-Edouard Bollaert (Hôpital Central Service de Réanimation médicale, 29, Avenue du Maréchal de Lattre de Tassigny, Nancy 54035, France), Guilhem Courte (Hôpital Central Service de Réanimation médicale, 29, Avenue du Maréchal de Lattre de Tassigny, Nancy 54035, France), Anne Baina (Hôpital Central Service de Réanimation médicale, 29, Avenue du Maréchal de Lattre de Tassigny, Nancy 54035, France), Nathalie Dumont (Hôpital Central Service de Réanimation médicale, 29, Avenue du Maréchal de Lattre de Tassigny, Nancy 54035, France), Anne Chatelain (Hôpital Central Service de Réanimation médicale, 29, Avenue du Maréchal de Lattre de Tassigny, Nancy 54035, France), Claire Fontanier (Hôpital Universitaire Dupuytren Service de Réanimation Polyvalente, 2 Avenue Martin Luther King, Limoges 87042, France), Paul Bourzeix (Hôpital Universitaire Dupuytren Service de Réanimation Polyvalente, 2 Avenue Martin Luther King, Limoges 87042, France), Ludmila Baudrillart (Hôpital Universitaire Dupuytren Service de Réanimation Polyvalente, 2 Avenue Martin Luther King, Limoges 87042, France), Bruno Francois (Hôpital Universitaire Dupuytren Service de Réanimation Polyvalente, 2 Avenue Martin Luther King, Limoges 87042, France), Isabelle Herafa (Hôpital Universitaire Dupuytren Service de Réanimation Polyvalente, 2 Avenue Martin Luther King, Limoges 87042, France), Jean-François Mary (Hôpital Universitaire Dupuytren Service de Réanimation Polyvalente, 2 Avenue Martin Luther King, Limoges 87042, France), Alexandra Gay (Hôpital Universitaire Dupuytren Service de Réanimation Polyvalente, 2 Avenue Martin Luther King, Limoges 87042, France), Guillaume Gilbert (Hôpital Universitaire Dupuytren Service de Réanimation Polyvalente, 2 Avenue Martin Luther King, Limoges 87042, France), Bruno Evrard (Hôpital Universitaire Dupuytren Service de Réanimation Polyvalente, 2 Avenue Martin Luther King, Limoges 87042, France), Marine Goudelin (Hôpital Universitaire Dupuytren Service de Réanimation Polyvalente, 2 Avenue Martin Luther King, Limoges 87042, France), Anne-Laure Fedou (Hôpital Universitaire Dupuytren Service de Réanimation Polyvalente, 2 Avenue Martin Luther King, Limoges 87042, France), Arnaud Desachy (Hôpital Universitaire Dupuytren Service de Réanimation Polyvalente, 2 Avenue Martin Luther King, Limoges 87042, France), Thomas Daix (Hôpital Universitaire Dupuytren Service de Réanimation Polyvalente, 2 Avenue Martin Luther King, Limoges 87042, France), Philippe Vignon (Hôpital Universitaire Dupuytren Service de Réanimation Polyvalente, 2 Avenue Martin Luther King, Limoges 87042, France), Julien Vaidie (Hôpital Universitaire Dupuytren Service de Réanimation Polyvalente, 2 Avenue Martin Luther King, Limoges 87042, France), Amandine Sanson (Hôpital Universitaire Dupuytren Service de Réanimation Polyvalente, 2 Avenue Martin Luther King, Limoges 87042, France), Celine Prevost (Hôpital Universitaire Dupuytren Service de Réanimation Polyvalente, 2 Avenue Martin Luther King, Limoges 87042, France), Perrine Engel (Hôpital Universitaire Dupuytren Service de Réanimation Polyvalente, 2 Avenue Martin Luther King, Limoges 87042, France), Florence Sanchez (Hôpital Universitaire Dupuytren Service de Réanimation Polyvalente, 2 Avenue Martin Luther King, Limoges 87042, France), Anne-Aurore Duchambon (Hôpital Universitaire Dupuytren Service de Réanimation Polyvalente, 2 Avenue Martin Luther King, Limoges 87042, France), Francois Beloncle (CHU Angers Service De Réanimation Médicale, 4 Rue Larrey, Angers 49100, France), Achille Kouatchet (CHU Angers Service De Réanimation Médicale, 4 Rue Larrey, Angers 49100, France), Alain Mercat (CHU Angers Service De Réanimation Médicale, 4 Rue Larrey, Angers 49100, France), Marc Pierrot (CHU Angers Service De Réanimation Médicale, 4 Rue Larrey, Angers 49100, France), Vincent Souday (CHU Angers Service De Réanimation Médicale, 4 Rue Larrey, Angers 49100, France), Laure Masson (CHU Angers Service De Réanimation Médicale, 4 Rue Larrey, Angers 49100, France), Satar Mortaza (CHU Angers Service De Réanimation Médicale, 4 Rue Larrey, Angers 49100, France), Paul Jaubert (CHU Angers Service De Réanimation Médicale, 4 Rue Larrey, Angers 49100, France), Alice Rouillard (CHU Angers Service De Réanimation Médicale, 4 Rue Larrey, Angers 49100, France), Hélène Julien (CHU Angers Service De Réanimation Médicale, 4 Rue Larrey, Angers 49100, France), Pierre Asfar (CHU Angers Service De Réanimation Médicale, 4 Rue Larrey, Angers 49100, France), Marie Clerc (CHU Angers Service De Réanimation Médicale, 4 Rue Larrey, Angers 49100, France), Elise Ballarini (CHU Angers Service De Réanimation Médicale, 4 Rue Larrey, Angers 49100, France), Florent Beaumale (CHU Angers Service De Réanimation Médicale, 4 Rue Larrey, Angers 49100, France), Pierre-Yves Olivier (CHU Angers Service De Réanimation Médicale, 4 Rue Larrey, Angers 49100, France), Jeremie Lemarie (Hôtel Dieu - Nantes Service de Réanimation Medicale Polyvalente, 30 Boulevard Jean Monnet, Nantes 44000, France), Virginie Delecrin (Hôtel Dieu - Nantes Service de Réanimation Medicale Polyvalente, 30 Boulevard Jean Monnet, Nantes 44000, France), Gauthier Blonz (Hôtel Dieu - Nantes Service de Réanimation Medicale Polyvalente, 30 Boulevard Jean Monnet, Nantes 44000, France), Luc Desmedt (Hôtel Dieu - Nantes Service de Réanimation Medicale Polyvalente, 30 Boulevard Jean Monnet, Nantes 44000, France), Paul Decamps (Hôtel Dieu - Nantes Service de Réanimation Medicale Polyvalente, 30 Boulevard Jean Monnet, Nantes 44000, France), Amelie Seguin (Hôtel Dieu - Nantes Service de Réanimation Medicale Polyvalente, 30 Boulevard Jean Monnet, Nantes 44000, France), Catherine Roussel (Hôtel Dieu - Nantes Service de Réanimation Medicale Polyvalente, 30 Boulevard Jean Monnet, Nantes 44000, France), Celine Rouzeau (Hôtel Dieu - Nantes Service de Réanimation Medicale Polyvalente, 30 Boulevard Jean Monnet, Nantes 44000, France), Melaine Le Brazic (Hôtel Dieu - Nantes Service de Réanimation Medicale Polyvalente, 30 Boulevard Jean Monnet, Nantes 44000, France), Carole Ouisse (Hôtel Dieu - Nantes Service de Réanimation Medicale Polyvalente, 30 Boulevard Jean Monnet, Nantes 44000, France), Olivier Zambon (Hôtel Dieu - Nantes Service de Réanimation Medicale Polyvalente, 30 Boulevard Jean Monnet, Nantes 44000, France), Maelle Martin (Hôtel Dieu - Nantes Service de Réanimation Medicale Polyvalente, 30 Boulevard Jean Monnet, Nantes 44000, France), Diane Maugars (Hôtel Dieu - Nantes Service de Réanimation Medicale Polyvalente, 30 Boulevard Jean Monnet, Nantes 44000, France), Charlotte Garret (Hôtel Dieu - Nantes Service de Réanimation Medicale Polyvalente, 30 Boulevard Jean Monnet, Nantes 44000, France), Pauline Lamouche-Wilquin (Hôtel Dieu - Nantes Service de Réanimation Medicale Polyvalente, 30 Boulevard Jean Monnet, Nantes 44000, France), Jean Reignier (Hôtel Dieu - Nantes Service de Réanimation Medicale Polyvalente, 30 Boulevard Jean Monnet, Nantes 44000, France), Emmanuel Canet (Hôtel Dieu - Nantes Service de Réanimation Medicale Polyvalente, 30 Boulevard Jean Monnet, Nantes 44000, France), Jean-Baptiste Lascarrou (Hôtel Dieu - Nantes Service de Réanimation Medicale Polyvalente, 30 Boulevard Jean Monnet, Nantes 44000, France), Paul Jaubert (Groupe Hospitalier Cochin Service de Réanimation Médicale, 27 Rue Du Faubourg Saint Jacques, Paris 75014, France), Jean-Paul Mira (Groupe Hospitalier Cochin Service de Réanimation Médicale, 27 Rue Du Faubourg Saint Jacques, Paris 75014, France), Juliette Pelle (Groupe Hospitalier Cochin Service de Réanimation Médicale, 27 Rue Du Faubourg Saint Jacques, Paris 75014, France), Nathalie Marin (Groupe Hospitalier Cochin Service de Réanimation Médicale, 27 Rue Du Faubourg Saint Jacques, Paris 75014, France), Minh Pierre Le (Groupe Hospitalier Cochin Service de Réanimation Médicale, 27 Rue Du Faubourg Saint Jacques, Paris 75014, France), Pierre Dupland (Groupe Hospitalier Cochin Service de Réanimation Médicale, 27 Rue Du Faubourg Saint Jacques, Paris 75014, France), Ariane Gavaud (Groupe Hospitalier Cochin Service de Réanimation Médicale, 27 Rue Du Faubourg Saint Jacques, Paris 75014, France), Sarah Benghanem (Groupe Hospitalier Cochin Service de Réanimation Médicale, 27 Rue Du Faubourg Saint Jacques, Paris 75014, France), Frederic Pene (Groupe Hospitalier Cochin Service de Réanimation Médicale, 27 Rue Du Faubourg Saint Jacques, Paris 75014, France), Swann Bredin (Groupe Hospitalier Cochin Service de Réanimation Médicale, 27 Rue Du Faubourg Saint Jacques, Paris 75014, France), Julien Charpentier (Groupe Hospitalier Cochin Service de Réanimation Médicale, 27 Rue Du Faubourg Saint Jacques, Paris 75014, France), Alain Cariou (Groupe Hospitalier Cochin Service de Réanimation Médicale, 27 Rue Du Faubourg Saint Jacques, Paris 75014, France), Suzanne Renard (Cliniques Universitaires Saint-Luc Soins Intensifs, Avenue Hippocrate 10, Bruxelles 1200, Belgium), Xavier Wittebole (Cliniques Universitaires Saint-Luc Soins Intensifs, Avenue Hippocrate 10, Bruxelles 1200, Belgium), Virginie Montiel (Cliniques Universitaires Saint-Luc Soins Intensifs, Avenue Hippocrate 10, Bruxelles 1200, Belgium), Pierre-Francois Laterre (Cliniques Universitaires Saint-Luc Soins Intensifs, Avenue Hippocrate 10, Bruxelles 1200, Belgium), Jean-Baptiste Mesland (Cliniques Universitaires Saint-Luc Soins Intensifs, Avenue Hippocrate 10, Bruxelles 1200, Belgium), Vinciane De Backer (Cliniques Universitaires Saint-Luc Soins Intensifs, Avenue Hippocrate 10, Bruxelles 1200, Belgium), Marie-France Dujardin (Cliniques Universitaires Saint-Luc Soins Intensifs, Avenue Hippocrate 10, Bruxelles 1200, Belgium), Caroline Berghe (Cliniques Universitaires Saint-Luc Soins Intensifs, Avenue Hippocrate 10, Bruxelles 1200, Belgium), Philippe Hantson (Cliniques Universitaires Saint-Luc Soins Intensifs, Avenue Hippocrate 10, Bruxelles 1200, Belgium), Ludovic Gerard (Cliniques Universitaires Saint-Luc Soins Intensifs, Avenue Hippocrate 10, Bruxelles 1200, Belgium), Christine Collienne (Cliniques Universitaires Saint-Luc Soins Intensifs, Avenue Hippocrate 10, Bruxelles 1200, Belgium), Kim Engelen (Ziekenhuis Oost-Limburg Intensieve Geneeskunde, Schiepse Bos 6, Genk 3600, Belgium), Dieter Mesotten (Ziekenhuis Oost-Limburg Intensieve Geneeskunde, Schiepse Bos 6, Genk 3600, Belgium), Lore Busse (Ziekenhuis Oost-Limburg Intensieve Geneeskunde, Schiepse Bos 6, Genk 3600, Belgium), Stéphane Deman (Ziekenhuis Oost-Limburg Intensieve Geneeskunde, Schiepse Bos 6, Genk 3600, Belgium), Shaun De Meirsman (Ziekenhuis Oost-Limburg Intensieve Geneeskunde, Schiepse Bos 6, Genk 3600, Belgium), Cédric Bouts (Ziekenhuis Oost-Limburg Intensieve Geneeskunde, Schiepse Bos 6, Genk 3600, Belgium), Steven Thiessen (Ziekenhuis Oost-Limburg Intensieve Geneeskunde, Schiepse Bos 6, Genk 3600, Belgium), Jonathan Conings (Ziekenhuis Oost-Limburg Intensieve Geneeskunde, Schiepse Bos 6, Genk 3600, Belgium), Tom Fivez (Ziekenhuis Oost-Limburg Intensieve Geneeskunde, Schiepse Bos 6, Genk 3600, Belgium), Margot Vander Laenen (Ziekenhuis Oost-Limburg Intensieve Geneeskunde, Schiepse Bos 6, Genk 3600, Belgium), Xavier Willaert (Ziekenhuis Oost-Limburg Intensieve Geneeskunde, Schiepse Bos 6, Genk 3600, Belgium), Sam Van Boxstael (Ziekenhuis Oost-Limburg Intensieve Geneeskunde, Schiepse Bos 6, Genk 3600, Belgium), Ingrid Meex (Ziekenhuis Oost-Limburg Intensieve Geneeskunde, Schiepse Bos 6, Genk 3600, Belgium), Elly Vandermeulen (Ziekenhuis Oost-Limburg Intensieve Geneeskunde, Schiepse Bos 6, Genk 3600, Belgium), Diana Clemente Batalha Pardal (Ziekenhuis Oost-Limburg Intensieve Geneeskunde, Schiepse Bos 6, Genk 3600, Belgium), Willem Boer (Ziekenhuis Oost-Limburg Intensieve Geneeskunde, Schiepse Bos 6, Genk 3600, Belgium)

Sven Zimmermann (Inotrem S.A , 54 rue de Ponthieu, 75008 Paris, France), Jean-Jacques Garaud (Inotrem S.A , 54 rue de Ponthieu, 75008 Paris, France), Martin Koch (Inotrem S.A , 54 rue de Ponthieu, 75008 Paris, France), Marc Derive (Inotrem S.A , 54 rue de Ponthieu, 75008 Paris, France), Margarita Salcedo-Magguilli (Inotrem S.A , 54 rue de Ponthieu, 75008 Paris, France), Simon Lambden (Inotrem S.A , 54 rue de Ponthieu, 75008 Paris, France), Sybille Dubois (Inotrem S.A , 54 rue de Ponthieu, 75008 Paris, France), Myriam Dumas (Inotrem S.A , 54 rue de Ponthieu, 75008 Paris, France), Valérie Cuvier (Inotrem S.A , 54 rue de Ponthieu, 75008 Paris, France), Philippe Bridonneau (Inotrem S.A , 54 rue de Ponthieu, 75008 Paris, France), Aurélie Olivier (Inotrem S.A , 54 rue de Ponthieu, 75008 Paris, France), Stephan Witte (Inotrem S.A , 54 rue de Ponthieu, 75008 Paris, France), Marie-Françoise Santoja (Inotrem S.A , 54 rue de Ponthieu, 75008 Paris, France), Lucie Jolly (Inotrem S.A , 54 rue de Ponthieu, 75008 Paris, France), Frederic Marmion (Inotrem S.A , 54 rue de Ponthieu, 75008 Paris, France), Delphine Joyeux (Inotrem S.A , 54 rue de Ponthieu, 75008 Paris, France), Monica Bidounga (Inotrem S.A , 54 rue de Ponthieu, 75008 Paris, France), Tarik Hicheur (Inotrem S.A , 54 rue de Ponthieu, 75008 Paris, France), Anne-Lise Laszlo-Pouvreau (Inotrem S.A , 54 rue de Ponthieu, 75008 Paris, France), Emma Lafond-Thenaille (Inotrem S.A , 54 rue de Ponthieu, 75008 Paris, France), Laurent Vincent (Inotrem S.A , 54 rue de Ponthieu, 75008 Paris, France), Nicolas Fourrier (Inotrem S.A , 54 rue de Ponthieu, 75008 Paris, France)

Jean-Marie Grouin (statistician)

Bruno François (Steering committee membership), Pierre-François Laterre (Steering committee membership), Mitchell Levy (Steering committee membership)

Michel Wolff (Data monitoring committee membership), Jean Chastre (Data monitoring committee membership), Steven Opal (Data monitoring committee membership), Stephen Senn (Data monitoring committee membership)
