# Supplementary material for: Evaluation of the efficacy and safety of TREM-1 inhibition with nangibotide in patients with COVID-19 receiving respiratory support: the ESSENTIAL randomised, double-blind trial
Source: eClinicalMedicine. 2023 May 31;60:102013. doi: 10.1016/j.eclinm.2023.102013 (PMC10231876; doi:10.1016/j.eclinm.2023.102013)
Supplement: ESSENTIAL-eClinicalMedicine-ESM2-R1-CLEAN [file mmc6.docx]

Title:

**Evaluation of the efficacy and safety of TREM-1 inhibition with nangibotide in patients with COVID-19 receiving respiratory support: The ESSENTIAL study**

Authors:

François, Bruno, MD^1^ , Lambden, Simon, MBBS^2,3^, Garaud, Jean-Jacques, MD^3^ , Derive, Marc, PhD^3^, Grouin, Jean Marie, PhD^4^, Asfar, Pierre, MD^5^, Darreau, Cédric, MD^6^, Mira, Jean-Paul, MD^7^, Quenot, Jean-Pierre, MD^8^, Lemarié, Jérémie, MD^9^, Mercier, Emmanuelle, MD^10^, Lacherade, Jean-Claude, MD^11^, Vinsonneau, Christophe, MD^12^, Fivez, Tom, MD^13^, Helms, Julie, MD^14^, Badie, Julio, MD^15^, Levy, Mitchell, MD^16^, Cuvier, Valérie^3^, Salcedo-Magguilli, Margarita, PhD^3,^ , Laszlo-Pouvreau, Anne-Lise, MSc^3^,Laterre, Pierre-François, MD^17^, Gibot, Sébastien, MD^18^ on behalf of the ESSENTIAL investigators

Affiliations:

1: Medical‑Surgical ICU department and Inserm CIC1435 & UMR1092, CHU Dupuytren, Limoges, France

2: Department of Medicine, University of Cambridge, Cambridge, UK

3: Inotrem SA, Paris, France

4: Université de Rouen, 76821 Mont Saint-Aignan, France

5: Department of intensive care, CHU d’Angers, France

6: Department of intensive care, CHU Le Mans, France

7 : Department of intensive care, Groupe hospitalier Cochin, Paris, France

8: Department of intensive care, CHRU Dijon Complexe du Bocage, Dijon, France

9 : Department of intensive care, Hôtel Dieu, Nantes, France

10: Department of intensive care, CHRU Tours Hôpital Bretonneau, Tours, France

11 : Department of intensive care, Centre hospitalier Départemental de Vendée, La Roche-Sur-Yon, France

12: Department of intensive care, Centre Hospitalier de Béthunes, France

13: Department of intensive care, Ziekenhuis Oost-Limburg, Genk, Belgium

14: Department of intensive care, Hôpitaux universitaires de Strasbourg, Nouvel Hôpital Civil, Université de Strasbourg (UNISTRA), Faculté de Médecine and Inserm UMR 1260, RNM, FMTS, Strasbourg, France

15 : Department of intensive care, Hôpital Nord Franche-Comté,Trevenans, France

16: Division of Pulmonary, Critical Care and Sleep Medicine, Department of Medicine, Warren Alpert School of Medicine at Brown University, Providence, RI, USA

17: Department of Critical Care Medicine, St Luc University Hospital, Université Catholique de Louvain, Brussels, Belgium

18: Intensive Care Unit, Centre Hospitalier Regional Universitaire (CHRU), 54000 Nancy, France.

***Corresponding author:**

Dr Bruno Francois

ICU Department – CHU Dupuytren

2 avenue Martin-Luther King

87072 Limoges cedex, France

bruno.francois@chu-limoges.fr

**Inclusion and Exclusion Criteria**

**Inclusion Criteria:**

To be eligible for the study, patients must meet the following criteria:

1. Provided informed consent (emergency consent according to local regulations where approved)
2. Age 18 to 75 years (inclusive)
3. Admitted to an intensive care unit
4. Treatment with High Flow Nasal Oxygen, non-invasive ventilation or invasive mechanical ventilation for acute respiratory failure caused by COVID-19 for less than 48 hours
5. A PaO2:FiO2 ratio of <200mmHg (<26.7kPa) with a FiO2 ≥0.6
6. Confirmed laboratory diagnosis of COVID-19 within 7 days of meeting screening criteria

**Exclusion Criteria**

The presence of any of the following criteria will exclude a patient from study enrolment:

1. Known pregnancy (positive urine or serum pregnancy test)
2. Ongoing treatment with an immunomodulatory agent not included in the standard of care for COVID-19 (including participation in clinical trials of such agents).
3. Body mass index (BMI) ≥ 40 kg/m2or weight ≥ 130 kg
4. Anticipated transfer to another hospital, which is not a study site within 72 hours
5. Expected to die within 6 months of treatment due to underlying chronic disease
6. Limitations of care in place during current hospital admission

**Supplementary Methods**

Methodology of the primary outcome analysis

The clinical status is an ordinal variable and the stratified Cochran-Mantel-Haenszel (CMH) analysis of the ordinal variable was planned as it is widely employed in regulatory preferred analytical strategies, such as the primary analysis, for this kind of data. As it is an ordinal analysis, it is necessary to propose scores associated with the variable values. Of note, the CMH analysis using modified ridit scores is the contingency counterpart of the Wilcoxon test. The p-value of the Wald test obtained in an ordinal logistic regression model assuming odds proportionality was found to be not far away from the p-value of the CMH test. Analyses considering a binary outcome are indeed of interest and were also planned in the SAP and performed.

Day 60 outcome analysis

Day 60 outcome was evaluated in patients included in part one of the study after completion of the trial following an amendment to the study protocol. Patients included in part one of the study were contacted and permission sought to include their vital status at day 60 in the analysis of the study. In addition, clinical data was collected from the national database of COVID-19 deaths and from hospital records at the study sites. Of the 60 patients included in part one of the study, data was available for 58 of the participants.

**Supplementary results**

Supplementary table 1: Definitions of each category of patients within the 7-point ordinal scale. All patients scored either 5 or 6 at baseline***.***

| Value | Clinical Status Definition |
| --- | --- |
| **1** | Not hospitalized, no limitations of activities |
| **2** | Not hospitalized, limitations of activities |
| **3** | Hospitalized, not requiring supplemental oxygen |
| **4** | Hospitalized, requiring supplemental oxygen |
| **5** | Hospitalized, on non-invasive ventilation or high flow oxygen devices |
| **6** | Hospitalized, on invasive mechanical ventilation or ECMO |
| **7** | Death |

Supplementary table 2: Distribution of clinical status at day 28 in patients that had a baseline clinical status score of 5 in the overall and high sTREM-1 populations

|  | | **Overall Population** | | **High sTREM-1** | |
| --- | --- | --- | --- | --- | --- |
|  |  | **Placebo** | **Nangibotide** | **Placebo** | **Nangibotide** |
|  |  | n(%) | n(%) | n(%) | n(%) |
| Value | Clinical Status Definition |  | |  | |
| **1** | Not hospitalized, no limitations of activities | 16 (23.2) | 17 (21.3) | 5 (13.9) | 1 (3.1) |
| **2** | Not hospitalized, limitations of activities | 13 (18.8) | 25 (31.3) | 5 (13.9) | 8 (25.0) |
| **3** | Hospitalized, not requiring supplemental oxygen | 4 (5.8) | 6 (7.5) | 2 (5.6) | 3 (9.4) |
| **4** | Hospitalized, requiring supplemental oxygen | 9 (13.0) | 10 (12.5) | 3 (8.3) | 7 (21.9) |
| **5** | Hospitalized, on non-invasive ventilation or high flow oxygen devices | 1 (1.4) | 0 (0.0) | 1 (2.8) | 0 (0.0) |
| **6** | Hospitalized, on invasive mechanical ventilation or ECMO | 11 (15.9) | 13 (16.3) | 7 (19.4) | 7 (21.9) |
| **7** | Death | 15 (21.7) | 9 (11.3) | 13 (36.1) | 6 (18.8) |

Supplementary table 3: Distribution of clinical status at day 28 in patients that had a baseline clinical status score of 6 in the overall and high sTREM-1 populations

|  | | **Overall Population** | | **High sTREM-1** | |
| --- | --- | --- | --- | --- | --- |
|  |  | **Placebo** | **Nangibotide** | **Placebo** | **Nangibotide** |
|  |  | n(%) | n(%) | n(%) | n(%) |
| Value | Clinical Status Definition |  | |  | |
| **1** | Not hospitalized, no limitations of activities | 0(0) | 3(7.7) | 0(0) | 0(0) |
| **2** | Not hospitalized, limitations of activities | 1 (3.2) | 4 (10.3) | 0(0) | 2 (9.1) |
| **3** | Hospitalized, not requiring supplemental oxygen | 5 (16.1) | 9 (23.1) | 3 (15.8) | 4 (18.2) |
| **4** | Hospitalized, requiring supplemental oxygen | 4 (12.9) | 3 (7.7) | 0(0) | 1 (4.5) |
| **5** | Hospitalized, on non-invasive ventilation or high flow oxygen devices | 0(0) | 0(0) | 0(0) | 0(0) |
| **6** | Hospitalized, on invasive mechanical ventilation or ECMO | 11 (35.5) | 12 (30.8) | 9 (47.4) | 10 (45.5) |
| **7** | Death | 10 (32.3) | 8 (20.5) | 7 (36.8) | 5 (22.7) |

Supplementary figure 1: Survival analysis in patients treated with dexamethasone plus nangibotide vs dexamethasone as the only immunomodulator.


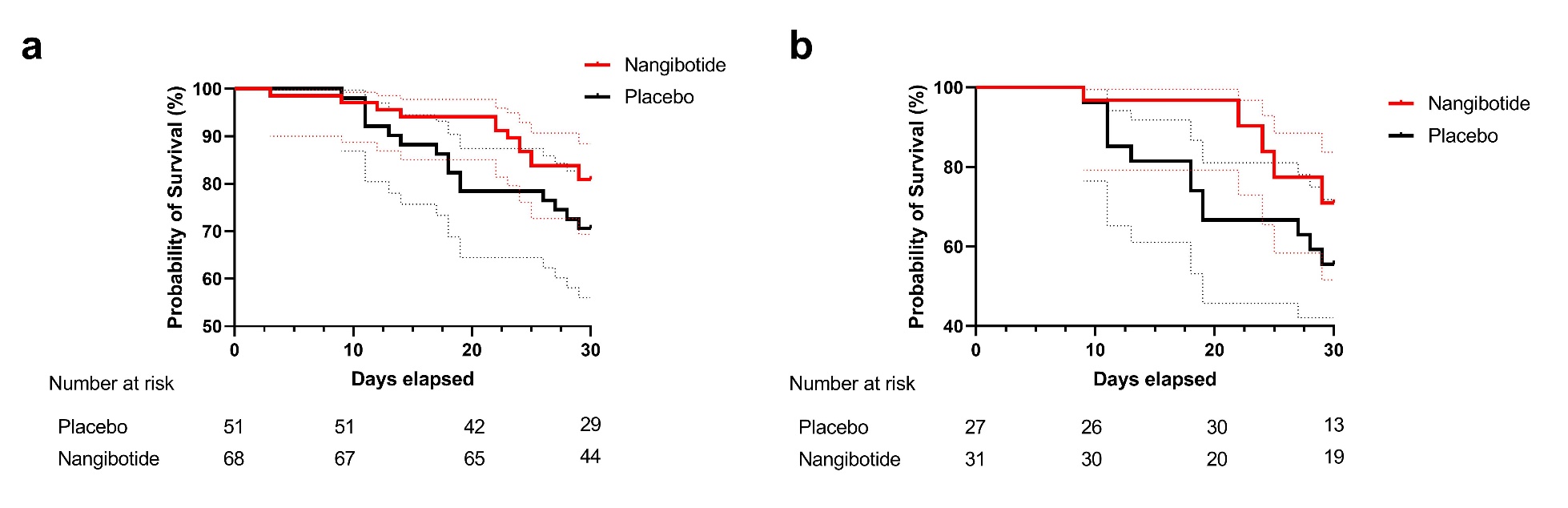


Supplementary figure 1: The impact of nangibotide on survival in patients that received dexamethasone as a single agent or in combination with nangibotide as treatment for COVID-19. **a**: overall and **b**: high sTREM-1 populations up to day 28 with 95% confidence intervals and the number of patients at risk at each timepoint reported.

Supplementary table 4 EQ5D-3L at day 60 - Overall population

|  |  | MOBILITY | | | |  | SELF-CARE | | | |  | USUAL ACTIVITIES | | | |  | PAIN/DISCOMFORT | | | |  | ANXIETY/ DEPRESSION | | | |
| --- | --- | --- | --- | --- | --- | --- | --- | --- | --- | --- | --- | --- | --- | --- | --- | --- | --- | --- | --- | --- | --- | --- | --- | --- | --- |
| Score |  | Placebo | | Nangibotide | |  | Placebo | | Nangibotide | |  | Placebo | | Nangibotide | |  | Placebo | | Nangibotide | |  | Placebo | | Nangibotide | |
| 1(n/%) |  | 27 | 56.3% | 34 | 65.4% |  | 35 | 72.9% | 43 | 82.7% |  | 22 | 45.8% | 21 | 40.4% |  | 22 | 46.8% | 24 | 46.2% |  | 29 | 61.7% | 29 | 55.8% |
| 2(n/%) |  | 16 | 33.3% | 17 | 32.7% |  | 10 | 20.8% | 7 | 13.5% |  | 19 | 39.6% | 27 | 51.9% |  | 23 | 48.9% | 27 | 51.9% |  | 14 | 29.8% | 21 | 40.4% |
| 3(n/%) |  | 5 | 10.4% | 1 | 1.9% |  | 3 | 6.3% | 2 | 3.8% |  | 7 | 14.6% | 4 | 7.7% |  | 2 | 4.3% | 1 | 1.9% |  | 4 | 8.5% | 2 | 3.8% |

Day 60 patient evaluation of functional status in each of the five components of the EQ5D -3L in the overall population. Patients (or immediate caregiver) reported a score of 1: no limitations, 2: some limitations or 3: severe limitation of functional status for each of the five categories. At the time of the evaluation, data from 48/56 surviving placebo and 52/59 surviving nangibotide treated patients was available.

Supplementary table 5: EQ5D-3L at day 60 - High sTREM-1 population

|  |  | MOBILITY | | | |  | SELF-CARE | | | |  | USUAL ACTIVITIES | | | |  | PAIN/DISCOMFORT | | | |  | ANXIETY/DEPRESSION | | | |
| --- | --- | --- | --- | --- | --- | --- | --- | --- | --- | --- | --- | --- | --- | --- | --- | --- | --- | --- | --- | --- | --- | --- | --- | --- | --- |
| Score |  | Placebo | | Nangibotide | |  | Placebo | | Nangibotide | |  | Placebo | | Nangibotide | |  | Placebo | | Nangibotide | |  | Placebo | | Nangibotide | |
| 1(n/%) |  | 9 | 50.0% | 10 | 55.6% |  | 12 | 66.7% | 13 | 72.2% |  | 8 | 44.4% | 5 | 27.8% |  | 8 | 47.1% | 9 | 50.0% |  | 13 | 76.5% | 10 | 55.6% |
| 2(n/%) |  | 6 | 33.3% | 8 | 44.4% |  | 4 | 22.2% | 4 | 22.2% |  | 5 | 27.8% | 10 | 55.6% |  | 8 | 47.1% | 9 | 50.0% |  | 3 | 17.6% | 7 | 38.9% |
| 3(n/%) |  | 3 | 16.7% | 0 | 0.0% |  | 2 | 11.1% | 1 | 5.6% |  | 5 | 27.8% | 3 | 16.7% |  | 1 | 5.9% | 0 | 0.0% |  | 1 | 5.9% | 1 | 5.6% |

Day 60 patient evaluation of functional status in each of the five components of the EQ5D -3L in the high sTREM-1 population. Patients (or immediate caregiver) reported a score of 1: no limitations, 2: some limitations or 3: severe limitation of functional status for each of the five categories. At the time of the evaluation, data from 18/24 (mobility, self-care, usual activities) and 17/24 (pain/Discomfort, Anxiety/Depression) surviving placebo and 18/22 surviving nangibotide treated patients was available.

Supplementary table 6: Per protocol exclusions

| **Patient Number** | **Activity Subtype** | **Description** |
| --- | --- | --- |
| 82002 | Study Treatment Compliance | Incorrect IMP vial allocation |
| 88001 | Inclusion Criteria | >48 hours respiratory support at randomisation |
| 90024 | Inclusion Criteria | No COVID-19 PCR available at inclusion |
| 90027 | Inclusion Criteria | No COVID-19 PCR available at inclusion, >48 hours respiratory support at randomisation |
| 90029 | Inclusion Criteria | No COVID-19 PCR available at inclusion |
| 90032 | Inclusion Criteria | >48 hours respiratory support at randomisation |
| 90035 | Inclusion Criteria | No COVID-19 PCR available at inclusion |
| 94004 | Exclusion Criteria | BMI>40 |
| 94007 | Inclusion Criteria | No COVID-19 PCR available at inclusion |
| 97015 | Inclusion Criteria | Severity of hypoxia inclusion criteria not met |
| 97017 | Inclusion Criteria | Severity of hypoxia inclusion criteria not met |
